# Theoretical Insights into the Gas-Phase Oxidation of 3-Methyl-2-butene-1-thiol by the OH Radical: Thermochemical and Kinetic Analysis

**DOI:** 10.1021/acs.jpca.3c07775

**Published:** 2024-03-11

**Authors:** Parandaman Arathala, Rabi A. Musah

**Affiliations:** Department of Chemistry, University at Albany−State University of New York, 1400 Washington Avenue, Albany, New York 12222, United States

## Abstract

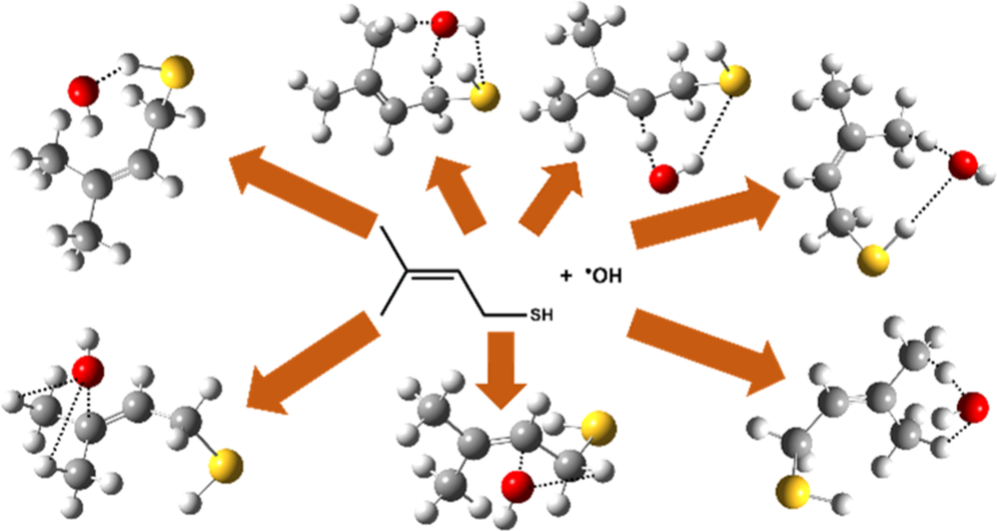

3-Methyl-2-butene-1-thiol ((CH_3_)_2_C=CH–CH_2_–SH; MBT) is a recently identified volatile organosulfur compound emitted from *Cannabis sativa* and is purported to contribute to its skunky odor. To understand its environmental fate, hydroxyl radical (^•^OH)-mediated oxidation of MBT was conducted using high-level quantum chemical and theoretical kinetic calculations. Three stable conformers were identified for the title molecule. Abstraction and addition pathways are possible for the MBT + OH radical reaction, and thus, potential energy surfaces involving H-abstraction and ^•^OH addition were computed at the CCSD(T)/aug-cc-pV(T+d)Z//M06-2X/aug-cc-pV(T+d)Z level of theory. The barrier height for the addition of the OH radical to a C atom of the alkene moiety, leading to the formation of a C-centered MBT–OH radical, was computed to be −4.1 kcal mol^–1^ below the energy of the starting MBT + OH radical-separated reactants. This reaction was found to be dominant compared to other site-specific H-abstraction and addition paths. The kinetics of all the site-specific abstraction and addition reactions associated with the most stable MBT + OH radical reaction were assessed using the MESMER kinetic code between 200 and 320 K. Further, we considered the contributions from two other conformers of MBT to the overall reaction of MBT + OH radical. The estimated global rate coefficient for the oxidation of MBT with respect to its reactions with the OH radical was found to be 6.1 × 10^–11^ cm^3^ molecule^–1^ s^–1^ at 298 K and 1 atm pressure. The thermodynamic parameters and atmospheric implications of the MBT + OH reaction are discussed.

## 1 Introduction

Investigation of the global sulfur cycle has been a major focus of interest because of the need to assess the contribution of biogenic sulfur required to balance the global sulfur cycle.^1^ Both natural and anthropogenic activities are responsible for the emissions of volatile organosulfur compounds (VOSCs) into the Earth’s atmosphere.^2^ Reduced organic sulfur compounds such as methanethiol (CH_3_SH), hydrogen sulfide (H_2_S), and dimethyl sulfide (CH_3_SCH_3_) as well as carbonyl sulfide (OCS) are the most common S-containing compounds released into the troposphere. Their transformation in the atmosphere can lead to climate modification, acid rain, and cloud formation.^3^ Methanethiol is the simplest VOSC and accounts for ∼10% of the global flux of sulfur-containing compounds.^4^ The atmospheric chemistry of biogenic sulfur compounds emitted from the oceans and their transformation processes are most well understood.^5,6^ However, detailed information on the sources and sinks of terrestrial biogenic sulfur species represents the largest uncertainty in the global atmospheric sulfur burden.^6^ Specifically, emission of sulfur species from living vascular plants and their transformation into others in the atmosphere are not well understood.^7^

Terrestrial plants release a variety of reactive organic compounds such as isoprene, terpenes, and oxygenated compounds, which make important contributions to the global budget of nonmethane volatile organic compounds.^8^ Plants also release a variety of VOSCs such as CS_2_, COS, methyl sulfide, dimethyl sulfide, and dimethyl disulfide into the atmosphere.^9^ Puxbaum and König^10^ reported that dipropenyl disulfide, methyl propenyl disulfide, diallyl sulfide, dimethyl disulfide, and 3-methylthiopropene were detected in the atmosphere of a beech forest with *Allium ursinum* ground cover plants. This study also reported the highest mean emission rate of 60 μg S m^–2^ h^–1^ for organic sulfur species emitted from a terrestrial plant.^10^ In addition, several alkyl thiosulfinates, namely, dimethyl thiosulfinate, dipropyl thiosulfinate, propyl methyl thiosulfinate, and diallyl thiosulfinate, are derived from *Allium* genus cash crops such as garlic and onions.^11,12^ These observations indicate that terrestrial plants may make significant contributions of organosulfur compounds into the Earth’s atmosphere, although the range and identities of these compounds continue to be discovered.

Recently, 3-methyl-2-butene-1-thiol ((CH_3_)_2_C=CH–CH_2_–SH; MBT) was detected in the emissions of the two varieties of *Cannabis sativa* species (i.e., hemp and marijuana) and was reported to be responsible for the skunky aroma of the plant.^13,14^ According to the Brightfield group, 288,000 acres of industrial hemp were cultivated in the U.S. in 2019.^15^ This indicates the large acreage occupied by *Cannabis* plants cultivated in the U.S. as well as throughout the world, and raises the question of whether the MBT emitted into the atmosphere from these plants may result in emergence of organosulfur hotspots that could have localized adverse environmental impacts on global warming and formation of acid rain, among others. Therefore, more information on the atmospheric transformation mechanism of this compound in the troposphere is required to assess its potential impact. Once released, MBT is expected to encounter and interact with hydroxyl (OH) radicals, as OH radicals are the most prominent reactive intermediates in atmospheric chemistry.^16,17^ In addition, previous studies have indicated that OH radicals play a dominant role in the oxidation of aliphatic thiols under tropospheric conditions.^3,18,19^

While there are several reports on the fate of saturated aliphatic thiols in the presence of OH radicals under atmospheric conditions, there are no data available on the oxidation of plant-derived unsaturated thiols initiated by OH radicals. Several experimental and theoretical studies have been devoted to the mechanism and products formed from the OH radical-mediated oxidation of saturated aliphatic thiols.^3,19−22^ For example, the kinetics of OH radical reactions with aliphatic thiols have been studied by Barnes et al.,^22^ Lee and Tang,^20^ and Wine et al.^3^ The results from these studies suggest that thiols react with OH radicals at nearly the same rate and mainly occur through H atom abstraction by OH radicals from the −SH group.^3,20,22^ In addition, a theoretical study of the gas phase reaction mechanism of C1–C3 aliphatic thiols with OH radicals has been reported,^18^ and the results suggested that H atom abstraction from the −SH group is the major contributor to the overall reaction compared to H atom abstraction from the alkyl groups. The rate coefficients for the reaction of CH_3_SH + OH radical, C_2_H_5_SH + OH radical, and *n-*C_3_H_7_SH + OH radical at 298 K are reported to be 1.3 × 10^–11^, 1.5 × 10^–11^, and 3.0 × 10^–11^ cm^3^ molecule^–1^ s^–1^, respectively. The kinetics of the reaction of OH radicals with CH_3_SH, C_2_H_5_SH, *n*-C_3_H_7_SH, and *iso*-C_3_H_7_SH have also been determined by theoretical methods over the temperature range of 252–430 K.^19^ The oxidation of thiols in the atmosphere has been found to ultimately lead to the generation of sulfur dioxide (SO_2_), which can undergo further oxidation to form sulfuric acid (H_2_SO_4_).^21^ This process contributes to particle formation and growth.^23,24^

Given that the discovery of the emission of MBT from *C. sativa* is very recent,^13,14^ there is a lack of information on the levels of this compound in the atmosphere. However, its detection in a terrestrial plant species that occupies large acreage by virtue of its widespread cultivation, raised the question of whether its presence in the atmosphere where emissions occur could lead to organosulfur hotspots that might have a more localized environmental impact. Thus, the observation of MBT presented the opportunity to consider the atmospheric implications of hydroxyl radical reactions with an unsaturated aliphatic thiol. Although the bimolecular rate coefficients for various aliphatic thiols with the OH radical have been documented, there is a notable gap in research concerning the reactive intermediates or stable products resulting from the interaction of unsaturated thiols in particular, with the OH radical.

Accordingly, the present work focused on determining the thermochemistry and kinetics for the initial OH radical addition and H-abstraction paths associated with the MBT + OH radical reaction. We studied the potential energy surfaces (PESs) of various possible channels in the MBT + OH radical reaction using high-level *ab initio*/DFT electronic structure calculations. The rate coefficients for all possible reactions were calculated in the atmospherically relevant temperatures between 200 and 320 K. The atmospheric implications for the reaction of MBT in the presence of OH radicals are presented. This information is important for its practical applications and more broadly in promoting an understanding of the fundamental mechanism of unsaturated thiol interactions with OH radicals.

## 2 Computational Methods

The hybrid density functional (M06-2X) along with the aug-cc-pV(T+d)Z basis set^25^ was used for geometry optimization for all the minima and transition states on the potential energy surface (PES) in the H atom abstraction and addition channels involved in the reaction of MBT with OH radicals. We added additional tight d-functions in the aug-cc-pVTZ basis set to facilitate better bonding in the S atom.^26,27^ The present theory and basis set combination have been shown to provide reliable stationary point geometries for simulating reaction mechanism and kinetic parameters.^28−30^ The optimized geometries of all the stationary points are provided in Table S1. The harmonic vibrational frequencies of all the minima and transition states were calculated using the same theory level. The vibrational frequencies, rotational constants of all the stationary points, and imaginary frequencies of the transition states computed in this work are displayed in Tables S2–S4, respectively. We found one imaginary frequency for the transition states and real frequencies for all the reactants, intermediates, and products in various possible elementary paths associated with the MBT + OH radical reaction. Intrinsic reaction coordinate (IRC) calculations^31,32^ were performed on each saddle point along the reaction path using the Hessian-based predictor corrector integrator algorithm to identify the corresponding prereactive and postreactive complexes at the same M06-2X/aug-cc-pV(T+d)Z level. Further, single-point energies at the CCSD(T)/aug-cc-pV(T+d)Z level were computed for all the stationary points on the PESs at the M06-2X/aug-cc-pV(T+d)Z level-optimized geometries to get precise energies. The final energies displayed on the PESs were calculated at the CCSD(T)/aug-cc-pV(T+d)Z//M06-2X/aug-cc-pV(T+d)Z (CCSD(T)//M06-2X) level, and zero-point energy (ZPE) correction was obtained at the M06-2X/aug-cc-pV(T+d)Z level. We previously used the combination of the dual level CCSD(T)//M06-2X approach (for the CH_3_S(O)_2_NH_2_ + OH radical and CH_3_S(O)_2_CH_3_ + OH radical reaction systems), as it predicted energies and kinetic features similar to experimentally measured values.^28,33^ The computed single-point energies, ZPEs, thermal correction to enthalpy, and Gibbs free energy for all the stationary points obtained at various levels are displayed in Table S5. The present electronic structure calculations were performed with the Gaussian 16 software suite.^34^ The T1 diagnostic values for all the minima and transition states were calculated using the CCSD(T)/aug-cc-pV(T+d)Z method. The results indicated that the T1 diagnostic values fell between 0.01 and 0.022 for all the species in the presently studied reaction paths (see Table S6). This suggests that the multireference character of the wave function of all the stationary points is not significant.^35,36^ Thus, the present calculated energies for all the stationary points are considered to be reliable.

## 3 Results and Discussion

### 3.1 Conformational Analysis

**Figure 1:**
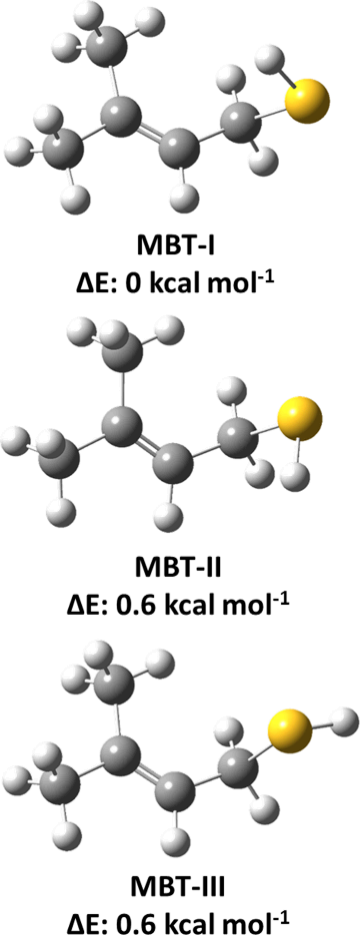
Fully optimized geometries and energies of the MBT conformers obtained at the CCSD(T)/aug-cc-pV(T+d)Z//M06-2X/aug-cc-pV(T+d)Z level. The energies of the conformers MBT-II and MBT-III were calculated relative to that of MBT-I. The black, yellow, and white colors denote C, S, and H atoms, respectively.

The most stable conformer of MBT is required to investigate the mechanism of its OH radical-mediated oxidation under atmospherically relevant conditions. Therefore, we performed conformational analysis at the M06-2*X*/6-311++G(2d,2p) level. The structure of MBT has four internal rotational degrees of freedom, namely, three C–C and one C–S bond torsions. This calculation revealed several different minima, and all of them were fully optimized at the M06-2X/aug-cc-pV(T+d)Z level. Moreover, we refined the energies by calculating the single-point energies for all of the conformers at the CCSD(T)/aug-cc-pV(T+d)Z level. The three most stable conformations of MBT with their corresponding energies are shown in Figure 1. The structures of these three conformers (designated as MBT-I, MBT-II, and MBT-III; see Figure 1) differ only in terms of the orientation of the H atom in the −SH group of MBT. The energies of the higher energy conformers were calculated relative to the energy of the most stable conformer (MBT-I). The ZPE-corrected CCSD(T)//M06-2X level-estimated energies indicated that the structure of MBT-I is more stable by ∼0.6 kcal mol^–1^ relative to the structures of both MBT-II and MBT-III (see Figure 1). The energy difference between these conformers is very small (∼0.6 kcal mol^–1^), and hence, all three stable conformations are expected to contribute to the overall MBT + ^•^OH reaction. We initiated our present investigation of the atmospheric transformation of MBT with OH radicals using the most stable structure (MBT-I).

### 3.2 Initial Reaction Pathways of MBT-I + OH Radical

**Figure 2:**
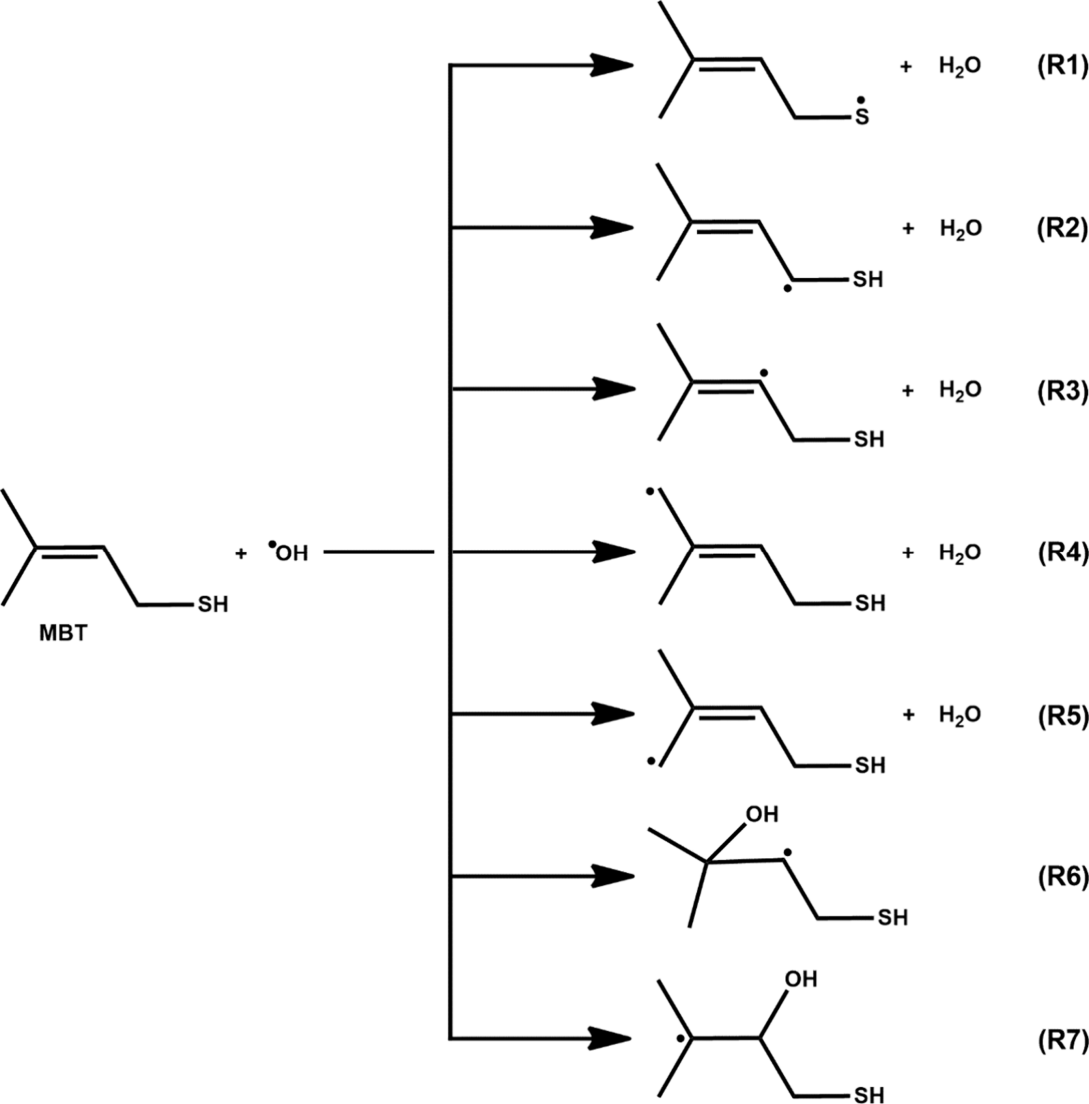
Possible paths for the reaction of MBT with OH radical, featuring H-abstraction to form S- and C-centered MBT radicals + water (R1–R5) and OH radical addition to one of the two carbons of the double bond to form two different C-centered MBT–OH addition products (R6 and R7).

The initial attack by the OH radical on MBT-I in the atmosphere can proceed via abstraction and/or addition pathways. Specifically, H atom abstraction can occur at the −CH_2_, −CH, −SH, and two −CH_3_ groups, while addition of the OH radical can occur at either of the C atoms of the alkene double bond. This suggests a total of five abstraction and two addition paths as illustrated in Figure 2. Reaction path R1 shows abstraction of a H atom from the −SH site by the OH radical to form a S-centered MBT radical and H_2_O molecule. Reaction paths R2, R3, R4, and R5 illustrate OH radical-initiated H atom abstraction from −CH_2_, =CH, and two −CH_3_ sites to form the corresponding C-centered MBT radical + H_2_O (see Figure 2). Reaction paths R6 and R7 show OH radical addition to either of the C atoms of the double bond. This leads to formation of the corresponding C-centered MBT–OH radical products (see Figure 2).

**Figure 3:**
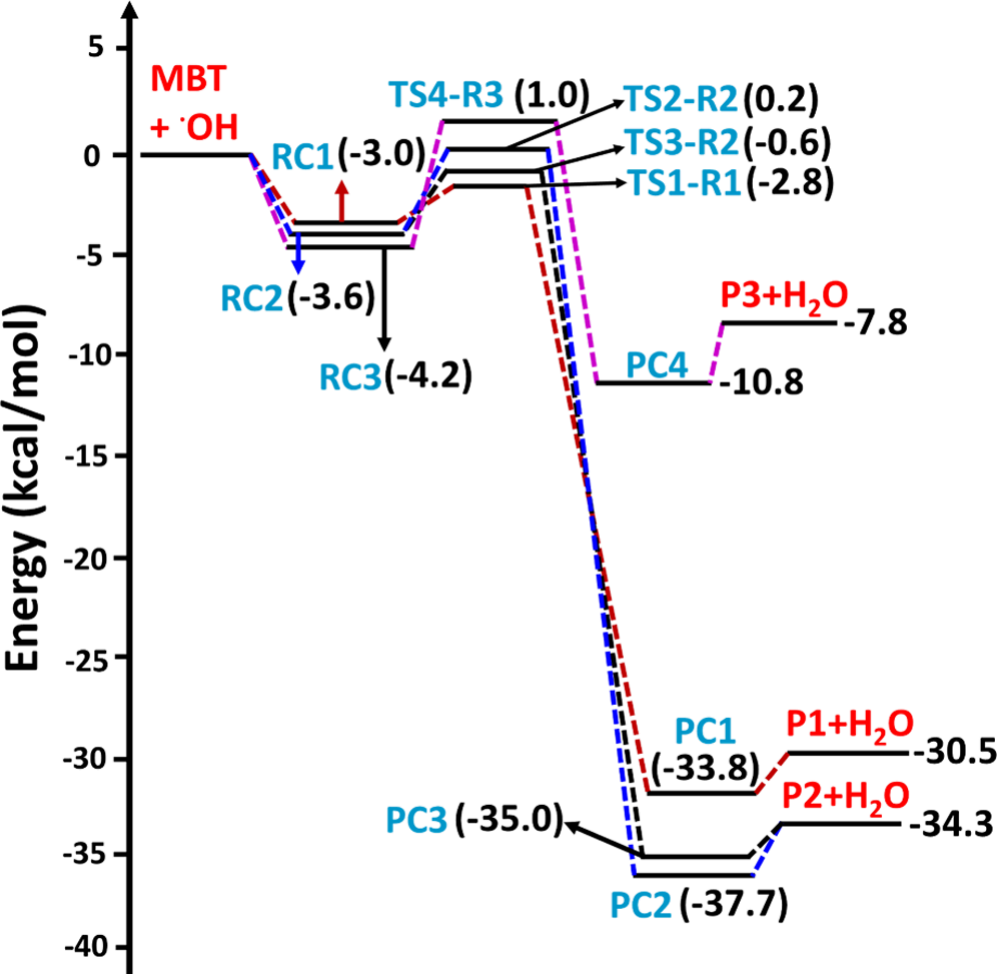
Potential energy surface profiles for H-abstraction from the −SH, −CH_2_, and =CH sites for the MBT + OH radical reaction computed at the CCSD(T)/aug-cc-pV(T+d)Z//M06-2X/aug-cc-pV(T+d)Z level. The RCs, TSs, PCs, and Ps refer to prereactive complexes, transition states, product complexes, and products, respectively.

**Figure 4:**
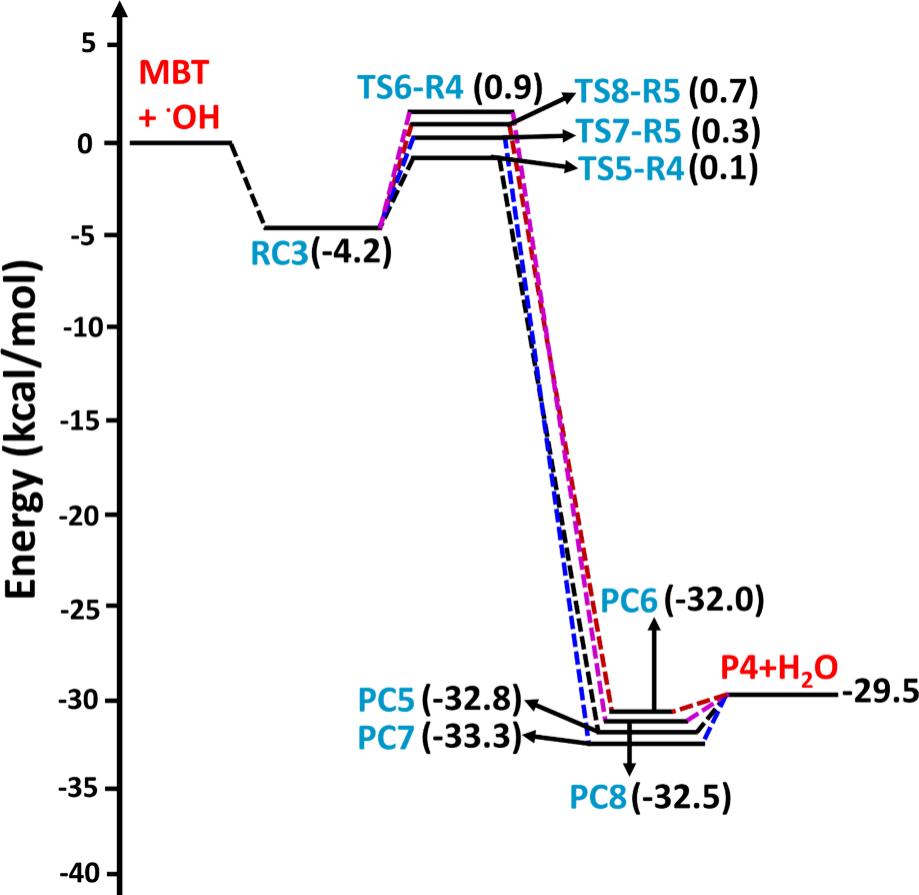
Potential energy surface profiles for H-abstraction from the two methyl groups of MBT by the OH radical, computed at the CCSD(T)/aug-cc-pV(T+d)Z//M06-2X/aug-cc-pV(T+d)Z level. The RCs, TSs, PCs, and Ps refer to prereactive complexes, transition states, product complexes, and products, respectively.

**Figure 5:**
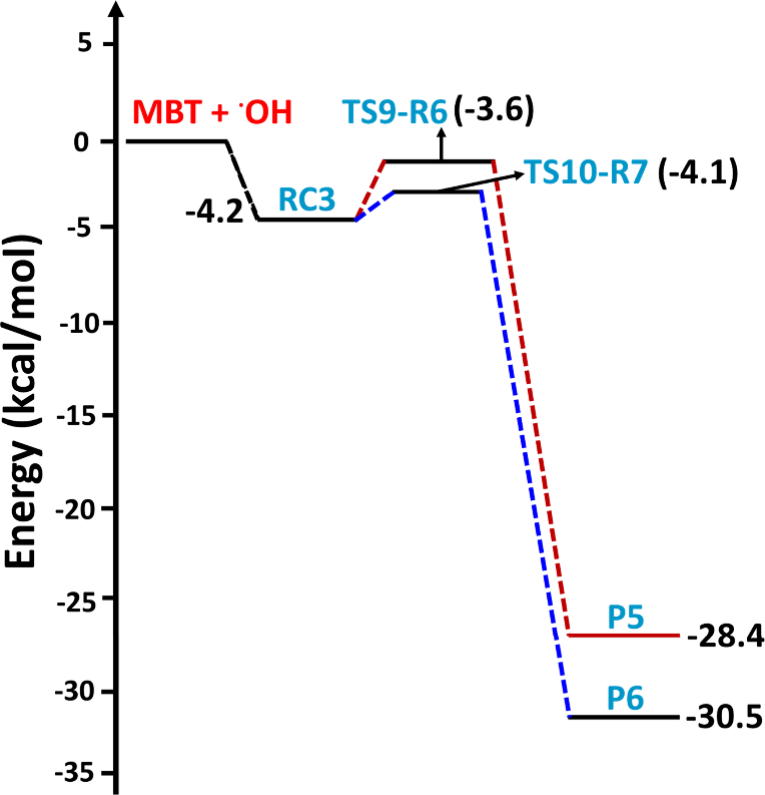
Potential energy surface profiles for the two possible paths associated with addition of the OH radical to either of the two carbons of the double bond of MBT, computed at the CCSD(T)/aug-cc-pV(T+d)Z//M06-2X/aug-cc-pV(T+d)Z level. The RCs, TSs, and Ps refer to prereactive complexes, transition states, and products, respectively.

The potential energy surface profiles for the H-abstraction from the −SH, −CH_2_, and =CH sites of MBT by the OH radical, and OH radical-mediated abstraction from either of the methyl groups, computed at the CCSD(T)/aug-cc-pV(T+d)Z//M06-2X/aug-cc-pV(T+d)Z level, are shown in Figures 3 and 4, respectively. The potential energy surface profiles, computed at the same level, for the addition of the OH radical to each of the two carbons of the double bond to form the corresponding C-centered radicals, are shown in Figure 5. For these computations, the energies of all the minima and transition states in the PES profiles were calculated relative to the separated reactants (i.e., MBT + OH radical) computed at the CCSD(T)//M06-2X level. The results reveal that for paths involving H atom abstraction, the reaction process proceeds as follows: (1) association of the two reactants (MBT and OH radical), leading to formation of barrierless prereactive complexes (RCs); (2) formation of transition states involving the intermediate prereactive complexes (RCs) and product complexes (PCs); and (3) formation of the separated products (S- and C-centered MBT radicals + H_2_O) from decomposition of the postreactive complexes (PCs). For the addition paths, the mechanism proceeds via steps (1) and (2) to form the products (see Figure 5).

**Figure 6:**
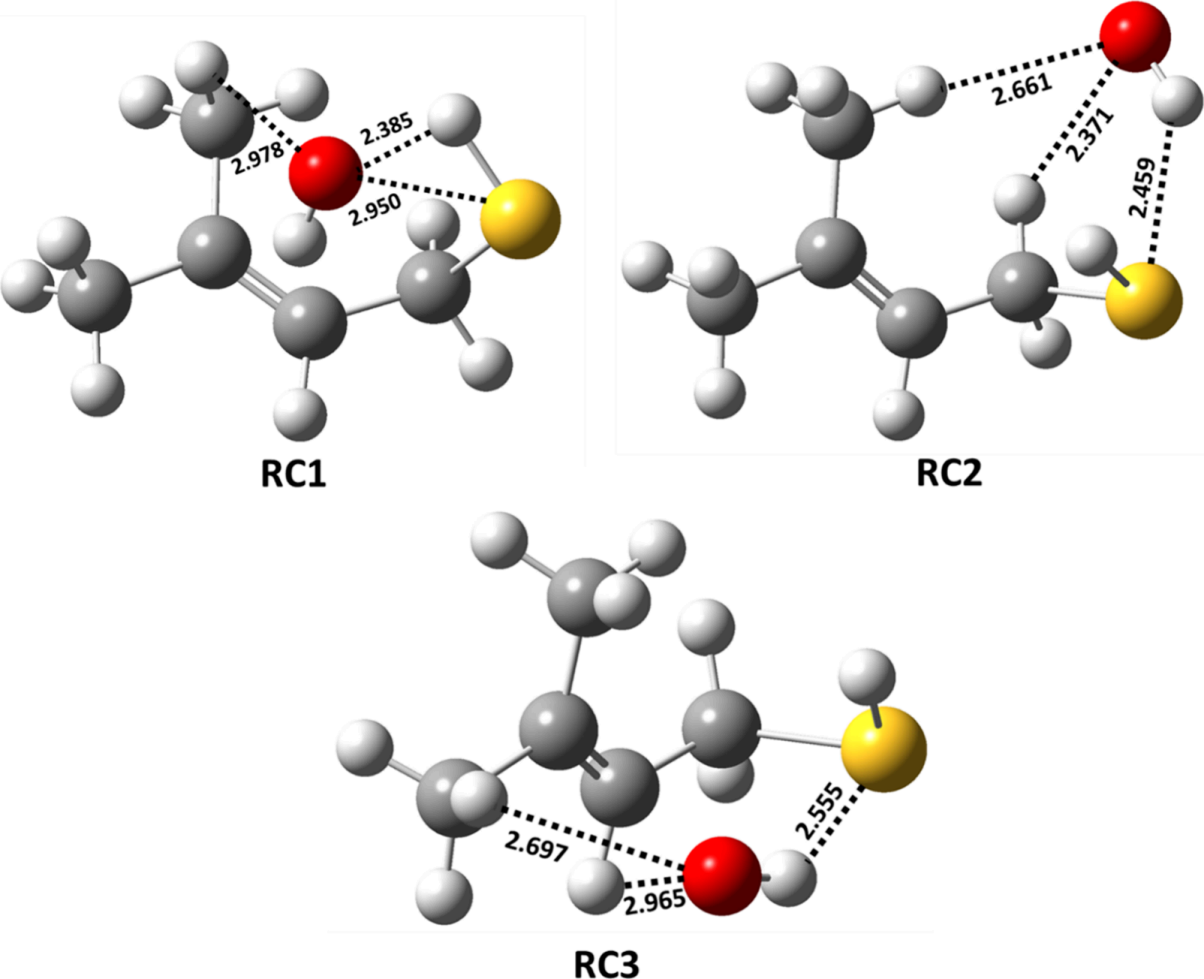
Optimized geometries of the prereactive complexes involved in the MBT + ^•^OH reaction obtained at the M06-2X/aug-cc-pV(T+d)Z level. The black, yellow, red, and white colors denote C, S, O, and H atoms, respectively.

The PES profiles shown in Figure 3 suggest that the initial encounter of the two reactants results in formation of prereactive complexes (RC1, RC2, and RC3) with corresponding binding energies of −3.0, −3.6, and −4.2 kcal mol^–1^ below that of the starting reactants for the hydrogen bonding-stabilized orientations that lead to abstraction of the H atom from sulfur, the carbon adjacent to sulfur (i.e., the α-carbon), and the β-carbon, respectively. The structures of these three prereactive complexes were computed using IRC calculations, and they are shown in Figure 6. The prereactive complex (RC1) involved in the abstraction of the H atom from the −SH group shows an interaction between the O atom in the OH radical and the S atom with a bond length of 2.95 Å. A similar prereactive complex was reported for the abstraction of a H atom by the OH radical from the −SH group of C1–C3 aliphatic thiols, with binding energies that varied between 2.0 and 2.6 kcal mol^–1^ below that of their corresponding separated reactants at the CCSD(T)/6-311++G(d,p)//BHandHLYP/6-311++G(2d,2p) level.^18^ The binding energy of RC1 in the present work is ∼0.5 kcal mol^–1^ greater than the reported values for the analogous reaction complexes in the C1–C3 aliphatic thiols. This may be due to the two additional interactions present in RC1, namely, that between the H atom of the −SH group and the O atom in the OH radical (with a bond length of 2.39 Å) and that between the O atom in the OH radical and the −H atom in the methyl group (with a bond length of 2.98 Å). We found three different hydrogen bonding interactions for the orientations leading to H atom abstraction from the −CH_2_ group of MBT (see the structure of RC2 in Figure 6). Two interactions were between one of the H atoms of the −CH_2_ and −CH_3_ groups and the O atom in the OH radical (with bond lengths of 2.66 and 2.37 Å, respectively). The third interaction was between the S atom and the H atom of the OH radical (with a bond length of 2.46 Å). For the reactant interactions leading to H atom abstraction from =CH and either of the two −CH_3_ moieties, as well as for the two OH radical addition pathways leading to the corresponding C-centered radicals, the prereactive complex (RC3) was formed through hydrogen bonding interactions between (a) the O atom of the OH radical and the H atom of =CH, (b) the O atom of the OH radical and a H atom of −CH_3_, and (c) the H atom of the OH radical and the S atom of the −SH group, with corresponding bond lengths of 2.70, 2.97, and 2.56 Å, respectively (see Figure 6). The results suggest that RC3 is the most stable by 0.6–1.2 kcal mol^–1^ compared to the values of RC1 and RC2.

**Figure 7:**
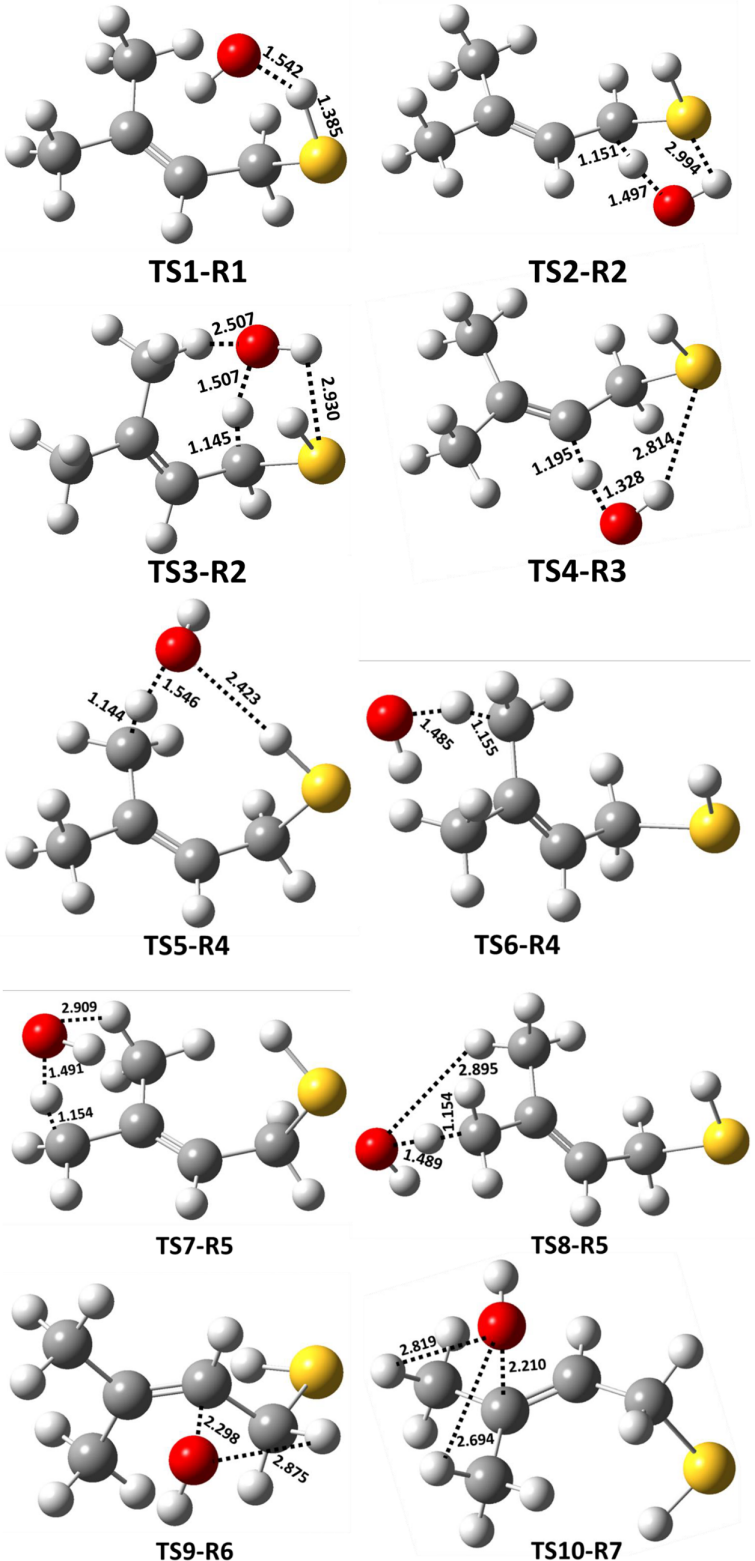
Optimized geometries of transition states for the H-abstraction and addition paths associated with the MBT + ^•^OH reaction obtained at the M06-2X/aug-cc-pV(T+d)Z level. The black, yellow, red, and white colors denote C, S, O, and H atoms, respectively.

The structures of all the transition states, postreactive complexes, and products for various possible abstraction and addition paths are shown in Figure 7 and Figure S1. Bond formation and bond cleavage in the transition state structures are illustrated with dotted lines (see Figure 7). Transition state TS1-R1 was formed from RC1 for the abstraction of a H atom from the −SH group of MBT. The barrier height for TS1-R1 was found to be −2.8 kcal mol^–1^ below that of the starting MBT and OH radical reactants(see Figure 3). We noted that the transition state barrier heights for the abstraction of a H atom from the −SH group of CH_3_SH by the OH radical were reported to be −1.1 kcal mol^–1^ at the CCSD(T)/aug-cc-pV(T,Q)Z//M06-2X/aug-cc-pV(T+d)Z level^37^ and −0.2 kcal mol^–1^ at the CCSD(T)/6-311++G(d,p)//BHandHLYP/6-311++G(2d,2p) level.^18^ This suggests that the TS1-R1 barrier is 1.7 and 2.6 kcal mol^–1^ lower than the analogous transition state for the CH_3_SH + OH radical reaction. H-abstraction from the −SH group of the higher alkanethiol analogues (i.e., C_2_H_5_SH and *n*-C_3_H_7_SH) with the OH radical has been reported to be −0.3 and −0.4 kcal mol^–1^ below that of their respective starting reactants when computed at the CCSD(T)/6-311++G(d,p)//BHandHLYP/6-311++G(2d,2p) level.^18^ Thus, these results further suggest that the TS1-R1 barrier is lower by 2.5 and 2.4 kcal mol^–1^ compared to the analogous reactions of C2–C3 thiols with OH radicals. The lower barrier for TS1-R1 may be due to interactions between the OH radical H atom with the electron density of the alkene group (see Figure 7), which might stabilize the transition state, as this type of interaction is absent in C1–C3 alkanethiols. This abstraction path was further found to form a stable postreactive complex (PC1) on the reaction coordinate, which occurs through hydrogen bonding interactions between a water molecule and the S-centered MBT radical (P1; (CH_3_)_2_C=CHCH_2_S^•^) at −33.8 kcal mol^–1^. Finally, the bimolecular separated products (P1 + H_2_O) are formed from PC1 at −30.5 kcal mol^–1^ below the reactants. Transition states TS2-R2 and TS3-R2 are formed from RC2 for the abstraction of a H atom from the −CH_2_ group of MBT, with respective barrier heights of 0.2 and −0.6 kcal mol^–1^ above and below the reactants respectively. Even though the OH radical abstracts a H atom from the same C atom via TS2-R2 and TS3-R2, the energy of TS3-R2 was found to be more stable by 0.8 kcal mol^–1^ compared to the energy of TS2-R2. This may be due to the presence of an additional hydrogen bonding interaction occurring between a H atom of the methyl group in MBT and the O atom in the OH radical, with a bond length of 2.51 Å (see the structure TS3-R2 in Figure 7). Once formed, TS2-R2 and TS3-R2 further lead to PC2 and PC3 with energies of −37.7 and −35.0 kcal mol^–1^ below the reactants and then to the same separated products (P2: ((CH_3_)_2_C=CHC^•^HSH) + H_2_O) at −34.3 kcal mol^–1^ below the reactants. The abstraction of a H atom at the =CH group of MBT by the OH radical proceeds from RC3 to TS4-R3 with a barrier height of 1.0 kcal mol^–1^ above the reactants. TS4-R3 forms PC4 and then P3 ((CH_3_)_2_C=C^•^CH_2_SH) + H_2_O separated products at −7.8 kcal mol^–1^ on the PES profile. The results presented in Figure 3 reveal that abstraction of the H atom from the −SH group of MBT is the energetically dominant path. Transition states TS5-R4, TS6-R4, TS7-R5, and TS8-R5, formed via abstraction of a H atom from the methyl groups by the OH radical, were formed from the same prereactive complex (RC3) with barrier heights of 0.1, 0.9, 0.3, and 0.7 kcal mol^–1^, respectively, above the reactants(see Figure 4). These transition states undergo formation of the corresponding PC5, PC6, PC7, and PC8 on the reaction coordinate, with energies of −32.8, −32.0, −33.3, and −32.5 kcal mol^–1^, respectively, and then proceed to the same products (P4: (CH_3_)(C^•^H_2_)C=CHCH_2_SH) + H_2_O, with a potential energy of −29.5 kcal mol^–1^ below that of the reactants. Based on the PES profiles, abstraction of the H atom from the −SH group of MBT by the OH radical is energetically favored compared to the other possible H-abstraction paths.

By virtue of the alkene functional group contained within MBT, OH radical addition can occur at either of the C atoms of the double bond (paths R6 and R7 in Figure 2). We found that these two addition paths form the same RC (i.e., RC3) and lead to their corresponding MBT–OH addition transition states (TS9-R6 and TS10-R7) with computed barrier heights of −3.6 and −4.1 kcal mol^–1^ relative to the starting reactants, respectively (see Figure 5). In both processes, a new single bond between the O atom from the OH radical and carbon is formed while simultaneously breaking the double bond and generating a new C-centered radical (see the structures of TS9-R6 and TS10-R7 in Figure 7). The two barrier heights of −3.6 and −4.1 kcal mol^–1^ relative to the starting reactants indicate that the transition states are below the separated reactants, and thus, the two paths occur in a barrierless manner. Moreover, TS9-R6 and TS10-R7 lead to formation of two different C-centered MBT–OH radicals as products (P5 and P6) on the PES at −28.4 and −30.5 kcal mol^–1^ below the separated reactants (see Figure 5). The results from the PES profiles shown in Figures 3–5 indicate that the addition of the OH radical via TS10-R7 to form P6 ((CH_3_)_2_C(OH)C^•^HCH_2_SH) is energetically more dominant compared to the other possible abstraction and addition paths studied in the title reaction.

We also calculated the thermodynamic parameters (i.e., Gibbs free energy and enthalpy) at 298 K for all the minima and saddle points on the PESs associated with various reaction pathways in the title reaction at the CCSD(T)//M06-2X level. The obtained values are displayed in Table 1, and they are referenced to the starting reactants (MBT + OH radical). The enthalpies and Gibbs free energy data presented in Table 1 indicate that, of the H-atom abstraction possibilities, the one involving abstraction of an H-atom from the −SH group via TS1-R1 is energetically favored, with values of −3.6 and 5.6 kcal mol^–1^ below and above the starting reactants, respectively. Additionally, the enthalpies and Gibbs free energy associated with other potential H-abstractions via TS2-R2 to TS8-R5 are characterized by Δ*H* > −3.6 kcal mol^–1^ and Δ*G* > 5.6 kcal mol^–1^, respectively (see Table 1). This indicates that the H-abstraction via TS1-R1 is predominant compared to other possible abstraction channels. The computed enthalpy and Gibbs free energy values in Table 1 further indicate the exothermic and spontaneous nature of this reaction pathway, with values of −30.6 and −31.2 kcal mol^–1^, respectively.

**Table 1:** Enthalpies (Δ*H* (298 K)) and Free Energies (Δ*G* (298 K)) of Various Stationary Points Involved in the Reaction of MBT-I + OH Radical, Calculated at the CCSD(T)/aug-cc-pV(T+d)Z//M06-2X/aug-cc-pV(T+d)Z Level^a^

**system**	**Δ***H***(298 K)(**kcal mol^–1^)	**Δ***G***(298 K)(**kcal mol^–1^)
MBT-I + ^•^OH	0.0	0.0
RC1	–3.3	4.7
TS1-R1	–3.6	5.6
PC1	–33.7	–26.7
P1 + H_2_O	–30.6	–31.2
RC2	–3.8	3.8
TS2-R2	–0.3	7.9
PC2	–37.2	–31.1
P2 + H_2_O	–34.0	–35.1
TS3-R2	–1.5	8.1
PC3	–35.1	–27.1
RC3	–4.7	4.0
TS4-R3	0.2	9.6
PC4	–10.5	–4.2
P3 + H_2_O	–7.4	–9.6
TS5-R4	–0.6	7.7
PC5	–32.7	–26.3
P4 + H_2_O	–29.3	–30.5
TS6-R4	0.1	8.6
PC6	–31.8	–25.1
TS7-R5	–0.2	7.1
PC7	–33.4	–25.4
TS8-R5	0.1	8.2
PC8	–32.3	–25.5
RC4	–3.2	–3.8
TS9-R6	–4.2	3.9
P5	–29.4	–19.5
TS10-R7	–4.9	4.5
P6	–31.8	–20.6

a The enthalpic (*H*) and free energy (*G*) corrections were derived from M06-2X/aug-cc-pV(T+d)Z level calculations.

The computed enthalpies for the formation of TS9-R6 and TS10-R7 were found to be −4.2 and −4.9 kcal mol^–1^, with corresponding Gibbs free energies of 3.9 and 4.5 kcal mol^–1^, respectively. These results indicate that the addition pathways are more favorable than the abstraction pathways. Additionally, the computed enthalpy and Gibbs free energy data for these two reaction pathways (which lead to products P5 and P6) are exothermic by −29.4 and −31.8 kcal mol^–1^, respectively, and spontaneous by −19.5 and −20.6 kcal mol^–1^, respectively.

We found that structures MBT-II and MBT-III (see Figure 1) are ∼0.6 kcal mol^–1^ less stable than the structure of MBT-I. The small energy difference between the conformers suggests that structures MBT-II and MBT-III also contribute to the overall MBT + OH radical reaction. We considered the structure MBT-II (see Figure 1) for further mechanistic and kinetic calculations to estimate the contribution from this conformer to the overall reaction. Like the most stable conformer, MBT-II can also undergo five H atom abstractions and two OH addition paths (see Figure 2). All the minima and saddle points involved in these abstraction and addition paths were fully optimized at the same M06-2X/aug-cc-pV(T+d)Z level. However, for the sake of brevity, the discussion of their geometries is not provided here. We used the same methodology provided in Section 2 for IRC calculations conducted at the M06-2X/aug-cc-pV(T+d)Z level. Additionally, CCSD(T)//M06−2X level energy calculations were performed for the stationary points for all possible paths for this conformer in its reactions with OH radical. The PES profiles for abstraction and addition paths associated with the reaction of MBT-II + OH radical are shown in Figures S2–S4. Based on the PES profiles in Figures S2 and S3, the abstraction of a H atom from the −SH group by the OH radical proceeds via RC4, TS11-R1, and PC11 to form products P1 + H_2_O. We found that the two H atoms in the −CH_2_ group are equivalent in this conformer, resulting in one transition state. This abstraction path proceeds via RC5, TS12-R2, and PC12 to form P2 + H_2_O products. The abstraction of a H atom from the =CH group by the OH radical leads to the formation of RC6, TS13-R3, PC13, and P3 + H_2_O as products. The H atom abstraction from the two different methyl groups primarily proceeds to the formation of two prereactive complexes (RC7 and RC8). Once formed, RC7 then leads to the formation of TS14-R4 and TS16-R5 and then to PC14 and PC16 to result in P4 + H_2_O as products. Similarly, RC8 forms TS15-R4 and TS17-R5, which proceed to form PC15 and PC17, resulting in the same products (P4 + H_2_O). The two possible addition paths initially form RC9, which leads to TS18-R6 and TS19-R7 and ultimately to the products P5 and P6, respectively. Based on the results, the abstraction of a H atom by the OH radical from the −SH group of MBT-II via TS11-R1 with a barrier height of −1.1 kcal mol^–1^ to form the corresponding S-centered MBT radical + H_2_O products is a major path when compared to other possible H-abstraction paths. The results from the PES profiles shown in Figure S4 indicate that addition of the OH radical to a C atom of the alkene group via TS19-R7 with a barrier height of −3.8 kcal mol^–1^ below the isolated reactants, leading to the formation of (CH_3_)_2_C(OH)C^•^HCH_2_SH (P6), represents the most energetically favorable reaction compared to the other possible abstraction and addition paths.

## 4 Reaction Kinetics

The Master Equation Solver for Multi-Energy well Reactions (MESMER) kinetic code^38^ employs master equation simulations to calculate rate coefficients, and it was applied to all potential H atom abstraction and addition pathways within the title reaction. This kinetic code utilizes the energy-grained master equation framework, as discussed in our prior works.^39,40^ A concise overview of MESMER and a comprehensive explanation of how it was applied in this work are described in the Supporting Information.

The temperature-dependent rate coefficients for various possible abstraction and addition pathways associated with the title reaction were calculated in the temperature range of 200 to 320 K and at a pressure of 1 atm. The results are provided in Table 2. We observed a negative temperature dependence trend, indicating that the rate coefficients decrease with increasing temperature, for the reaction paths proceeding via TS1-R1, TS2-R2, TS3-R2, TS4-R3, TS5-R4, TS6-R4, TS9-R6, and TS10-R7 within the temperature range of 200 to 320 K (see Table 2). In contrast, the rate coefficients for the other paths via TS7-R5 and TS8-R5 were found to decrease initially and then gradually increase with temperature within the studied temperature range. The negative temperature dependence trend for the reaction paths is mainly due to having been modeled with the formation of prereactive complexes from the starting reactants. The formation of a prereactive complex often is accompanied by a negative temperature dependence of the bimolecular reaction rate due to the need for the molecules to correctly orient themselves before the reaction.^41,42^ The investigation of prereactive complexes has been thorough, particularly in relation to the OH reaction involving formic acid, ethene, acetone, and acetaldehyde.^41−44^ These studies have revealed the significance of prereactive complexes, which are characterized by bimolecular reaction rate coefficients exhibiting a negative temperature dependence. The data from Table 2 suggest that the rate coefficient for the abstraction of a H atom from the −SH group by the OH radical via TS1-R1 is the major reaction compared to all possible H-abstraction channels. The calculated rate coefficients within the temperature range of 200 to 320 K differ by approximately an order of magnitude larger compared to the values for other possible H-abstraction channels. For example, at 298 K, the computed rate coefficient for the H-abstraction reaction via TS1-R1 was found to be 5.3 × 10^–12^ cm^3^ molecule^–1^ s^–1^, whereas, at the same temperature, the rate coefficients for the other H-abstractions via TS2-R2, TS3-R2, TS4-R3, TS5-R4, TS6-R4, TS7-R5, and TS8-R5 were 6.6 × 10^–13^, 5.4 × 10^–13^, 1.1 × 10^–13^, 7.8 × 10^–13^, 2.8 × 10^–13^, 2.4 × 10^–12^, and 4.7 × 10^–13^ cm^3^ molecule^–1^ s^–1^, respectively.

**Table 2:** Rate Coefficients (in cm^3^ molecule^–1^ s^–1^) for the Abstraction and Addition Paths Associated with the MBT-I + OH Radical Reaction in the 200–320 K Temperature Range and at 1 atm Pressure

***T* (K)**	**TS1-R1**	**TS2-R2**	**TS3-R2**	**TS4-R3**	**TS5-R4**^a^	**TS6-R4**	**TS7-R5**
200	7.07 × 10^–12^	8.53 × 10^–13^	1.11 × 10^–12^	3.29 × 10^–13^	1.03 × 10^–12^	4.60 × 10^–13^	2.65 × 10^–12^
220	6.69 × 10^–12^	7.44 × 10^–13^	9.04 × 10^–13^	2.28 × 10^–13^	9.15 × 10^–13^	3.65 × 10^–13^	2.51 × 10^–12^
240	6.30 × 10^–12^	6.86 × 10^–13^	7.64 × 10^–13^	1.70 × 10^–13^	8.46 × 10^–13^	3.15 × 10^–13^	2.42 × 10^–12^
250	6.11 × 10^–12^	6.69 × 10^–13^	7.10 × 10^–13^	1.51 × 10^–13^	8.23 × 10^–13^	3.00 × 10^–13^	2.40 × 10^–12^
260	5.92 × 10^–12^	6.59 × 10^–13^	6.65 × 10^–13^	1.37 × 10^–13^	8.07 × 10^–13^	2.90 × 10^–13^	2.38 × 10^–12^
280	5.56 × 10^–12^	6.51 × 10^–13^	5.92 × 10^–13^	1.17 × 10^–13^	7.87 × 10^–13^	2.80 × 10^–13^	2.38 × 10^–12^
298	5.25 × 10^–12^	6.55 × 10^–13^	5.42 × 10^–13^	1.06 × 10^–13^	7.82 × 10^–13^	2.78 × 10^–13^	2.40 × 10^–12^
300	5.22 × 10^–12^	6.56 × 10^–13^	5.37 × 10^–13^	1.05 × 10^–13^	7.81 × 10^–13^	2.79 × 10^–13^	2.40 × 10^–12^
320	4.90 × 10^–12^	6.71 × 10^–13^	4.97 × 10^–13^	9.83 × 10^–14^	7.85 × 10^–13^	2.84 × 10^–13^	2.44 × 10^–12^

**TS8-R5**^a^	**TS9-R6**	**TS10-R7**	*k*_MBT-I_^b^	***X***_**1**_^c^	***k***_1_^d^	***k***_**global**_^e^
6.11 × 10^–13^	9.20 × 10^–12^	1.69 × 10^–10^	1.94 × 10^–10^	0.74672	1.45 × 10^–10^	1.54 × 10^–10^
5.21 × 10^–13^	9.22 × 10^–12^	1.37 × 10^–10^	1.60 × 10^–10^	0.71508	1.15 × 10^–10^	1.24 × 10^–10^
4.78 × 10^–13^	9.23 × 10^–12^	1.10 × 10^–10^	1.33 × 10^–10^	0.68696	9.12 × 10^–11^	1.01 × 10^–11^
4.67 × 10^–13^	9.22 × 10^–12^	9.91 × 10^–11^	1.21 × 10^–10^	0.67412	8.17 × 10^–11^	9.14 × 10^–11^
4.61 × 10^–13^	9.21 × 10^–12^	8.91 × 10^–11^	1.11 × 10^–10^	0.66203	7.34 × 10^–11^	8.33 × 10^–11^
4.60 × 10^–13^	9.18 × 10^–12^	7.23 × 10^–11^	9.36 × 10^–11^	0.63990	5.99 × 10^–11^	7.00 × 10^–11^
4.69 × 10^–13^	9.15 × 10^–12^	6.02 × 10^–11^	8.11 × 10^–11^	0.62210	5.04 × 10^–11^	6.08 × 10^–11^
4.70 × 10^–13^	9.14 × 10^–12^	5.90 × 10^–11^	7.98 × 10^–11^	0.62023	4.95 × 10^–11^	5.99 × 10^–11^
4.88 × 10^–13^	9.09 × 10^–12^	4.85 × 10^–11^	6.90 × 10^–11^	0.60268	4.16 × 10^–11^	5.22 × 10^–11^

a The degeneracy of TS5-R4 and TS8-R5 is 2, and thus, the contribution of the rate coefficients through TS5-R4 and TS8-R5 was multiplied by a factor of 2.

b *k*_MBT-I_ is the total rate coefficient for the MBT-I + OH radical reaction obtained using the corresponding site-specific reaction rate coefficients at each temperature.

c Mole fraction of MBT-I calculated using eq 2.

d The total rate coefficient (*k*_1_) was calculated by multiplying *k*_MBT-I_ by *X*_1_ at the presently studied temperatures.

e The global rate coefficient for the MBT + OH radical reaction was calculated using eq 1.

The data from Table 2 indicate that the calculated rate coefficients for the addition reaction via TS10-R7 are ∼5–18 and 10–24 times larger than those for TS9-R6 (addition channel) and the major abstraction channel via TS1-R1, respectively, within the studied temperature range. For example, the obtained rate coefficient at 298 K for the addition reaction proceeding via TS10-R7 is 6.0 × 10^–11^ cm^3^ molecule^–1^ s^–1^, which is 7 and 11 times larger compared to the rate coefficients for TS9-R6 and TS1-R1, with rate coefficients of 9.2 × 10^–12^ and 5.3 × 10^–12^ cm^3^ molecule^–1^ s^–1^, respectively. These data indicate that OH radical addition via TS10-R7 is more dominant compared to the other possible channels studied in this work.

We also calculated rate coefficients for all the abstraction and addition pathways associated with the second conformer of MBT (see Figure 1) and its interaction with the OH radical, along with their corresponding PES profiles, using the MESMER kinetic code. The site-specific H atom abstraction and addition path rate coefficients for the MBT-II + OH radical reaction are provided in Table S7 of the Supporting Information. We observed that the rate coefficient trend for all the reaction paths associated with the MBT-II + OH radical reaction was similar to those observed for the MBT-I + OH radical reaction.

Additionally, we determined the total rate coefficient for the MBT-I + OH radical (*k*_MBT-I_) and MBT-II + OH radical (*k*_MBT-II_) reactions by adding the corresponding individual reaction pathway rate coefficients at their respective temperatures. The reactions were similar and of similar energies. The rate coefficients obtained for the MBT-II + OH radical reaction were used for the MBT-III + OH radical reaction.

Within the two methyl groups of MBT-I, a total of six hydrogen atoms are present. The molecular structure reveals that for each of the two methyl groups, the attached hydrogen atoms are not entirely equivalent. This disparity primarily arises from the differences in the orientation of the methyl group hydrogen atoms in relation to the −SH group. While there are six hydrogen atoms involved in total, each methyl group consists of two equivalent H atoms. The corresponding transition states are designated as TS5-R4 and TS8-R5 (refer to Figure 7). Additionally, one H atom differs in each methyl group, identified as TS6-R4 and TS7-R5 (see Figure 7). Consequently, there are four transition states arising from the two methyl groups. This implies that the OH radical can abstract hydrogen from the two methyl groups through four distinct paths. The two equivalent H atoms from each methyl group contribute to one transition state, and the degeneracy of the reaction path for TS5-R4 and TS8-R5 is assumed to be 2. As a result, the rate coefficients for channels TS5-R4 and TS8-R5 were multiplied by a factor of 2 in calculating the total rate coefficient of the MBT-I + OH radical reaction.

Finally, we calculated the global rate coefficient for the title reaction (MBT + OH radical) using eq 1
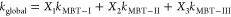
1

In eq 1, *X*_1_, *X*_2_, and *X*_3_ are the mole fractions of conformers MBT-I, MBT-II, and MBT-III, respectively. The terms *k*_MBT-I_, *k*_MBT-II_, and *k*_MBT-III_ denote the total rate coefficients obtained for the reactions of MBT-I + OH radical, MBT-II + OH radical, and MBT-III + OH radical.

The mole fractions (*X_i_*; *i* = 1, 2, and 3) of each conformer were calculated using eqs 2–4.
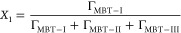
2
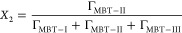
3
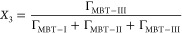
4

In eqs 2–4, the terms Γ_MBT-I_, Γ_MBT-II_, and Γ_MBT-III_ represent the Boltzmann weight factors for the MBT-I, MBT-II, and MBT-III conformers, respectively. The Boltzmann weight factors for MBT-I, MBT-II, and MBT-III were calculated using equations Γ_MBT-I_ = exp(−Δ*G*_1_/*RT*), Γ_MBT-II_ = exp(−Δ*G*_2_/*RT*), and Γ_MBT-III_ = exp(−Δ*G*_3_/*RT*), respectively. In these equations, Δ*G*_1_, Δ*G*_2_, and Δ*G*_3_ are the Gibbs free energy differences between the respective conformer and the most stable conformer obtained at the CCSD(T)//M06-2X level. The symbols *R* and *T* are the gas constant and temperature, respectively.

The obtained weight factors for MBT-I, MBT-II and MBT-III and the sum of the weight factors (Γ_MBT-I_ + Γ_MBT-II_ + Γ_MBT-III_) for these conformers in the temperature range between 200 and 320 K are given in Table S8. The mole fraction of each conformer of MBT was calculated using eqs 2–4, and the obtained values in the temperature range between 200 and 320 K are given in Table 2 and Tables S7 and S8.

**Figure 8:**
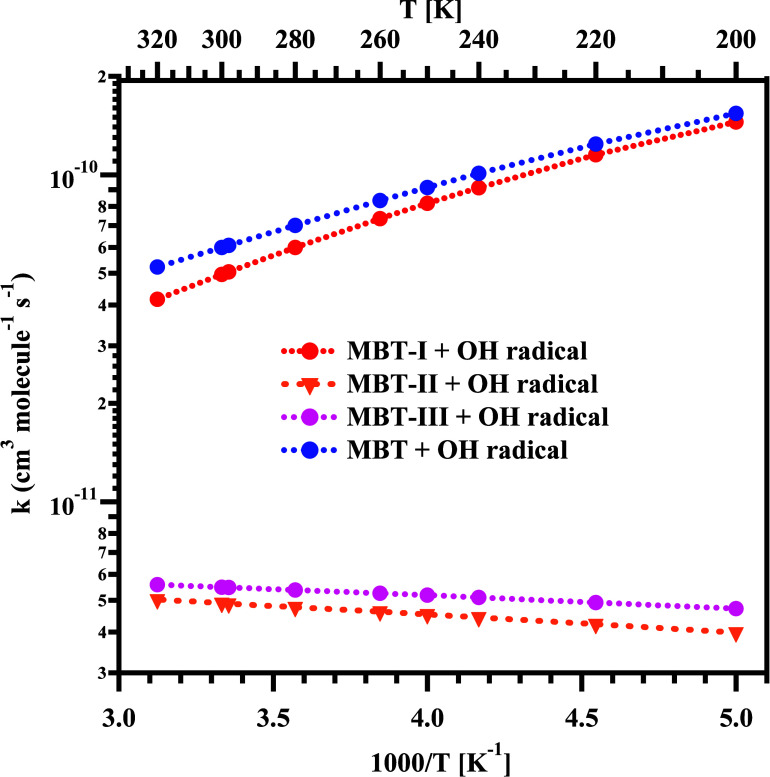
Comparison between the total rate coefficients estimated for the MBT-I + OH radical (*k*_1_), MBT-II + OH radical (*k*_2_), and MBT-III + OH radical (*k*_3_) reactions, with computed global rate coefficients (*k*_global_) for the MBT + OH radical reaction in the temperature range of 200–320 K and at 1 atm.

The total rate coefficients for the MBT-I + OH radical (*k*_1_ = *X*_1_*k*_MBT-I_), MBT-II + OH radical (*k*_2_ = *X*_2_*k*_MBT-II_), and MBT-III + OH radical (*k*_3_ = *X*_3_*k*_MBT-III_) reactions were calculated by multiplying the mole fraction of the corresponding conformer and the sum of the individual site-specific reactions within the studied temperature range. The obtained values are displayed in Table 2 and Tables S7 and S8, respectively, and plotted in Figure 8 between 200 and 320 K. The data in Table 2 and Figure 8 reveal a negative temperature dependence trend (total rate coefficient values decrease with temperature) for the MBT-I + OH radical reaction within the studied temperature range. Also, the total rate coefficients obtained for the MBT-I + OH reaction were found to be ∼1–2 orders of magnitude larger than the total rate coefficient obtained for the MBT-II + OH radical and MBT-III + OH radical reactions (see Figure 8). For example, at 298 K, the total rate coefficient (*k*_1_) for the MBT-I + OH radical reaction was found to be 5.0 × 10^–11^ cm^3^ molecule^–1^ s^–1^, whereas the total rate coefficients for the MBT-II + OH radical (*k*_2_) and MBT-III + OH radical (*k*_3_) reactions were found to be 4.9 × 10^–12^ and 5.5 × 10^–12^ cm^3^ molecule^–1^ s^–1^, respectively. We observed a slightly positive temperature dependence for the total rate coefficients obtained for the MBT-II + OH radical and MBT-III + OH radical reactions in the presently studied temperature range (see Figure 8 and Tables S7 and S8). This trend is primarily due to the increase in the populations of the less stable conformers (i.e., MBT-II and MBT-III) with increasing temperature (see Tables S7 and S8). Thus, the obtained total rate coefficients for the MBT-II + OH radical and MBT-III + OH radical reactions exhibit a slightly positive temperature dependence in the studied temperature range.

Finally, the global rate coefficient (*k*_global_) for the MBT + OH radical reaction (as a function of temperature), were calculated using eq 1. The corresponding values are displayed in Table 2, and they are also plotted in Figure 8. The calculated global rate coefficient for the MBT + OH radical reaction was also found to decrease with increasing temperature within the presently studied temperature range. For instance, at 200 and 298 K, the global rate coefficients for the MBT + OH radical reaction were estimated to be 1.5 × 10^–10^ and 6.1 × 10^–11^ cm^3^ molecule^–1^ s^–1^, respectively. Overall, the dominant channels via TS1-R1, TS9-R6, and TS10-R7 were found to significantly contribute to the global rate coefficients. This is mainly due to the lower transition state and Gibbs free energy barriers for these paths (illustrated in Figures 3–5 and Table 1) compared to other possible channels.

To compare the global rate coefficients obtained by considering the contributions from the three conformers of MBT in their reactions with the OH radical to those obtained by considering the most stable conformer of MBT with the OH radical reaction using hindered rotor treatment, we conducted hindered rotor (HR) calculations. This allowed us to evaluate the discrepancy arising from approximating anharmonic low-frequency torsions as harmonic oscillators in the computation of rate coefficients. In our calculations, we utilized the HR model developed by Chuang and Truhlar,^45^ as detailed in the Supporting Information for the computation of partition functions pertaining to the lower vibrational modes (refer to Table S9). The obtained rate coefficients for the various possible H-abstraction and OH addition paths involved in the MBT + OH radical reaction using the HR model in the temperature range of 200–320 K are given in Table S10. The application of HR treatment resulted in an approximately 4–6-fold alteration in the rate coefficients for TS1-R1 and 2-fold alteration in the rate coefficients for TS9-R6 and TS10-R7. In all other scenarios, there were no significant variations in the rate coefficients (see Table 2 and Table S10). Due to the HR treatment, the variation in the global rate coefficient for the MBT + OH reaction was found to be approximately 2- to 3-fold in the temperature range between 200 and 320 K (see Table 2 and Table S10). For example, at 298 K, the global rate coefficient calculated with hindered rotor considerations was found to be 1.7 × 10^–10^ cm^3^ molecule^–1^ s^–1^, whereas consideration of the contributions from the reaction of the three conformers with the OH radical yielded a global rate coefficient of 6.1 × 10^–11^ cm^3^ molecule^–1^ s^–1^ at the same temperature (see Table 2 and Table S10).

The current CCSD(T)//M06-2X calculations yield energies with an accuracy within 1 kcal mol^–1^.^46,47^ To get the uncertainty in the computed rate coefficients, we performed rate coefficient calculations by varying the barrier heights within the uncertainty of the CCSD(T)//M06-2X energies for all possible H-abstraction and OH addition paths for the MBT + OH radical reaction. The calculated rate coefficients for all the paths and the global rate coefficient for the MBT + OH radical are provided in Table S11 of the Supporting Information. The global rate coefficient data in Tables S10 and S11 clearly suggest that the introduction of 1 kcal mol^–1^ uncertainty into the CCSD(T)//M06-2X barrier heights results in a factor of ∼2 uncertainty in the predicted global rate coefficients in the temperature range between 200 and 320 K.

### 4.1 Atmospheric Implications

The experimentally determined rate coefficient for the reaction between MBT and the OH radical has not been reported. Therefore, our comparison relies on existing literature regarding the reactions of thiols as a class with the OH radical. In our study, the rate coefficient for the MBT + OH radical reaction was estimated to be 6.1 × 10^–11^ cm^3^ molecule^–1^ s^–1^ at 298 K. This value is approximately twice as large as the rate coefficients for the CH_3_SH + OH radical^48^ (*k* = 3.3 × 10^–11^ cm^3^ molecule^–1^ s^–1^) and (CH_3_)_2_CHSH + OH radical^22^ (*k* = 3.9 × 10^–11^ cm^3^ molecule^–1^ s^–1^) reactions. However, it aligns well with the rate coefficients of the CH_3_CH_2_SH + OH radical^22^ (*k* = 4.5 × 10^–11^ cm^3^ molecule^–1^ s^–1^) and CH_3_CH_2_CH_2_SH + OH radical^22^ (*k* = 5.3 × 10^–11^ cm^3^ molecule^–1^ s^–1^) reactions at the same temperature.

The larger rate coefficients observed for the MBT + OH radical reaction at 298 K, in comparison to the values of the CH_3_SH + OH radical and (CH_3_)_2_CHSH + OH radical reactions at the same temperature, can be attributed primarily to the presence of additional reaction channels for MBT that significantly contribute to the overall reaction. These channels involve OH addition and hydrogen abstractions from the SH and alkyl groups. In contrast, the CH_3_SH + OH radical and (CH_3_)_2_CHSH + OH radical reactions lack addition paths, and their reaction paths are limited mainly to hydrogen abstractions from the SH and alkyl groups. Furthermore, the increase in alkyl chain length in the reactions of the CH_3_CH_2_SH + OH radical, and CH_3_CH_2_CH_2_SH + OH radical results in a proportional increase in the contribution from the H-abstraction paths to the overall reaction. This phenomenon aligns their reaction rate coefficients more closely with the rate coefficient of the MBT + OH radical reaction at the same temperature.

We compared the current reaction rate coefficient of the MBT + OH radical at 298 K with the reactions involving Cl atom + thiols. Our findings indicate that the rate coefficient for the MBT + OH radical reaction at 298 K is approximately 3 to 4 times smaller than the rate coefficients observed for CH_3_SH + Cl atom^48^ (*k* = 2.0 × 10^–10^ cm^3^ molecule^–1^ s^–1^), CH_3_CH_2_SH + Cl atom^49^ (*k* = 1.9 × 10^–10^ cm^3^ molecule^–1^ s^–1^), CH_3_CH_2_CH_2_SH + Cl atom^49^ (*k* = 2.6 × 10^–10^ cm^3^ molecule^–1^ s^–1^), and (CH_3_)_2_CHSH + Cl atom^49^ reactions (*k* = 2.7 × 10^–10^ cm^3^ molecule^–1^ s^–1^) at the same temperature. Considering the previously reported rate coefficients for thiol + Cl atom reactions, we anticipate that the rate coefficient for the reaction between the title molecule (MBT) and the Cl atom will also fall within the order of 10^–10^ cm^3^ molecule^–1^ s^–1^ under room temperature conditions. Consequently, it can be concluded that under tropospheric conditions, MBT would mainly be scavenged by the OH radical and Cl atoms.

The primary mechanism for the atmospheric removal of MBT is through reactions with the OH radical, which plays a key role in determining its atmospheric lifetime. The lifetime of MBT with the OH radical in the atmosphere was calculated using the formula^50,51^ given in eq 5
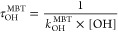
5where τ_OH_^MBT^ and *k*_OH_^MBT^ are the atmospheric lifetime and the rate coefficient (in cm^3^ molecule^–1^ s^–1^) for the oxidation of MBT initiated by OH radicals, respectively. In eq 5, [OH] represents the average concentration of the OH radical under atmospheric conditions, which is 1.0 × 10^6^ molecules cm^–3^.^52^ Thus, the lifetime of MBT at 298 K calculated using eq 5 was found to be 5 h. We also estimated the lifetime of MBT with respect to its reaction with the OH radical in the entire studied temperature range of 200 and 320 K and found it to vary between 2.0 and 5.0 h. This estimated atmospheric lifespan indicates that MBT is a highly short-lived compound with limited atmospheric mixing. The atmospheric persistence of MBT is contingent on the timing and emission location and would be influenced by fluctuations in OH radical concentrations. If other active processes, such as photolysis or deposition come into play, then it could lead to an even more reduced atmospheric lifespan.

At the moment, there is a lack in the existing literature of kinetic data concerning the interaction of MBT with Cl atoms or NO_3_ radicals. It is anticipated that the rate coefficient for the reaction with Cl atoms could be 1 order of magnitude larger than that with OH radicals. This suggests that in regions with elevated Cl atom concentrations, the gas-phase Cl atom + MBT reaction might play a role in the atmospheric removal of MBT. The increased reactivity of Cl atoms would, to some extent, offset the lower expected atmospheric abundance of Cl atoms compared to OH radicals. Consequently, Cl chemistry might act as a loss mechanism for MBT in areas with higher pollution levels and coastal regions, where elevated Cl levels and MBT emissions are expected. Based on the limited available kinetic data for the NO_3_ radical reaction with MBT (e.g., the rate coefficient for the NO_3_ + CH_3_SH reaction is approximately 10^–12^ cm^3^ molecule^–1^ s^–1^), it is likely that the NO_3_ radical reaction may also significantly influence the atmospheric lifetime of MBT.

The results of the OH radical-initiated degradation of MBT confirm that the primary degradation mechanisms involve addition reactions to the double bonds within the molecule. These addition pathways lead to the formation of C-centered MBT–OH radical products. Once released into the atmosphere, these products have the potential to undergo autoxidation to form other compounds or react with atmospheric oxygen (O_2_) to generate corresponding peroxy radicals. These peroxy radicals are of particular significance as they can serve as crucial precursors for the production of OH radicals, S atom-containing compounds, and highly oxygenated organic molecules (HOMs) within the atmosphere. This study offers valuable insights into the significant role of multifunctional VOSCs in atmospheric chemistry.

## 5 Conclusions

We studied the energetics and kinetics of the atmospheric oxidation of MBT mediated by the OH radical using high-level quantum chemistry calculations. H-abstraction and OH radical addition paths were identified. The reaction of MBT + OH radical via addition of the OH radical to a C atom of the double bond leading to a C-centered MBT–OH radical intermediate was found to be major. The results from thermochemical assessments revealed that all possible abstraction and addition paths are highly exothermic and spontaneous. The rate coefficient calculations for all H-abstraction and addition paths were estimated using MESMER in the temperature range of 200 to 320 K and at 1 atm pressure. The global rate coefficient for the MBT + OH radical reaction at 298 K was estimated to be 6.1 × 10^–11^ cm^3^ molecule^–1^ s^–1^. The lifetime of MBT with respect to its reactions with the OH radical was found to be 2–5 h in the atmospherically relevant temperature range of 200 to 320 K. This suggests that this molecule is short-lived in the atmosphere and its direct contribution to global warming is almost negligible. However, the possibility exists that the further interaction of the initial products of the MBT + OH radical reaction with other atmospheric radicals may lead to compounds with longer lifetimes and significant environmental impacts.

## Supporting Information Available

Tables S1–S12: optimized geometries of all the stationary points at the M06-2X level of theory, vibrational frequencies, rotational constants, and imaginary frequencies of various transition states obtained at the M06-2X level, calculated total electronic energies including zero-point energy corrections calculated at various levels of theory, T1 diagnostic values for all the stationary points, rate coefficients for all possible paths for the MBT-II + OH radical reaction, weight factors and mole fractions of MBT-III, lower vibrational modes for various stationary points treated as hindered rotors, rate coefficients for the MBT + OH radical reaction with the hindered rotor (HR) treatment, rate coefficients for the H-abstraction and addition paths by introducing the uncertainty of 1 kcal mol^–1^ into the CCSD(T) barriers for the MBT + OH radical reaction, and MESMER input files; Figure S1: optimized geometries of the postreactive complexes and products; Figures S2–S4: PES diagrams for the various abstraction and addition paths associated with the MBT-II + OH radical reaction (PDF)
