# Supplementary material for: Theoretical Insights into the Gas-Phase Oxidation of 3-Methyl-2-butene-1-thiol by the OH Radical: Thermochemical and Kinetic Analysis
Source: J Phys Chem A. 2024 Mar 11;128(11):2136–49. doi: 10.1021/acs.jpca.3c07775 (PMC10961829; doi:10.1021/acs.jpca.3c07775)
Supplement: Supplementary file 1 — jp3c07775_si_001.pdf [file jp3c07775_si_001.pdf]

Theoretical Insights into the Gas-Phase Oxidation of 3-Methyl-2-Butene-1-Thiol by the OH Radical: Thermochemical and Kinetic Analysis

Parandaman Arathala, and Rabi A. Musah*

University at Albany—State University of New York, Department of Chemistry, 1400 Washington Avenue, Albany, NY 12222, USA

*Address correspondence to: rmusah@albany.edu

SUPPORTING INFORMATION

Tables S1-S12 present the optimized geometries of all the stationary points computed at the M06-2X level of theory; vibrational frequencies, rotational constants, and imaginary frequencies of various transition states obtained at the M06-2X level; calculated total electronic energies including zero-point energy corrections calculated at various levels of theory; T1 diagnostic values for all the stationary points; rate coefficients for all possible paths for the MBT-II + OH radical reaction; weight factors and mole fractions of MBT-III; lower vibrational modes for various stationary points treated as hindered rotors; rate coefficients for the MBT + OH radical reaction with the hindered rotor (HR) treatment; rate coeffiicents for the H-abstraction and addition paths following the introduction of the uncertainity of 1 kcal mol^{-1} in the CCSD(T) barriers for the MBT + OH radical reaction; and MESMER input files. Figure S1 presents optimized geometries of the post-reactive complexes and products; Figures S2-S4 present the PES diagrams for the various abstraction and addition paths associated with the MBT-II + OH radical reaction.

Table S1: The M06-2X/aug-cc-pV(T+d)Z level optimized geometries of reactants, pre-reactive complexes, transition states, post-reactive complexes, and products involved in the MBT + •OH reaction.

MBT-I

C	0.00525200	0.01894900	-0.02394800
H	1.09119800	0.04786600	-0.04039000
H	-0.30382000	-0.58208600	0.83403200
H	-0.36267700	1.03162200	0.15636700
C	-0.60055700	-0.53942000	-1.27920200
C	-2.09316000	-0.69539300	-1.22636100
H	-2.37968900	-1.36716500	-0.41384500
H	-2.57164900	0.26555900	-1.02348800
H	-2.49126500	-1.09017800	-2.15875000
C	0.08539100	-0.88809600	-2.36651900
H	-0.45949200	-1.31357700	-3.20295700
C	1.56167900	-0.79872900	-2.55545500
H	1.79854100	-0.41763800	-3.54822000
H	2.04178200	-0.14729600	-1.83248900
S	2.36865100	-2.44010400	-2.51528000
H	1.87431500	-2.80601500	-1.32829600

MBT-II

C	-0.00843700	0.00059400	0.00870500
H	1.07608600	0.05803100	0.07455700
H	-0.43628100	0.82330500	0.58626300
H	-0.30324100	0.15864400	-1.03100800
C	-0.52825500	-1.31583200	0.50705900
C	-2.02129000	-1.45964400	0.46279300
H	-2.49328700	-0.72909000	1.12404800
H	-2.38110600	-1.24902200	-0.54631700
H	-2.36333200	-2.45433700	0.73578500
C	0.29864800	-2.26512000	0.94031600
H	1.36641300	-2.08112400	0.89587100
C	-0.11029900	-3.60966700	1.45582700
H	0.63653400	-3.99117100	2.14682000
H	-1.06991000	-3.57414500	1.96363400
S	-0.22650600	-4.76049800	0.03578700
H	-0.42993700	-5.88052300	0.73985900

MBT-III

C	0.05358200	0.12803100	-0.04224200
H	1.13718900	0.15093300	0.03313000
H	-0.20523800	0.13967800	-1.10322600
H	-0.30826900	-0.82076200	0.36057700
C	-0.61232900	1.27891300	0.65142500

C	-2.10591400	1.31703900	0.51336600
H	-2.39088000	1.37610400	-0.53960800
H	-2.55058200	0.40106300	0.90912700
H	-2.53884900	2.16645700	1.03792700
C	0.04417600	2.22018700	1.32591000
H	-0.52838600	3.03309200	1.76126400
C	1.52351200	2.29464700	1.52884100
H	1.76031900	2.83071800	2.44412400
H	1.97633100	1.30827000	1.60297000
S	2.42038600	3.09262600	0.14420100
H	1.65215300	4.18510300	0.08161600

TS1-R1

C	-0.04866600	-0.18197700	0.00158900
H	1.03543200	-0.23732000	0.02370300
H	-0.43060300	-0.82234600	0.79979000
H	-0.35161500	0.83868700	0.24554900
C	-0.64563800	-0.59563300	-1.31298600
C	-2.14727300	-0.65450100	-1.31862200
H	-2.50835900	-1.35759400	-0.56428700
H	-2.56559400	0.32114800	-1.06151900
H	-2.53794400	-0.95399000	-2.28880600
C	0.05298400	-0.89394300	-2.41020200
H	-0.49276700	-1.20082500	-3.29706100
C	1.53786700	-0.87110800	-2.56831000
H	1.80866200	-0.32190900	-3.47059700
H	2.04962200	-0.40373000	-1.73370500
S	2.24783600	-2.53185500	-2.84509600
H	2.05781600	-2.98721500	-1.55069700
O	0.95334200	-3.37431700	-0.54729500
H	0.19827700	-2.88527500	-0.92279200

RC1

C	-0.44489600	1.32590500	1.64171200
H	0.61394700	1.52536600	1.49432000
H	-0.53637400	0.51662300	2.37076700
H	-0.90524800	2.21118600	2.08394800
C	-1.15713100	0.93845100	0.37938400
C	-2.64507500	0.79638500	0.51604900
H	-2.88744400	0.06274300	1.28952100
H	-3.09217400	1.74158500	0.83073600
H	-3.11081200	0.48629600	-0.41736600
C	-0.53668900	0.67970000	-0.77204500
H	-1.13266500	0.35600300	-1.61940800
C	0.94090600	0.75203600	-0.99822100

H	1.16009100	0.88078600	-2.05575500
H	1.40221500	1.58064800	-0.46806100
S	1.82327100	-0.78819900	-0.54536600
H	1.60254900	-0.70425700	0.77199100
O	-0.30053800	-2.12162600	1.00865200
H	-0.74496400	-1.52129500	0.38334800

PC1

C	-0.41759000	1.41473700	1.62460900
H	0.65217100	1.57702900	1.52190500
H	-0.57318500	0.66062800	2.39843200
H	-0.88702400	2.33750400	1.97270200
C	-1.07088700	0.95108300	0.35845300
C	-2.52897800	0.62549700	0.48568200
H	-2.66558600	-0.17262900	1.21893000
H	-3.08019600	1.49479200	0.85035000
H	-2.96050200	0.31108500	-0.46238600
C	-0.43119200	0.80820300	-0.80773400
H	-0.97481400	0.41711900	-1.66000600
C	1.02597500	1.09693400	-1.04103400
H	1.20902000	1.31485600	-2.08987500
H	1.39115200	1.91596600	-0.42862400
S	1.82159700	-0.45740800	-0.58711600
H	0.21132700	-2.23538400	1.73936600
O	-0.67312200	-1.97011100	1.47581500
H	-0.53953700	-1.46715000	0.66547000

$(CH_3)_2C=CHCH_2S^{\cdot}$ (P1)

C	-0.41358200	1.40785500	1.62476500
H	0.65729500	1.55946600	1.52021100
H	-0.57015300	0.66825200	2.41250100
H	-0.87360300	2.33708200	1.96903800
C	-1.06950200	0.94268300	0.35939200
C	-2.52957300	0.62391000	0.48360000
H	-2.68386200	-0.16654700	1.22150800
H	-3.08170900	1.49743500	0.83755500
H	-2.95671600	0.30305400	-0.46406900
C	-0.43402700	0.81228400	-0.80724600
H	-0.97837300	0.42152800	-1.65882200
C	1.02271000	1.09869900	-1.04039100
H	1.20487700	1.31484500	-2.08998500
H	1.38541500	1.92294400	-0.43281800
S	1.83076400	-0.44809500	-0.58095100

TS2-R2

C	-0.03235000	0.25091000	-0.13412000
H	1.04864500	0.32839900	-0.19700700
H	-0.29226400	-0.18186800	0.83432200
H	-0.44443000	1.26249900	-0.14450100
C	-0.64750200	-0.55656500	-1.23768400
C	-2.13491200	-0.71988600	-1.12604100
H	-2.39577200	-1.20904700	-0.18472800
H	-2.62452500	0.25675900	-1.11873600
H	-2.54127000	-1.30579500	-1.94736000
C	0.02024000	-1.10202700	-2.25939400
H	-0.54046800	-1.65906000	-3.00249500
C	1.47461800	-1.03093200	-2.50690000
H	1.66761500	-0.36412000	-3.42448800
H	2.04849000	-0.57978400	-1.70459400
S	2.22596700	-2.60137200	-3.02133100
H	2.01231300	-3.25695000	-1.87501300
O	2.34124800	0.29900700	-4.58593200
H	3.16134900	-0.22188600	-4.57856900

RC2

C	-0.52541900	1.21117400	1.78281800
H	0.48558800	1.24974500	1.38453400
H	-0.48072400	0.73659000	2.76544200
H	-0.87047700	2.23446400	1.94792300
C	-1.49755500	0.47098700	0.91018300
C	-2.85677400	0.27828100	1.51746500
H	-2.78259700	-0.28620300	2.44966700
H	-3.30142800	1.24374000	1.76928000
H	-3.53039200	-0.24850300	0.84478400
C	-1.21906900	0.00069600	-0.30495500
H	-1.98602300	-0.55300200	-0.83606100
C	0.08645500	0.13190400	-1.01257600
H	-0.06248900	0.29419700	-2.07882600
H	0.68236000	0.95843800	-0.63443600
S	1.07728900	-1.41228800	-0.93750000
H	0.90181300	-1.62167500	0.37192600
O	2.88183600	1.03716700	0.24791200
H	2.80932000	0.15037300	-0.15953700

PC2

C	-0.61228500	1.46418600	1.80268500
H	0.07663100	2.05961000	1.19600300

H	-0.03352700	1.02884000	2.62092700
H	-1.34833900	2.14112900	2.23198200
C	-1.27521600	0.40246400	0.97911500
C	-2.74816200	0.51420000	0.76155200
H	-3.28230000	0.50827600	1.71595700
H	-2.99401700	1.46229200	0.27462600
H	-3.13242700	-0.29756200	0.14652200
C	-0.54980900	-0.63078100	0.42707300
H	-1.09153200	-1.36819200	-0.15835900
C	0.82481300	-0.79035600	0.54952200
H	0.19088500	1.11892200	-1.01128100
H	1.42066400	-0.08875300	1.11681800
S	1.77017500	-2.05966300	-0.15619500
H	0.76494700	-2.70541000	-0.75834700
O	0.51193900	1.93812500	-1.40413600
H	1.29907300	1.68693200	-1.89182900

$(CH_3)_2C$=CHC•HSH (P2)

C	-0.61105900	1.46615100	1.79695100
H	0.15395200	1.99850100	1.22523200
H	-0.11293800	1.03539100	2.66989000
H	-1.33396400	2.19851400	2.15180700
C	-1.27372400	0.40756500	0.96903800
C	-2.75139700	0.50475200	0.77510500
H	-3.27369200	0.48476800	1.73630600
H	-3.02139200	1.45141400	0.29762300
H	-3.13403200	-0.30889300	0.16139200
C	-0.54515800	-0.61708100	0.40940700
H	-1.08523400	-1.35237300	-0.17996200
C	0.82475700	-0.78236300	0.54073500
H	1.42140000	-0.08742100	1.11482300
S	1.76819000	-2.05985200	-0.15397900
H	0.76390600	-2.69879300	-0.76449000

TS3-R2

C	1.04665100	0.23218300	-0.64363700
H	2.12042300	0.38892200	-0.70844000
H	0.84838000	-0.57139800	0.07082600
H	0.59805900	1.13591100	-0.22628300
C	0.41070600	-0.09476000	-1.96244400
C	-1.08615400	-0.18606200	-1.93804700
H	-1.41017600	-0.93533600	-1.21215800
H	-1.51957600	0.76556800	-1.62277100

H	-1.49314300	-0.44771500	-2.91247600
C	1.09426000	-0.30662700	-3.08640200
H	0.54590600	-0.54825400	-3.99084700
C	2.57698000	-0.25786700	-3.22723100
H	2.87964500	-0.08041900	-4.25682000
H	3.00961400	0.62046800	-2.63367200
S	3.45961400	-1.75572700	-2.72274600
H	2.79290500	-1.93910600	-1.57811600
O	4.01018500	1.49756500	-1.92705000
H	4.66291800	0.77886800	-1.87750300

PC3

C	-0.93783800	-0.22790900	1.68150200
H	-0.25213000	0.53122700	2.07107100
H	-0.43192200	-1.19012100	1.70858000
H	-1.78798300	-0.26791900	2.36369500
C	-1.41692800	0.12390700	0.30509300
C	-2.64788300	0.97515600	0.25958000
H	-3.48278500	0.47585300	0.75926300
H	-2.48215600	1.91303600	0.79836800
H	-2.94604700	1.21470600	-0.75928900
C	-0.83658100	-0.29815800	-0.87165800
H	-1.39626300	-0.06196200	-1.77253900
C	0.34839700	-0.98583500	-1.13222200
H	0.51152300	-1.34514300	-2.13828600
H	0.48645400	1.60145600	-0.11711400
S	1.68885200	-1.36077300	-0.09580100
H	1.51597200	-0.38605000	0.80485200
O	1.12134000	2.03748400	0.46370000
H	1.89088300	2.20556400	-0.08498300

TS4-R3

C	-0.02670200	0.05513800	0.02128700
H	1.05697000	0.12690600	-0.01229700
H	-0.30503500	-0.58074500	0.86407100
H	-0.43135300	1.04834300	0.22556100
C	-0.62770000	-0.49176900	-1.24551200
C	-2.12144100	-0.64924700	-1.22221400
H	-2.41715000	-1.30348600	-0.39932100
H	-2.59986600	0.31695000	-1.04829600
H	-2.49341900	-1.06682900	-2.15463200
C	0.07979400	-0.80893500	-2.31892600
H	-0.53561500	-1.22928500	-3.25261800

C	1.53914500	-0.79810200	-2.58275000
H	1.75898600	-0.27998000	-3.51581400
H	2.09741400	-0.30841100	-1.78808200
S	2.20940200	-2.47816500	-2.84426900
H	1.82598500	-2.97033100	-1.66138200
O	-0.96385800	-2.01435300	-4.23371000
H	-0.27321400	-2.69350600	-4.15148700

RC3

C	-0.33419500	0.64827200	2.10594700
H	0.75340600	0.64029900	2.08918900
H	-0.67606900	-0.30757200	2.50936900
H	-0.66316400	1.42392400	2.80031600
C	-0.95281200	0.85663900	0.75613300
C	-2.44862300	0.95593700	0.75972100
H	-2.88177300	0.07346300	1.23265700
H	-2.76707500	1.82763200	1.33613500
H	-2.84760700	1.03508100	-0.24899700
C	-0.25328400	0.93604200	-0.37918500
H	-0.79835000	1.03446500	-1.31173700
C	1.23593600	0.87338000	-0.49301300
H	1.57281800	1.38571800	-1.39167100
H	1.73951000	1.33486900	0.35155900
S	1.89921000	-0.82637600	-0.68614200
H	1.54437400	-1.25532200	0.53142000
O	-1.34135900	-1.64468500	-0.16346300
H	-0.50531300	-1.69061500	-0.66323000

PC4

C	-0.31854000	1.42232100	2.37642600
H	0.76294300	1.47953800	2.27762400
H	-0.55472800	0.80743900	3.24701600
H	-0.70250800	2.42463500	2.57531700
C	-0.98323800	0.84871500	1.14742900
C	-2.48089700	0.72346500	1.22420800
H	-2.76309000	0.08883600	2.06675100
H	-2.93316900	1.70290300	1.39255600
H	-2.89491500	0.29828300	0.31302500
C	-0.30893400	0.48574500	0.08242300
H	-1.26620300	-0.30663400	-2.13148400
C	1.08200400	0.42925700	-0.37454300
H	1.17720800	0.83041700	-1.38360400

H	1.73649500	1.00821200	0.28103200
S	1.72523200	-1.27840700	-0.51089000
H	1.34212900	-1.67326800	0.70773400
O	-0.77421400	-0.81224600	-2.78464000
H	-0.20994700	-1.38670000	-2.25510300

$(CH_3)_2C{=}C^{\bullet}CH_2SH$ (P3)

C	-0.31730300	1.40347100	2.37470100
H	0.76479900	1.44831800	2.27347000
H	-0.55746000	0.78345400	3.24082700
H	-0.68775300	2.40864800	2.58561500
C	-0.99141100	0.84900100	1.14189800
C	-2.49042400	0.73961800	1.22092500
H	-2.77897700	0.09376600	2.05306100
H	-2.93216800	1.72101600	1.40675700
H	-2.90744500	0.33482200	0.30218600
C	-0.32735400	0.48408100	0.07102100
C	1.06396900	0.41895400	-0.38227300
H	1.16732400	0.81843500	-1.39104600
H	1.71981600	1.00557500	0.26606500
S	1.70715200	-1.28788900	-0.50481300
H	1.45322600	-1.62317900	0.76410900

TS5-R4

C	0.20798000	0.28619100	-0.31152000
H	0.67493500	-0.48297800	0.39524200
H	-0.44790000	0.88791400	0.31628200
H	1.02700200	0.89203400	-0.68907500
C	-0.52997000	-0.47843100	-1.35021300
C	-1.91007400	-0.92247700	-0.96354800
H	-1.87079100	-1.51520400	-0.04662100
H	-2.55058600	-0.06194800	-0.76028200
H	-2.37149600	-1.52383200	-1.74386300
C	0.01690200	-0.83255100	-2.51727600
H	-0.56306800	-1.45250700	-3.19211200
C	1.40844700	-0.52037600	-2.94977900
H	1.47528500	-0.46127000	-4.03413100
H	1.77194000	0.41932800	-2.54428400
S	2.57282800	-1.85882900	-2.49328600
H	2.22435100	-1.90862700	-1.20023900
O	1.61524600	-1.48319000	1.10590800
H	2.30884800	-0.85098200	1.35532800

PC5

C	-0.90500400	1.79619900	0.81021500
H	0.14877500	0.02925200	1.93461300
H	-1.47921700	2.24957000	1.60571900
H	0.02560100	2.27474700	0.54252400
C	-1.37717300	0.68626000	0.15770900
C	-2.68000600	0.06228900	0.60071400
H	-2.49224800	-0.89859100	1.08199800
H	-3.20577400	0.70186600	1.30621400
H	-3.33231800	-0.11889300	-0.25294700
C	-0.70142100	0.05589900	-0.89628900
H	-1.15294600	-0.82789100	-1.33032300
C	0.62128100	0.43764300	-1.43686200
H	0.59610700	0.49380800	-2.52540500
H	0.97466700	1.39296500	-1.06234800
S	1.87462200	-0.85830800	-1.09697400
H	1.66108600	-0.91853600	0.22551500
O	0.70084200	-0.70799200	2.22293900
H	1.08715200	-0.43546400	3.05786600

$(CH_3)(^{\bullet}CH_2)C{=}CHCH_2SH$ (P4)

C	-0.74101900	1.59888000	0.85657700
H	-1.21484900	2.02643100	1.72820700
H	0.23121100	1.98700400	0.58710000
C	-1.35577200	0.61067200	0.13910100
C	-2.70525900	0.09071400	0.57236500
H	-2.64321900	-0.96656300	0.83170700
H	-3.07760200	0.63328000	1.43845000
H	-3.43376500	0.18850400	-0.23256800
C	-0.77410600	0.02475900	-0.99463800
H	-1.28479600	-0.80641800	-1.46422900
C	0.55482400	0.39240500	-1.52893400
H	0.64171300	0.14204300	-2.58388700
H	0.77401600	1.45055300	-1.41338900
S	1.89977600	-0.57925900	-0.72996900
H	1.55610400	-0.26397800	0.52356900

TS6-R4

C	-0.02833600	-0.28508000	0.19355700
H	1.04617000	-0.42209100	0.26992900
H	-0.53668100	-0.82162600	0.99474900
H	-0.23197700	0.82304500	0.44642000
C	-0.61609300	-0.58563100	-1.13823900

C	-2.11294200	-0.70439700	-1.15081600
H	-2.43595700	-1.53580100	-0.52087600
H	-2.56415200	0.20035200	-0.73673700
H	-2.49745200	-0.86053300	-2.15637400
C	0.09936200	-0.71319700	-2.25989200
H	-0.42410500	-0.95470900	-3.17913900
C	1.58095900	-0.61002900	-2.38216300
H	1.85206200	-0.03093200	-3.26434400
H	2.04555500	-0.13498800	-1.52372500
S	2.36213900	-2.23692900	-2.67408900
H	1.87099200	-2.82506700	-1.57843800
O	-0.56344000	2.26452000	0.31138800
H	-0.46836100	2.27768000	-0.65508900

PC6

C	-0.56013700	-0.22434000	1.66874200
H	0.50588700	-0.07620900	1.77547700
H	-1.11787400	-0.45418500	2.56537200
H	-0.44853900	2.15393000	1.08520300
C	-1.18606200	-0.15325200	0.45403900
C	-2.67212500	-0.39952800	0.35304400
H	-3.11479700	-0.55693500	1.33410000
H	-3.17296200	0.44522900	-0.12007800
H	-2.87047200	-1.28094200	-0.25737500
C	-0.49910700	0.13168800	-0.73816700
H	-1.05618000	0.10827500	-1.66610000
C	0.95953600	0.35663400	-0.82969100
H	1.20950100	0.96297200	-1.69716600
H	1.36859700	0.85074600	0.04645300
S	1.85301300	-1.22679300	-1.11268400
H	1.36945600	-1.84637900	-0.03041100
O	0.06708200	2.93162700	0.84424700
H	-0.07543200	3.56535900	1.55010000

TS7-R5

C	-0.05029100	0.10514200	-0.01898000
H	1.03210500	0.00792800	-0.02390100
H	-0.44039800	-0.43782900	0.84462000
H	-0.30154100	1.15771900	0.12702900
C	-0.70227800	-0.42083600	-1.26444900
C	-2.18634000	-0.45905100	-1.21653400
H	-2.49951400	-1.24894300	-0.43572600
H	-2.62431800	0.46176100	-0.82885400

H	-2.64891300	-0.74101600	-2.15900400
C	-0.04756500	-0.88816300	-2.33103600
H	-0.62692200	-1.29342600	-3.15425200
C	1.43215600	-0.95995700	-2.49243300
H	1.71841500	-0.75750200	-3.52319600
H	1.96014600	-0.25403400	-1.85934700
S	2.07366300	-2.64483200	-2.17962200
H	1.59869900	-2.74174700	-0.93275300
O	-2.48343000	-2.46572700	0.42617900
H	-1.94990000	-2.99595700	-0.18821900

PC7

C	-0.19031700	1.59018800	1.31919700
H	0.35856600	0.74030000	1.72761500
H	-0.90086500	1.92350500	2.07193900
H	0.52231800	2.39601000	1.14105100
C	-0.90363800	1.20736200	0.04516200
C	-2.26424300	1.36022000	-0.04912800
H	-1.64312300	-0.81695500	1.08270300
H	-2.83993700	1.78160900	0.76244200
H	-2.79179300	1.08874300	-0.95368200
C	-0.17440100	0.66901100	-1.02116400
H	-0.71171400	0.36984000	-1.91288200
C	1.26917400	0.35894600	-0.97019300
H	1.75295700	0.53789200	-1.92899400
H	1.79304000	0.92975700	-0.20911600
S	1.54910500	-1.43623000	-0.66505100
H	0.75603300	-1.51919800	0.41304900
O	-1.20585200	-1.44275200	1.67226400
H	-1.70780400	-2.25774200	1.60386700

TS8-R5

C	-0.69192100	0.07811700	-0.09840500
H	0.31641500	0.43444300	-0.28808300
H	-0.78625700	-0.12143500	0.97105600
H	-1.39698800	0.87874200	-0.33194000
C	-1.05073900	-1.15351800	-0.87759400
C	-2.43032300	-1.65165600	-0.63784600
H	-2.65217300	-2.58826500	-1.14302400
H	-2.68073400	-1.71345300	0.42227700
H	-3.17850400	-0.86780100	-1.03397500
C	-0.24932000	-1.78030300	-1.74424900
H	-0.61902800	-2.68048200	-2.22477700

C	1.15155300	-1.41089700	-2.09145700
H	1.31290400	-1.48996400	-3.16613100
H	1.41036700	-0.40033100	-1.79328200
S	2.36687500	-2.57940200	-1.38326600
H	1.98777700	-2.41877900	-0.11113400
O	-3.80708900	0.24192000	-1.80325200
H	-3.26340600	0.11343400	-2.59737300

PC8

C	-0.58560500	1.10974500	1.54857700
H	0.47931000	1.12711300	1.78546600
H	-1.14135500	1.21921300	2.47684900
H	-0.79498500	1.97379300	0.91658200
C	-0.96509900	-0.17322600	0.84852500
C	-1.97207300	-0.95830900	1.35588800
H	-2.25593600	-1.88055700	0.86770900
H	-2.50209400	-0.68133700	2.25531400
H	-1.89925600	1.09739900	-1.14503300
C	-0.29564100	-0.54854300	-0.31963200
H	-0.55208700	-1.49641400	-0.77778000
C	0.81922200	0.21329700	-0.92300300
H	0.78509800	0.17671300	-2.01060300
H	0.82107900	1.25756900	-0.62600300
S	2.44897000	-0.53843100	-0.51151800
H	2.29374000	-0.49322800	0.81593000
O	-1.75471200	1.95714700	-1.55437000
H	-2.61944600	2.25923200	-1.83961100

TS9-R6

C	-0.00495700	-0.03018600	-0.00590400
H	1.07949800	0.02588500	-0.05090900
H	-0.27699800	-0.66362800	0.84143400
H	-0.39742200	0.96523200	0.21837000
C	-0.63242300	-0.56631500	-1.25708000
C	-2.11922100	-0.72725700	-1.20059600
H	-2.39980800	-1.41981200	-0.40333900
H	-2.59119600	0.23149100	-0.97546900
H	-2.51868300	-1.09072500	-2.14407800
C	0.05006100	-0.87671000	-2.37616000
H	-0.49824500	-1.31487200	-3.20072100
C	1.52550700	-0.79705400	-2.56481100
H	1.74870700	-0.49246100	-3.58516100
H	2.00050700	-0.08339400	-1.89800900

S	2.33031200	-2.42647500	-2.38443200
H	1.90856100	-2.65884000	-1.13665300
O	-0.54190200	1.19519500	-3.17534700
H	-0.11129100	1.69831700	-2.46769900

$(CH_3)_2C(OH)=C^{\bullet}HCH_2SH$ (P5)

C	-0.33987400	0.74788100	1.91680400
H	0.73826000	0.58154100	1.92349800
H	-0.75487200	0.30320300	2.82160300
H	-0.49879800	1.83221900	2.00796500
C	-1.00449700	0.18951900	0.70129800
C	-2.47330700	-0.05218800	0.75504200
H	-2.76637800	-0.51740200	1.69705400
H	-3.03136300	0.88982100	0.67352700
H	-2.80265400	-0.68883000	-0.06623200
C	-0.39407300	0.41446400	-0.64760700
H	-0.87981700	-0.24254400	-1.37019000
C	1.10856600	0.16851700	-0.71263100
H	1.44812500	0.35849200	-1.72902500
H	1.64514900	0.85268100	-0.05509400
S	1.58364100	-1.54360400	-0.35899700
H	1.01689200	-1.61787100	0.85040000
O	-0.67283500	1.74020000	-1.12435800
H	-0.32736500	2.37292600	-0.48766300

TS10-R7

C	0.04439100	-0.11488500	0.00910400
H	1.10878400	0.07672800	-0.08929000
H	-0.09812600	-0.92417400	0.72976500
H	-0.42945000	0.77822000	0.41425900
C	-0.60955500	-0.48546400	-1.28493000
C	-2.09758700	-0.64427200	-1.21635000
H	-2.35759300	-1.43323900	-0.50672000
H	-2.55134600	0.28034200	-0.85916000
H	-2.52425300	-0.89721400	-2.18532400
C	0.07123500	-0.83299600	-2.40384100
H	-0.49617100	-1.20178000	-3.25207400
C	1.54611900	-0.78097600	-2.57562200
H	1.80102000	-0.51447700	-3.59946500
H	2.01225500	-0.05281000	-1.92104900
S	2.31423700	-2.42328500	-2.33198700
H	1.88603100	-2.59290700	-1.07621600
O	-0.39049700	1.52534300	-2.17415400

H	-0.95539500	1.44466500	-2.95633800

$(CH_3)_2C^{\bullet}CH(OH)CH_2SH$ (P6)

C	-0.40506800	0.51691100	1.73163900
H	0.65198500	0.77565800	1.66801200
H	-0.50411300	-0.50285400	2.10260300
H	-0.88014100	1.20103200	2.43459200
C	-1.06652500	0.65618400	0.36260700
C	-2.55733700	0.35562700	0.45363600
H	-2.72379400	-0.67278700	0.77508800
H	-3.02741200	1.03080100	1.16702400
H	-3.03262500	0.48886700	-0.52005400
C	-0.41610900	-0.22463400	-0.66262400
H	-0.95331800	-1.08458900	-1.04014600
C	1.01429200	-0.08605800	-0.99034200
H	1.20100600	-0.17532200	-2.06057600
H	1.41070900	0.86908800	-0.65404000
S	2.03850400	-1.45286000	-0.28848100
H	1.71198300	-1.24443400	0.99028300
O	-0.86745600	2.03331200	0.01089100
H	-1.24111500	2.17611400	-0.86414300

TS11-R1

C	-0.09651400	0.00756600	0.02092000
H	0.97610100	0.17878500	0.08518100
H	-0.60826300	0.78279700	0.59553100
H	-0.40725500	0.12957900	-1.01908200
C	-0.47582600	-1.35343900	0.52451200
C	-1.94212500	-1.66566800	0.46875300
H	-2.50537100	-0.93013300	1.04770200
H	-2.29325100	-1.58724800	-0.56291300
H	-2.18592000	-2.66180200	0.83095100
C	0.44348200	-2.20744400	0.97001100
H	1.48543600	-1.90895500	0.94689800
C	0.15625500	-3.58000200	1.49525100
H	0.97398000	-3.93868700	2.11479100
H	-0.76212300	-3.61947300	2.07506300
S	-0.07321600	-4.77560600	0.12792600
H	-0.81547800	-5.69987900	0.84312300
O	-2.32136200	-5.38419200	1.12123600
H	-2.75561600	-5.41125400	0.25591300

RC4

C	0.45762200	2.98090600	-0.70260700
H	1.53798700	3.07769400	-0.78827900
H	0.07583900	3.82695300	-0.12669200
H	0.01925200	3.05649400	-1.69990800
C	0.05746800	1.68789000	-0.05387500
C	-1.42010400	1.51132000	0.13800300
H	-1.78726500	2.22363300	0.88116100
H	-1.93701300	1.74199100	-0.79574500
H	-1.69776100	0.50204800	0.42880500
C	0.97956900	0.79933400	0.31431000
H	2.02160500	1.01951900	0.10816700
C	0.71859700	-0.50688700	0.99201900
H	1.50259800	-0.72914800	1.71283600
H	-0.25156800	-0.55200900	1.47615100
S	0.73333800	-1.83846400	-0.25133200
H	0.68902200	-2.87996300	0.58439300
O	-1.49650400	-1.91468600	-0.05744900
H	-1.72270400	-2.09628000	-0.97851400

PC11

C	0.48383100	3.03190300	-0.59242700
H	1.54993800	3.23558600	-0.51880500
H	-0.05895800	3.80461300	-0.04352200
H	0.18274700	3.11957900	-1.63882300
C	0.13317500	1.67282000	-0.06365000
C	-1.32326500	1.32710400	-0.12767800
H	-1.90241500	2.05435500	0.44644900
H	-1.67001000	1.40057300	-1.16099600
H	-1.55668600	0.32984100	0.23531400
C	1.08096400	0.86782900	0.42583300
H	2.11346200	1.19516000	0.38863500
C	0.86151300	-0.51317400	0.97196600
H	1.62638100	-0.76433800	1.70249500
H	-0.12422200	-0.65291100	1.40600200
S	1.06820000	-1.54900200	-0.49496200
H	-1.25810700	-2.10735500	-0.07548600
O	-2.01754900	-2.05887400	0.52129800
H	-2.66236300	-2.67902800	0.17610300

RC5

C	0.57819000	2.67993200	-1.08656300
H	1.58432800	2.58359200	-1.48899100

H	0.50651600	3.62711100	-0.54757300
H	-0.12669400	2.73686300	-1.91880100
C	0.22332000	1.53454200	-0.18263100
C	-1.16419000	1.60693200	0.38253400
H	-1.31004200	2.56646300	0.88335800
H	-1.89700400	1.55621300	-0.42566100
H	-1.39653400	0.81507500	1.08907900
C	1.10556000	0.56549100	0.05838100
H	2.07566800	0.62509300	-0.42500900
C	0.90297600	-0.64500100	0.90925500
H	1.73751600	-0.77989300	1.59417400
H	-0.02178700	-0.60687200	1.47650200
S	0.85598600	-2.10417000	-0.20407400
H	0.75986800	-3.04365800	0.74469600
O	-2.33018700	-1.11744000	-0.17752500
H	-1.45498500	-1.50628600	-0.38890900

TS12-R2

C	-0.16747800	0.20965500	0.01983600
H	0.91057000	0.27493000	0.15076900
H	-0.63937500	1.00455800	0.60144900
H	-0.40423200	0.40355800	-1.02862200
C	-0.70320900	-1.12773000	0.43615000
C	-2.18113200	-1.30169800	0.25539200
H	-2.71341700	-0.47331500	0.72701600
H	-2.42945500	-1.27403600	-0.80818200
H	-2.55522400	-2.23145600	0.67352300
C	0.10500500	-2.06852600	0.92475300
H	1.16219800	-1.84455900	1.01689500
C	-0.30031100	-3.43750800	1.35650500
H	0.45997900	-3.90296700	1.97684700
H	-1.24098900	-3.39853500	2.00269200
S	-0.64101700	-4.49662000	-0.07432600
H	-0.69401900	-5.65726000	0.59144300
O	-2.54302100	-3.82760500	2.67535700
H	-2.80251500	-4.37135000	1.91248400

PC12

C	0.48296100	2.84155000	-0.04195200
H	1.43825500	2.98061000	0.46041900
H	-0.30044100	3.29172300	0.57477100
H	0.49837000	3.40524000	-0.97921200
C	0.18391700	1.39904300	-0.29864200

C	-1.12422000	1.10143500	-0.96890600
H	-1.57772500	2.01939100	-1.33988800
H	-1.00293200	0.42261700	-1.81412400
H	-1.83236900	0.64315800	-0.27270600
C	1.08880200	0.42792600	0.07986400
H	2.01377300	0.78758300	0.51947300
C	1.01789200	-0.95627700	0.00175800
H	1.86024100	-1.53647100	0.34371300
H	-0.63143100	0.20898600	1.93348300
S	-0.33062600	-1.89704900	-0.58535500
H	0.19572600	-3.08811700	-0.28920800
O	-1.45164700	-0.19456400	2.23464400
H	-1.50178200	-1.01173000	1.72984800

RC6

C	-0.37110600	2.84671700	-0.35613400
H	0.68918900	3.03155500	-0.19833800
H	-0.94282500	3.58740700	0.20764000
H	-0.60013200	3.00817600	-1.41167500
C	-0.76163000	1.45891900	0.05827400
C	-2.21420900	1.13066600	-0.12473900
H	-2.83168100	1.79376200	0.48551100
H	-2.50220700	1.30142800	-1.16404300
H	-2.45585600	0.10013300	0.12230500
C	0.13870100	0.60642700	0.54444200
H	1.17394500	0.92497300	0.61503300
C	-0.14193500	-0.79634500	0.98756900
H	0.62237600	-1.12938900	1.68410200
H	-1.11802900	-0.89335800	1.45420400
S	-0.08559300	-1.89316000	-0.48414900
H	-0.05538400	-3.06307600	0.16612100
O	2.92621200	-0.64000700	0.04089200
H	2.26558700	-1.21883000	-0.39159600

TS13-R3

C	-0.00460100	0.02813700	-0.00552700
H	1.08198700	-0.00900200	-0.01772600
H	-0.32625100	0.86258800	0.62128300
H	-0.36274800	0.23250000	-1.01653900
C	-0.59516500	-1.25670700	0.49978800
C	-2.09872100	-1.30392700	0.52115000
H	-2.48682200	-0.51655500	1.17024700
H	-2.48370500	-1.10980600	-0.48172100

H	-2.49242800	-2.25848800	0.85911700
C	0.17419400	-2.25719200	0.89948300
H	1.35332600	-2.08786000	0.81784500
C	-0.14818200	-3.62229100	1.38529800
H	0.47907900	-3.87503900	2.23713000
H	-1.19311800	-3.71163000	1.67318700
S	0.22213700	-4.80974600	0.03946000
H	0.38586300	-5.89111400	0.80889400
O	2.63084500	-2.26073300	0.49187700
H	2.52273900	-3.09662200	0.00781500

PC13

C	-0.36724300	2.85332700	-0.39661400
H	0.71160400	2.94397100	-0.29558900
H	-0.84329900	3.63377800	0.20038500
H	-0.63944500	3.03257800	-1.43855000
C	-0.85193800	1.49864900	0.03990300
C	-2.34121700	1.27036700	-0.05965700
H	-2.87470600	2.00319100	0.54870700
H	-2.66682700	1.40624500	-1.09258200
H	-2.62795200	0.27067700	0.25935700
C	-0.03680800	0.56900600	0.47681000
H	2.52223600	0.36769600	0.23706200
C	-0.12413500	-0.81446700	0.96329700
H	0.70818100	-1.03798500	1.62799900
H	-1.06201600	-0.97733900	1.49814300
S	-0.03358300	-1.95182600	-0.47229900
H	0.15882200	-3.07637200	0.22703800
O	2.98766800	-0.47333800	0.20813900
H	2.41070600	-1.04144300	-0.31502800

RC7

C	0.42789400	2.62066600	0.39679900
H	1.51510400	2.62252600	0.36149100
H	0.09958200	3.30094000	1.18575000
H	0.04871600	3.01052900	-0.54926400
C	-0.12107300	1.24425400	0.62974100
C	-1.61675700	1.18736500	0.71958800
H	-1.95626800	1.73976200	1.59883300
H	-2.04763500	1.67239000	-0.15666100
H	-2.01408800	0.17800700	0.77425300
C	0.70084200	0.19803200	0.75382100
H	1.76835800	0.37749300	0.66985200

C	0.31631000	-1.22724600	0.97928000
H	0.81686100	-1.62666900	1.85959100
H	-0.75453900	-1.35226600	1.09356600
S	0.87458600	-2.20059500	-0.47352100
H	0.26273200	-3.34814900	-0.16130400
O	-0.16159500	0.64347900	-1.95319400
H	-0.04612400	-0.32099900	-1.86397700

TS14-R4

C	0.05333300	0.28389600	0.31587000
H	1.14113400	0.28694800	0.32146100
H	-0.30402200	1.01943800	1.03900800
H	-0.29232600	0.60750200	-0.66836500
C	-0.50141300	-1.07465900	0.62575900
C	-1.97983200	-1.18532500	0.56910100
H	-2.48583600	-0.26861200	0.86901400
H	-2.28364800	-1.31672100	-0.54737200
H	-2.40088600	-2.04158700	1.08765400
C	0.30047500	-2.11664800	0.86367400
H	1.37315200	-1.96324900	0.83587900
C	-0.15931900	-3.51986600	1.09759100
H	0.58038400	-4.08138300	1.66119300
H	-1.10837000	-3.55688400	1.62431400
S	-0.36946100	-4.32582300	-0.53875600
H	-0.88238100	-5.48226400	-0.10225900
O	-2.46085500	-1.86007000	-1.87165900
H	-2.00156300	-2.69856900	-1.67887100

PC14

C	0.99086500	2.75442500	0.51435800
H	1.76136400	2.84214200	1.28067900
H	0.35146000	3.63212300	0.57345300
H	1.48971000	2.75723100	-0.45453700
C	0.19535000	1.48317700	0.70507700
C	-1.13870800	1.56332200	1.01827600
H	-1.61882700	2.52581800	1.11931700
H	-1.67254100	0.45040600	-1.18754000
H	-1.75283800	0.68973600	1.17829100
C	0.89136200	0.27889600	0.56632800
H	1.95156200	0.33120600	0.34448000
C	0.32652100	-1.08629900	0.71874100
H	0.75950700	-1.58839900	1.58485600
H	-0.75515900	-1.07661400	0.80939700

S	0.78663100	-2.05988000	-0.76730200
H	0.28382000	-3.22896000	-0.35534100
O	-2.07615200	-0.38975000	-1.42514800
H	-1.33187900	-0.96956000	-1.62493800

RC8

C	0.73607800	2.34087500	-1.03216500
H	1.77715800	2.46015200	-0.73958800
H	0.17049000	3.20542700	-0.67668600
H	0.67611200	2.35102400	-2.12229800
C	0.14410500	1.07321100	-0.49002800
C	-1.29722700	0.84785300	-0.84432600
H	-1.91753800	1.65326600	-0.44086600
H	-1.41432500	0.87452900	-1.92942200
H	-1.68257900	-0.10383400	-0.48867600
C	0.86734700	0.23187400	0.25236600
H	1.91366000	0.46212900	0.42170800
C	0.37628400	-1.05834900	0.83623100
H	0.96562700	-1.32296300	1.71004400
H	-0.66966000	-0.99988000	1.12663100
S	0.58115200	-2.37065800	-0.42302700
H	0.24634300	-3.40307600	0.35955900
O	-1.50276800	1.65103900	2.09754900
H	-0.62200000	1.46978500	1.71613700

TS15-R4

C	0.03372200	0.06836200	-0.24112500
H	1.10561800	0.15135000	-0.07459500
H	-0.45908100	0.93558800	0.20503200
H	-0.15850600	0.11274600	-1.31472600
C	-0.52428500	-1.19685600	0.33968200
C	-1.99997800	-1.36442600	0.23775200
H	-2.49183400	-0.89076000	1.17260200
H	-2.42588800	-0.81423400	-0.59941200
H	-2.33214900	-2.39954000	0.22433800
C	0.25314800	-2.09478500	0.95072600
H	1.32440000	-1.92991200	0.97775500
C	-0.24319200	-3.35789000	1.58062300
H	0.41332100	-3.65953800	2.39232800
H	-1.25261000	-3.24175200	1.96806100
S	-0.23392800	-4.67552800	0.30774800
H	-0.55916500	-5.68975500	1.11782200
O	-2.61655500	-0.38121400	2.55342100

H	-1.66797700	-0.47241200	2.74229500

PC15

C	0.84128500	2.33321000	-1.20641500
H	1.70579200	2.06317900	-1.81327700
H	1.19536700	2.97227700	-0.39740800
H	0.15749500	2.90897100	-1.82621500
C	0.17373500	1.09430400	-0.65969400
C	-1.10930800	0.78442100	-1.02485000
H	-1.29690700	1.45920100	1.31623600
H	-1.65972300	1.42009800	-1.70363100
H	-1.59595300	-0.11924400	-0.68512600
C	0.91829700	0.28977700	0.21469600
H	1.94623300	0.56493400	0.41242300
C	0.41169800	-0.95247000	0.84915400
H	1.00417200	-1.19695200	1.72563000
H	-0.63046400	-0.85834800	1.14456600
S	0.57244700	-2.33660100	-0.35641100
H	0.25415200	-3.31953200	0.49403000
O	-1.48272800	1.28935100	2.24660400
H	-2.23869300	1.83866000	2.46303700

TS16-R5

C	-0.04305900	0.09096400	0.09311100
H	1.03017900	0.21320400	0.21464100
H	-0.58607300	0.93627700	0.51726800
H	-0.26043800	0.17816500	-1.04108600
C	-0.55558100	-1.22196500	0.56100600
C	-2.05024300	-1.35706000	0.55878400
H	-2.49105100	-0.69821600	1.31012500
H	-2.43833900	-1.03837800	-0.41071500
H	-2.38650700	-2.37424700	0.74082100
C	0.28861400	-2.20047300	0.89761200
H	1.35430200	-2.01411800	0.82144700
C	-0.10658500	-3.57595300	1.33108100
H	0.66416400	-4.01196800	1.96044300
H	-1.04744300	-3.57557800	1.87388700
S	-0.27912400	-4.60412700	-0.17505500
H	-0.41585000	-5.78792700	0.43435700
O	-0.57062200	-0.18630200	-2.43350700
H	-0.31017800	-1.11787600	-2.34084500

PC16

C	0.49856900	2.59126600	0.58369800
H	1.56204700	2.76011800	0.68431400
H	-0.12862900	3.44399500	0.36810700
H	0.14797900	1.41040600	-1.82345500
C	-0.03335200	1.33443300	0.73861200
C	-1.51977000	1.12085200	0.59603800
H	-1.96036500	0.81580200	1.54640900
H	-2.01000600	2.03492300	0.27036900
H	-1.73334600	0.34638200	-0.14138000
C	0.79944300	0.24803700	1.01566900
H	1.86503200	0.42236100	1.10025700
C	0.32792300	-1.15404200	1.11431000
H	0.91017500	-1.72025700	1.83662800
H	-0.72546200	-1.20698200	1.37403500
S	0.54839000	-1.94912800	-0.53746300
H	-0.16261200	-3.05701300	-0.29560700
O	-0.27421800	0.85509200	-2.48542600
H	-0.18481400	-0.03923500	-2.13851800

TS17-R5

C	-0.01083500	0.01806800	-0.11461200
H	1.07487900	0.06804600	-0.13567300
H	-0.37524300	0.88025600	0.56151700
H	-0.43165800	0.27502100	-1.08750100
C	-0.54153700	-1.26026200	0.42441500
C	-2.03270700	-1.40631700	0.35534500
H	-2.51046600	-0.58713200	0.89713200
H	-2.36009400	-1.33335700	-0.68350200
H	-2.38800700	-2.35426900	0.74970600
C	0.28346300	-2.17494000	0.94119700
H	1.35072500	-1.98491300	0.90788300
C	-0.12827600	-3.49111700	1.52008400
H	0.60775400	-3.83029700	2.24395200
H	-1.09843900	-3.43386900	2.00537900
S	-0.20977600	-4.71422500	0.15922800
H	-0.41389000	-5.79624500	0.92019600
O	-0.84031300	1.67010900	1.72964800
H	-0.44761100	1.08087500	2.39396700

PC17

C	0.54543800	1.80194000	-1.68323200
H	1.61732900	1.92358500	-1.75948600

H	0.14429200	1.94228900	1.50430600
H	-0.07668500	2.40669600	-2.32650600
C	-0.00126100	0.89654900	-0.80484100
C	-1.49888400	0.73909000	-0.71154200
H	-1.86074800	1.07100500	0.26300100
H	-1.99638900	1.32554800	-1.48037000
H	-1.78372400	-0.30658500	-0.83276200
C	0.82317300	0.12340200	0.01521200
H	1.89656800	0.21665800	-0.09796400
C	0.31942400	-0.88080500	0.98447000
H	1.00650300	-0.99260900	1.81871200
H	-0.66123400	-0.60800300	1.36526400
S	0.19072400	-2.50488900	0.12188500
H	-0.21224700	-3.21513800	1.18255200
O	-0.37057000	1.92363800	2.31804100
H	-0.35982600	2.82229600	2.65369700

RC9

C	0.43109200	2.42876800	-0.84673000
H	1.51230000	2.49522200	-0.73821400
H	-0.03029800	3.24896400	-0.29239400
H	0.17777200	2.57238500	-1.89915100
C	-0.10253000	1.11797700	-0.35409800
C	-1.58876000	0.96376300	-0.45050100
H	-2.07496200	1.69355500	0.19927500
H	-1.91244000	1.16883100	-1.47273900
H	-1.93199600	-0.03110600	-0.18150900
C	0.71771900	0.17244100	0.12494900
H	1.78691800	0.35792400	0.10167000
C	0.29438100	-1.16171200	0.64871300
H	1.01047000	-1.51665000	1.38470100
H	-0.68518900	-1.11214100	1.11324500
S	0.25766600	-2.34092000	-0.75154700
H	0.04309400	-3.44781300	-0.03087600
O	-0.08795600	1.45055500	2.19690000
H	0.81127400	1.81395900	2.20516400

TS18-R6

C	-0.03517900	-0.00276500	0.02562100
H	1.04381400	0.06189300	0.14209100
H	-0.49158900	0.81356100	0.59022700
H	-0.29171700	0.15424200	-1.02463100
C	-0.56394100	-1.31934900	0.50126200

C	-2.05132600	-1.48233400	0.42464400
H	-2.55022500	-0.73644900	1.04964700
H	-2.38623100	-1.29915300	-0.59851100
H	-2.39014000	-2.47335500	0.71417500
C	0.26739700	-2.26528900	0.97806000
H	1.33299400	-2.08348000	0.92388800
C	-0.14116900	-3.62390200	1.44958600
H	0.60842500	-4.01292300	2.13132400
H	-1.09881100	-3.60045300	1.96222700
S	-0.25679300	-4.72056400	-0.01107300
H	-0.41198800	-5.86930200	0.65744500
O	0.33944800	-1.24972600	3.01961500
H	-0.60784200	-1.05468900	3.08317100

TS19-R7

C	-0.03515500	0.01851700	0.03039100
H	1.03744900	0.12131500	0.18570900
H	-0.54679300	0.84806900	0.51823900
H	-0.23886700	0.09026000	-1.04036100
C	-0.55565200	-1.28140700	0.56157200
C	-2.02948300	-1.48670700	0.41140400
H	-2.56108700	-0.63200500	0.82740400
H	-2.27491700	-1.55455400	-0.65089500
H	-2.38181400	-2.39107400	0.89821500
C	0.29594100	-2.25243300	0.97383100
H	1.36031100	-2.04647700	0.94275700
C	-0.11137300	-3.60274100	1.45682800
H	0.65406000	-4.01532800	2.10770200
H	-1.04933600	-3.55300400	2.00112700
S	-0.29801000	-4.69837000	-0.00060500
H	-0.53339100	-5.82753400	0.67734200
O	-0.63388800	-0.88747300	2.72248200
H	0.26074200	-0.55539500	2.88629100

Table S2: Calculated positive frequencies (cm^{-1}) for reactants, complexes, transition states (TSs) and products at the M06-2X/aug-cc-pV(T+d)Z level of theory.

Species	Frequencies
MBT-I	40 116 137 199 238 317<br>348 403 474 519 705 771<br>840 888 967 1007 1015 1046

	1106 1137 1180 1262 1270 1375<br>1414 1422 1474 1478 1493 1496<br>1505 1765 2730 3047 3054 3097<br>3098 3101 3147 3151 3165 3173
MBT-II	50 112 129 199 244 316<br>341 393 471 539 716 788<br>856 922 953 969 1010 1040<br>1107 1132 1163 1259 1265 1373<br>1415 1423 1472 1482 1491 1496<br>1514 1763 2717 3049 3054 3100<br>3103 3106 3147 3152 3165 3177
MBT-III	48 106 124 180 206 310<br>348 397 468 547 696 771<br>839 879 965 979 1009 1057<br>1106 1140 1187 1262 1277 1374<br>1416 1422 1471 1478 1490 1496<br>1508 1765 2729 3048 3055 3099<br>3102 3104 3144 3146 3164 3168
TS1-R1	-841 42 66 101 125 149<br>199 240 292 313 348 409<br>478 514 678 723 778 834<br>889 967 979 1017 1044 1107<br>1137 1178 1253 1265 1379 1415<br>1424 1467 1471 1489 1493 1500<br>1755 1817 3048 3055 3089 3099<br>3103 3148 3155 3166 3179 3704
RC1	36 68 73 122 135 157<br>217 255 302 337 370 401<br>447 475 546 684 764 829<br>883 968 994 1011 1047 1108<br>1143 1186 1257 1281 1371 1416<br>1421 1472 1477 1493 1498 1502<br>1754 2719 3046 3054 3099 3107<br>3108 3145 3150 3157 3169 3722
PC1	34 49 87 111 125 140<br>154 208 210 306 308 317<br>393 480 534 738 790 869<br>927 971 1008 1022 1108 1123<br>1142 1223 1254 1365 1413 1419<br>1469 1478 1490 1494 1512 1619<br>1719 3051 3057 3103 3107 3111<br>3149 3155 3165 3184 3833 3934

$(CH_3)_2C{=}CHCH_2S^{\bullet}$ (P1)	76 106 137 195 306 314<br>390 469 535 739 790 868<br>926 968 1006 1022 1104 1122<br>1142 1223 1255 1366 1413 1420<br>1470 1478 1489 1493 1510 1734<br>3049 3055 3100 3100 3104 3149<br>3150 3166 3186
Water (H_2O)	1619 3868 3971
TS2-R2	-639 37 66 91 118 131<br>148 194 213 293 326 411<br>473 494 622 730 794 846<br>877 967 985 1017 1048 1070<br>1104 1156 1206 1271 1319 1389<br>1415 1423 1473 1486 1494 1497<br>1638 1781 2724 3049 3056 3099<br>3103 3150 3152 3171 3178 3770
RC2	31 60 76 119 139 144<br>194 212 321 350 367 403<br>421 476 526 694 770 843<br>889 969 1003 1013 1044 1108<br>1135 1186 1263 1278 1376 1415<br>1425 1473 1477 1491 1497 1508<br>1761 2723 3049 3054 3096 3100<br>3103 3140 3147 3161 3171 3636
PC2	23 63 86 105 122 147<br>159 174 189 249 302 309<br>376 415 432 489 710 826<br>828 911 985 986 1001 1055<br>1076 1181 1233 1322 1401 1413<br>1428 1472 1477 1490 1498 1519<br>1615 2730 3033 3038 3078 3088<br>3138 3141 3152 3214 3826 3940
$(CH_3)_2C{=}CHC^{\bullet}HSH$ (P2)	86 132 167 183 240 296<br>301 415 416 488 708 826<br>827 909 983 986 998 1052<br>1072 1183 1234 1320 1399 1413<br>1430 1473 1477 1487 1498 1519<br>2732 3029 3035 3071 3081 3135<br>3138 3153 3216
TS3-R2	-713 54 83 118 126 158<br>205 227 303 334 364 401

	458 528 643 708 784 839<br>886 970 996 1004 1019 1071<br>1107 1139 1234 1293 1355 1371<br>1415 1424 1474 1487 1494 1500<br>1703 1751 2730 3048 3051 3099<br>3102 3131 3148 3166 3168 3759
PC3	55 80 106 131 160 174<br>185 196 229 284 314 382<br>399 439 480 643 671 721<br>832 902 973 994 1004 1047<br>1079 1188 1241 1350 1395 1412<br>1428 1468 1479 1488 1505 1530<br>1614 2724 3032 3039 3076 3100<br>3139 3147 3153 3210 3807 3931
TS4-R3	-1051 62 85 113 120 144<br>201 243 286 336 359 386<br>422 512 571 719 767 846<br>919 954 971 999 1028 1073<br>1104 1158 1211 1255 1270 1403<br>1416 1433 1462 1475 1488 1495<br>1498 1765 2731 3051 3057 3090<br>3104 3108 3140 3159 3164 3751
RC3	36 70 124 135 149 167<br>212 246 314 353 399 441<br>472 497 542 677 761 830<br>884 970 994 1008 1049 1105<br>1135 1190 1261 1280 1371 1416<br>1417 1472 1475 1491 1495 1503<br>1742 2718 3053 3056 3107 3107<br>3113 3148 3154 3157 3175 3695
PC4	22 45 81 114 130 157<br>197 201 217 259 294 329<br>386 435 485 501 695 759<br>828 960 962 992 1003 1097<br>1160 1181 1227 1274 1395 1410<br>1450 1473 1485 1492 1495 1632<br>1792 2727 3051 3052 3055 3106<br>3110 3115 3154 3154 3817 3908
$(CH_3)_2C=C^{\bullet}CH_2SH$ (P3)	22 107 158 165 202 236<br>326 384 482 499 701 760<br>825 961 962 1000 1007 1097<br>1162 1180 1227 1268 1394 1408

	1454 1475 1484 1493 1495 1792 2731 3046 3049 3053 3102 3106 3109 3150 3156
TS5-R4	-673 29 62 70 85 127 200 243 304 326 351 404 470 530 670 701 780 850 891 986 989 1015 1048 1089 1134 1175 1256 1269 1297 1346 1380 1416 1470 1485 1497 1503 1652 1834 2699 3050 3099 3103 3107 3149 3151 3170 3178 3777
PC5	14 49 78 94 123 134 149 218 282 321 369 400 433 495 510 611 697 765 778 837 892 962 1008 1050 1064 1139 1195 1246 1286 1338 1409 1452 1489 1496 1502 1560 1610 2693 3066 3087 3128 3153 3154 3178 3185 3266 3790 3935
$(CH_3)(^{\bullet}CH_2)C{=}CHCH_2SH$ (P4)	71 84 107 173 305 319 429 485 525 604 655 738 787 853 884 954 1005 1054 1065 1129 1199 1253 1279 1335 1411 1453 1489 1498 1506 1566 2723 3067 3104 3127 3153 3153 3175 3192 3264
TS6-R4	-840 26 41 77 127 170 206 275 308 335 355 400 457 519 691 724 778 848 891 927 986 1007 1047 1079 1136 1181 1263 1270 1330 1358 1402 1416 1465 1483 1496 1501 1573 1762 2731 3050 3094 3099 3103 3150 3152 3172 3179 3774
PC6	41 58 78 90 102 125 147 194 271 299 330 332 427 492 525 621 662 746 786 857 883 954 1005 1054 1065 1130 1205 1251 1284 1344 1410 1453 1489 1497 1506 1565 1614 2730 3067 3113 3127 3152 3163 3173 3194 3261 3820 3943

TS7-R5	-817 16 48 53 89 129<br>153 175 282 321 369 403<br>467 526 699 735 777 838<br>873 943 981 1008 1048 1069<br>1136 1183 1258 1269 1345 1370<br>1385 1424 1466 1481 1488 1506<br>1600 1768 2723 3056 3085 3105<br>3107 3152 3163 3166 3173 3774
PC7	56 72 98 119 121 152<br>196 249 286 303 332 388<br>437 497 522 613 679 778<br>789 846 872 964 1006 1041<br>1064 1129 1212 1245 1279 1391<br>1422 1434 1488 1492 1503 1540<br>1619 2694 3065 3099 3125 3153<br>3156 3163 3185 3260 3806 3936
TS8-R5	-785 31 42 73 108 145<br>164 207 292 315 355 411<br>472 516 707 723 779 842<br>877 941 989 1011 1047 1070<br>1138 1182 1259 1268 1345 1375<br>1393 1424 1463 1481 1487 1503<br>1577 1763 2728 3058 3083 3098<br>3107 3154 3164 3168 3176 3780
PC8	42 56 80 90 122 149<br>175 184 197 290 303 331<br>436 495 522 593 671 765<br>783 843 860 956 1002 1026<br>1064 1127 1212 1245 1272 1389<br>1421 1434 1486 1493 1506 1536<br>1607 2721 3062 3105 3121 3153<br>3164 3167 3186 3264 3826 3944
TS9-R6	-163 26 47 92 115 130<br>159 187 257 320 345 400<br>470 525 608 706 771 839<br>894 966 1001 1006 1048 1097<br>1140 1184 1262 1270 1373 1415<br>1419 1469 1476 1488 1493 1501<br>1689 2724 3047 3051 3094 3102<br>3109 3152 3157 3164 3196 3797
$(CH_3)_2C(OH)=C^{\bullet}HCH_2SH$ (P5)	48 95 111 129 169 224<br>242 300 348 362 401 491

	547 706 783 839 934 965<br>984 999 1042 1059 1103 1165<br>1204 1265 1301 1321 1387 1404<br>1410 1424 1468 1479 1480 1494<br>1501 2730 2983 3004 3079 3084<br>3086 3095 3126 3130 3143 3859
TS10-R7	155 32 101 119 127 169<br>212 241 266 322 353 402<br>420 527 666 694 766 828<br>875 966 997 1002 1049 1084<br>1139 1181 1262 1270 1370 1411<br>1421 1468 1477 1488 1496 1503<br>1667 2723 3054 3060 3111 3116<br>3126 3148 3163 3172 3182 3806
$(CH_3)_2C^{\bullet}C(OH)HCH_2SH$ (P6)	68 100 225 234 252 291<br>307 339 341 364 408 443<br>542 562 667 733 821 914<br>943 978 993 1025 1122 1128<br>1180 1230 1239 1256 1347 1393<br>1408 1417 1475 1483 1490 1503<br>1512 2741 3056 3062 3084 3127<br>3138 3144 3145 3148 3197 3851

Table S3: Calculated rotational constants (GHz) for reactants, complexes, transition states (TS), and products at the M06-2X/aug-cc-pV(T+d)Z level of theory.

Species	A	B	C
MBT-I	5.88	1.30	1.21
MBT-II	5.62	1.41	1.31
MBT-III	5.64	1.39	1.28
TS1-R1	2.96	1.16	1.02
RC1	2.61	1.29	1.08
PC1	2.26	1.39	1.07
$(CH_3)_2C=CHCH_2S^{\bullet}$ (P1)	5.48	1.53	1.42
TS2-R2	2.70	0.94	0.83
RC2	2.55	1.11	0.85
PC2	2.35	1.05	0.87
$(CH_3)_2C=CHC^{\bullet}HSH$ (P2)	7.58	2.00	1.05
TS3-R2	2.50	1.20	0.98
PC3	2.44	1.28	1.09
TS4-R3	2.46	1.25	0.91
RC3	2.86	1.25	1.10

PC4	2.30	1.11	0.82
$(CH_3)_2C{=}C^{\bullet}CH_2SH$ (P3)	6.06	1.26	1.17
TS5-R4	2.14	1.35	0.96
PC5	2.40	1.21	0.98
$(CH_3)(^{\bullet}CH_2)C{=}CHCH_2SH$ (P4)	5.99	1.42	1.34
TS6-R4	3.21	0.88	0.78
PC6	2.10	1.21	0.92
TS7-R5	2.72	0.98	0.86
PC7	2.34	1.30	1.03
TS8-R5	4.62	0.74	0.72
PC8	2.21	1.16	0.92
TS9-R6	2.81	1.14	0.98
$(CH_3)_2C(OH){=}C^{\bullet}HCH_2SH$ (P5)	2.77	1.38	1.17
TS10-R7	3.29	1.10	1.02
$(CH_3)_2C^{\bullet}C(OH)HCH_2SH$ (P6)	4.01	1.10	1.07

Table S4: Imaginary frequencies of all transition states associated with the MBT + OH radical reactions obtained at the M06-2X/aug-cc-pV(T+d)Z level of theory.

Transition State	M06-2X/aug-cc-pV(T+d)Z
TS1-R1	841
TS2-R2	639
TS3-R2	713
TS4-R3	1051
TS5-R4	673
TS6-R4	840
TS7-R5	817
TS8-R5	785
TS9-R6	163
TS10-R7	155
TS11-R1	984
TS12-R2	725
TS13-R3	1047
TS14-R4	956
TS15-R4	902
TS16-R5	863
TS17-R5	796
TS18-R6	181
TS19-R7	178

Table S5: Calculated total electronic energies for reactants, pre- and post-reactive complexes, transition states (TSs), and products associated with the MBT + OH radical reaction at the M06-2X/aug-cc-pV(T+d)Z and CCSD(T)/aug-cc-pV(T+d)Z levels of theory. Zero-point energy (ZPE) corrections and thermal corrections to Gibbs free energies and enthalpies are given at the M06-2X level.

Species	M06-2X	ZPE (M06-2X)	CCSD(T)	Thermal correction to Gibbs free energy	Thermal correction to Enthalpy
MBT-I	-594.702270	0.137080	-593.927085	0.103855	0.146276
MBT-II	-594.701320	0.137089	-593.926091	0.104040	0.146275
MBT-III	-594.701082	0.136872	-593.925720	0.103562	0.146181
OH radical	-75.733809	0.008585	-75.6455842	-0.008335	0.011890
RC1	-670.445875	0.147995	-669.579797	0.110191	0.159997
TS1-R1	-670.441477	0.145986	-669.577436	0.109154	0.157224
PC1	-670.498912	0.151398	-669.632237	0.112510	0.163960
P1	-594.060617	0.127872	-593.282751	0.094890	0.136516
H_2O	-76.430105	0.021547	-76.3422883	0.003259	0.025327
TS2-R2	-670.434069	0.143644	-669.570271	0.105742	0.155321
RC2	-670.445298	0.147709	-669.580429	0.109327	0.159835
PC2	-670.500961	0.146806	-669.633810	0.107065	0.159946
P2	-594.063440	0.123269	-593.284119	0.090090	0.132417
TS3-R2	-670.436723	0.144502	-669.572452	0.108153	0.155632
PC3	-670.498261	0.147935	-669.630751	0.110478	0.160353
RC3	-670.448271	0.148455	-669.582192	0.111362	0.160177
TS4-R3	-670.433468	0.143309	-669.568693	0.106869	0.154576
PC4	-670.460199	0.147780	-669.591978	0.108207	0.160664
P3	-594.021073	0.123545	-593.242281	0.088805	0.132992
TS5-R4	-670.434763	0.144447	-669.571311	0.106482	0.155877
PC5	-670.491452	0.147379	-669.626683	0.107586	0.159996
P4	-594.078016	0.123406	-593.276655	0.089872	0.132438
TS6-R4	-670.434038	0.144468	-669.570020	0.106600	0.155717
PC6	-670.490733	0.147257	-669.625272	0.108055	0.160063

TS7-R5	-670.434527	0.143949	-669.570471	0.104636	0.155646
PC7	-670.493284	0.147792	-669.627798	0.110197	0.160092
TS8-R5	-670.434052	0.144187	-669.570065	0.105989	0.155691
PC8	-670.490974	0.146893	-669.625670	0.107826	0.159746
TS9-R6	-670.444330	0.147582	-669.580382	0.159194	0.109447
P5	-670.492009	0.151508	-669.623774	0.115529	0.162397
TS10-R7	-670.445083	0.148162	-669.581621	0.111621	0.159347
P6	-670.494168	0.151334	-669.626876	0.116897	0.161712
RC4	-670.449790	0.149687	-669.584639	0.112517	0.161173
TS11-R1	-670.438930	0.146942	-669.574654	0.109690	0.158298
PC11	-670.499908	0.151581	-669.634277	0.112553	0.164005
RC5	-670.445426	0.147996	-669.580454	0.109555	0.159899
TS12-R2	-670.435566	0.144256	-669.571315	0.107508	0.155565
PC12	-670.499584	0.147517	-669.631861	0.109598	0.160188
RC6	-670.444928	0.147834	-669.579897	0.109705	0.159873
TS13-R3	-670.431765	0.142960	-669.567104	0.105862	0.154480
PC13	-670.459507	0.147690	-669.591450	0.108118	0.160648
RC7	-670.446339	0.148102	-669.580890	0.110417	0.160073
TS14-R4	-670.436308	0.144808	-669.572394	0.108804	0.155545
PC14	-670.490442	0.147537	-669.624900	0.109225	0.159947
TS15-R4	-670.433095	0.144144	-669.568933	0.105580	0.155548
PC15	-670.488725	0.146839	-669.623559	0.107234	0.159822
TS16-R5	-670.433491	0.144195	-669.569318	0.106540	0.155527
PC16	-670.491190	0.147574	-669.625554	0.109791	0.159904
RC8	-670.443000	0.147273	-669.578166	0.159769	0.107762
TS17-R5	-670.432888	0.144203	-669.568874	0.106293	0.155627
PC17	-670.489732	0.147097	-669.624550	0.108392	0.159781
RC9	-670.444616	0.147816	-669.579814	0.109497	0.160092
TS18-R6	-670.443307	0.147402	-669.579377	0.109691	0.159128
TS19-R7	-670.443747	0.1482810	-669.580402	0.112255	0.159410

Table S6: T1 diagnostic values for all the stationary points involved in the MBT + OH radical reaction calculated at the CCSD(T)/aug-cc-pV(T+d)Z level on M06-2X optimized geometries.

Species	T1 values	Species	T1 value
MBT-I	0.010	TS7-R5	0.019
$^{\bullet}$OH	0.010	PC7	0.020
RC1	0.013	TS8-R5	0.019
TS1-R1	0.018	PC8	0.020
PC1	0.016	RC4	0.013
P1	0.017	TS9-R6	0.022
H_2O	0.010	P5	0.012
RC2	0.011	TS10-R6	0.022
TS2-R2	0.019	P6	0.013
PC2	0.020	MBT-II	0.010
P2	0.020	MBT-III	0.010
TS3-R2	0.019		
PC3	0.020		
RC3	0.011		
TS4-R3	0.021		
PC4	0.021		
P3	0.022		
TS5-R4	0.021		
PC5	0.020		
P4	0.022		
TS6-R4	0.019		
PC6	0.020		

Table S7: Rate coeffiicents for the various possible abstraction and addition channels associated with the MBT-II + OH radical reaction in the temperature range between 200 – 320 K and 1 atm pressure.

T (K)	TS11-R1	TS12-R2[a]	TS13-R3	TS14-R4	TS15-R4[a]	TS16-R5
200	5.02×10^{-12}	2.01×10^{-12}	6.29×10^{-13}	2.28×10^{-12}	7.32×10^{-13}	8.87×10^{-13}
220	4.53×10^{-12}	1.68×10^{-12}	4.45×10^{-13}	1.87×10^{-12}	7.17×10^{-13}	7.00×10^{-13}
240	4.10×10^{-12}	1.44×10^{-12}	3.39×10^{-13}	1.56×10^{-12}	7.16×10^{-13}	5.91×10^{-13}
250	3.91×10^{-12}	1.35×10^{-12}	3.03×10^{-13}	1.44×10^{-12}	7.20×10^{-13}	5.56×10^{-13}
260	3.73×10^{-12}	1.27×10^{-12}	2.76×10^{-13}	1.33×10^{-12}	7.27×10^{-13}	5.28×10^{-13}
280	3.42×10^{-12}	1.15×10^{-12}	2.38×10^{-13}	1.14×10^{-12}	7.46×10^{-13}	4.93×10^{-13}
298	3.19×10^{-12}	1.06×10^{-12}	2.17×10^{-13}	1.01×10^{-12}	7.70×10^{-13}	4.76×10^{-13}
300	3.16×10^{-12}	1.06×10^{-12}	2.16×10^{-13}	1.00×10^{-12}	7.73×10^{-13}	4.75×10^{-13}
320	2.95×10^{-12}	9.86×10^{-13}	2.04×10^{-13}	8.87×10^{-13}	8.07×10^{-13}	4.69×10^{-13}

TS17-R5[a]	TS18-R6	TS19-R7	$k_{\mathrm{MBT-II}}$[b]	X_2[c]	k_2[d]
1.36×10^{-12}	9.14×10^{-12}	8.15×10^{-12}	3.43×10^{-11}	0.11598	3.98×10^{-12}
1.37×10^{-12}	9.14×10^{-12}	7.89×10^{-12}	3.21×10^{-11}	0.13155	4.22×10^{-12}
1.41×10^{-12}	9.13×10^{-12}	7.60×10^{-12}	3.05×10^{-11}	0.14553	4.43×10^{-12}
1.44×10^{-12}	9.11×10^{-12}	7.44×10^{-12}	2.98×10^{-11}	0.15196	4.52×10^{-12}
1.47×10^{-12}	9.09×10^{-12}	7.27×10^{-12}	2.92×10^{-11}	0.15803	4.61×10^{-12}
1.54×10^{-12}	9.04×10^{-12}	6.94×10^{-12}	2.81×10^{-11}	0.16921	4.76×10^{-12}
1.61×10^{-12}	8.98×10^{-12}	6.63×10^{-12}	2.74×10^{-11}	0.17826	4.88×10^{-12}
1.62×10^{-12}	8.98×10^{-12}	6.59×10^{-12}	2.73×10^{-11}	0.17921	4.90×10^{-12}
1.71×10^{-12}	8.90×10^{-12}	6.25×10^{-12}	2.67×10^{-11}	0.18819	5.02×10^{-12}

[a]The degeneracy of TS12-R2, TS15-R4, and TS17-R5 is 2 and thus the contributions of the rate coefficients for these paths was multiplied by a factor of 2; [b]$k_{\mathrm{MBT-II}}$ refers to the total rate coefficient for the MBT-II + OH radical reaction obtained by adding the corresponding site-specific reaction rate coefficients at each temperature; [c]the mole fraction of MBT-II was calculated using eqn. 3; [d]the total rate coefficient (k_2) was calculated by multiplying $k_{\mathrm{MBT-II}}$ by X_2 at the present studied temperatures.

Table S8: Boltzmann weight factors and mole fractions of the MBT-III conformer, and calculated total rate coefficients for the MBT-III + OH radical reaction.

T (K)	Γ1	Γ2	Γ3	Γ1+ Γ2+ Γ3	X_3	k_3[a]
200	1.0	0.15532	0.18388	1.33920	0.13730	4.71×10^{-12}
220	1.0	0.18397	0.21448	1.39845	0.15337	4.92×10^{-12}
240	1.0	0.21185	0.24384	1.45569	0.16751	5.10×10^{-12}
250	1.0	0.22542	0.25800	1.48342	0.17392	5.18×10^{-12}
260	1.0	0.23871	0.27180	1.51051	0.17994	5.25×10^{-12}
280	1.0	0.26443	0.29831	1.56273	0.19089	5.37×10^{-12}
298	1.0	0.28655	0.32092	1.60747	0.19964	5.47×10^{-12}
300	1.0	0.28895	0.32336	1.61231	0.20056	5.48×10^{-12}
320	1.0	0.31226	0.34700	1.65926	0.20913	5.58×10^{-12}

[a]The mole fraction of MBT-III was calculated using eqn. 4; [b]The total rate coefficient (k_3) was calculated by multiplying $k_{\mathrm{MBT-III}}$ by X_3 at the present studied temperatures.

Table S9: Lower vibrational modes (in cm^{-1}) for various stationary points treated as hindered rotors at the M06-2X/aug-cc-pV(T+d)Z level of theory.

Species	M06-2X/aug-cc-pV(T+d)Z
MBT-I	40.3, 116.0, 137.2
TS1-R1	66.1, 101.2
TS2-R2	37.2, 91.5
TS3-R2	54.1
TS4-R3	61.6
TS5-R4	69.7, 200.1
TS6-R4	25.7
TS7-R5	48.4
TS8-R5	41.7
TS9-R6	26.2, 47.2, 91.8
TS10-R7	32.1, 101.1, 119.0

Table S10: Rate coeffiicents for the various possible abstraction and addition channels associated with the MBT + OH radical reaction with the hindered rotor (HR) treatment in the temperature range between 200 – 320 K and 1 atm pressure.

T (K)	TS1-R1	TS2-R2	TS3-R2	TS4-R3	TS5-R4[a]	TS6-R4	TS7-R5
200	4.26×10^{-11}	8.65×10^{-13}	8.79×10^{-13}	2.15×10^{-13}	7.03×10^{-13}	5.80×10^{-12}	2.39×10^{-12}
220	3.76×10^{-11}	7.48×10^{-13}	7.55×10^{-13}	1.60×10^{-13}	6.82×10^{-13}	3.31×10^{-12}	2.32×10^{-12}
240	3.33×10^{-11}	6.84×10^{-13}	6.66×10^{-13}	1.28×10^{-13}	6.84×10^{-13}	2.17×10^{-12}	2.30×10^{-12}
250	3.14×10^{-11}	6.66×10^{-13}	6.31×10^{-13}	1.17×10^{-13}	6.91×10^{-13}	1.83×10^{-12}	2.30×10^{-12}
260	2.97×10^{-11}	6.53×10^{-13}	6.00×10^{-13}	1.09×10^{-13}	7.02×10^{-13}	1.57×10^{-12}	2.31×10^{-12}
280	2.66×10^{-11}	6.42×10^{-13}	5.52×10^{-13}	9.76×10^{-14}	7.31×10^{-13}	1.23×10^{-12}	2.35×10^{-12}
298	2.42×10^{-11}	6.44×10^{-13}	5.19×10^{-13}	9.20×10^{-14}	7.65×10^{-13}	1.04×10^{-12}	2.40×10^{-12}
300	2.39×10^{-11}	6.44×10^{-13}	5.16×10^{-13}	9.16×10^{-14}	7.69×10^{-13}	1.02×10^{-12}	2.40×10^{-12}
320	2.17×10^{-11}	6.56×10^{-13}	4.88×10^{-13}	8.91×10^{-14}	8.15×10^{-13}	8.87×10^{-13}	2.48×10^{-12}

TS8-R5[a]	TS9-R6	TS10-R7	k_{global}[b]
6.04×10^{-13}	1.90×10^{-11}	3.37×10^{-10}	4.03×10^{-10}
5.17×10^{-13}	1.94×10^{-11}	2.75×10^{-10}	3.33×10^{-10}
4.76×10^{-13}	1.97×10^{-11}	2.25×10^{-10}	2.77×10^{-10}
4.66×10^{-13}	1.99×10^{-11}	2.04×10^{-10}	2.54×10^{-10}
4.61×10^{-13}	2.00×10^{-11}	1.85×10^{-10}	2.33×10^{-10}
4.62×10^{-13}	2.03×10^{-11}	1.52×10^{-10}	1.97×10^{-10}
4.71×10^{-13}	2.06×10^{-11}	1.28×10^{-10}	1.71×10^{-10}
4.73×10^{-13}	2.06×10^{-11}	1.26×10^{-10}	1.69×10^{-10}
4.92×10^{-13}	2.08×10^{-11}	1.05×10^{-10}	1.46×10^{-10}

[a]The degeneracy of TS5-R4 and TS8-R5 is 2 and thus the contribution of the rate coefficients for TS5-R4 and TS8-R5 were multiplied by a factor of 2. [b]The global rate coefficients (k_{global}) for the MBT + OH radical reaction were obtained by adding the rate coefficients for all the individual reaction paths at the corresponding temperatures.

Table S11. The rate coefficients for the various H-abstraction and addition paths with the introduction of an uncertainity of 1 kcal mol^{-1} in the CCSD(T) barriers for the MBT + OH radical reaction, calculated in the temperature range between 200 – 320 K and 1 atm pressure.

T (K)	TS1-R1	TS2-R2	TS3-R2	TS4-R3	TS5-R4[a]	TS6-R4	TS7-R5	TS8-R5[a]	TS9-R6	TS10-R7	k_{global}[b]
200	3.06×10^{-11}	3.91×10^{-13}	3.45×10^{-13}	1.55×10^{-13}	2.10×10^{-13}	3.13×10^{-12}	5.02×10^{-13}	2.59×10^{-13}	1.76×10^{-11}	1.27×10^{-10}	1.81×10^{-10}
220	2.58×10^{-11}	2.81×10^{-13}	2.63×10^{-13}	1.03×10^{-13}	1.70×10^{-13}	1.46×10^{-12}	4.60×10^{-13}	1.77×10^{-13}	1.76×10^{-11}	9.82×10^{-11}	1.45×10^{-10}
240	2.20×10^{-11}	2.23×10^{-13}	2.14×10^{-13}	7.24×10^{-14}	1.54×10^{-13}	7.92×10^{-13}	4.60×10^{-13}	1.37×10^{-13}	1.76×10^{-11}	7.68×10^{-11}	1.19×10^{-10}
250	2.04×10^{-11}	2.06×10^{-13}	1.97×10^{-13}	6.24×10^{-14}	1.52×10^{-13}	6.17×10^{-13}	4.70×10^{-13}	1.25×10^{-13}	1.76×10^{-11}	6.83×10^{-11}	1.08×10^{-10}
260	1.90×10^{-11}	1.94×10^{-13}	1.83×10^{-13}	5.46×10^{-14}	1.53×10^{-13}	4.98×10^{-13}	4.86×10^{-13}	1.18×10^{-13}	1.76×10^{-11}	6.10×10^{-11}	9.95×10^{-11}
280	1.65×10^{-11}	1.81×10^{-13}	1.65×10^{-13}	4.40×10^{-14}	1.62×10^{-13}	3.56×10^{-13}	5.30×10^{-13}	1.13×10^{-13}	1.76×10^{-11}	4.92×10^{-11}	8.52×10^{-11}
298	1.47×10^{-11}	1.79×10^{-13}	1.54×10^{-13}	3.82×10^{-14}	1.75×10^{-13}	2.88×10^{-13}	5.81×10^{-13}	1.15×10^{-13}	1.76×10^{-11}	4.11×10^{-11}	7.53×10^{-11}
300	1.46×10^{-11}	1.79×10^{-13}	1.53×10^{-13}	3.78×10^{-14}	1.77×10^{-13}	2.82×10^{-13}	5.88×10^{-13}	1.16×10^{-13}	1.76×10^{-11}	4.03×10^{-11}	7.43×10^{-11}
320	1.29×10^{-11}	1.84×10^{-13}	1.47×10^{-13}	3.43×10^{-14}	1.98×10^{-13}	2.42×10^{-13}	6.56×10^{-13}	1.24×10^{-13}	1.76×10^{-11}	3.36×10^{-11}	6.61×10^{-11}

[a]The degeneracy of TS5-R4 and TS8-R5 is 2 and thus the contribution of the rate coefficients for TS5-R4 and TS8-R5 were multiplied by a factor of 2. [b]The global rate coefficients (k_{global}) for the MBT + OH radical reaction were obtained by adding the rate coefficients for all the individual reaction paths at the corresponding temperatures.

Table S12: MESMER input files for the various possible abstraction and addition channels associated with the MBT-I + OH radical reaction.

Mesmer script for TS1

```
<?xml version="1.0" encoding="utf-8" ?>
<?xml-stylesheet type='text/xsl' href='../../mesmer2.xsl' media='other'?>
<?xml-stylesheet type='text/xsl' href='../../mesmer1.xsl' media='screen'?>
<me:mesmer xmlns="http://www.xml-cml.org/schema"
xmlns:me="http://www.chem.leeds.ac.uk/mesmer"
xmlns:xsi="http://www.w3.org/2001/XMLSchema-instance" xmlns:cml="http://www.xml-
cml.org/schema">
<me:title>321MBT_OH_TS1</me:title>
<moleculeList>
  <molecule id="321MBT">
    <propertyList>
      <property dictRef="me:ZPE">
        <scalar units="kcal/mol">0.0</scalar>
      </property>
      <property dictRef="me:rotConsts">
        <array units="cm-1">0.19625 0.04349 0.04035</array>
      </property>
      <property dictRef="me:vibFreqs">
        <array units="cm-1">40.2728 115.9719 137.1856 199.4724 238.1814 316.9590
347.5624 402.8403 474.4488 519.2721 704.7871 770.9793 839.5900 887.5999 966.7843
1007.2573 1014.6785 1046.3552 1106.3000 1137.0336 1180.0570 1262.0525 1270.0480
1375.0776 1414.1651 1421.7355 1473.8347 1477.9148 1492.9447 1495.8890 1504.7109
1764.7289 2730.2078 3047.3869 3054.1175 3097.3687 3097.8866 3100.6151 3147.0004
3151.3191 3164.8649 3173.6496</array>
      </property>
      <property dictRef="me:frequenciesScaleFactor">
       <scalar>1.0</scalar>
      </property>
      <property dictRef="me:symmetryNumber">
        <scalar>1</scalar>
      </property>
      <property dictRef="me:MW">
        <scalar units="amu">102.05032</scalar>
      </property>
      <property dictRef="me:spinMultiplicity">
        <scalar>1</scalar>
      </property>
    </propertyList>
    <me:DOSCMethod name="ClassicalRotors"/>
  </molecule>
  <molecule id="OH">
```

<propertyList>
<property dictRef="me:ZPE">
<scalar units="kcal/mol">0.0</scalar>
</property>
<property dictRef="me:rotConsts">
<array units="cm-1">18.83025</array>
</property>
<property dictRef="me:vibFreqs">
<array units="cm-1">3768.5403</array>
</property>
<property dictRef="me:frequenciesScaleFactor">
<scalar>1.0</scalar>
</property>
<property dictRef="me:symmetryNumber">
<scalar>1</scalar>
</property>
<property dictRef="me:MW">
<scalar units="amu">17.00274</scalar>
</property>
<property dictRef="me:spinMultiplicity">
<scalar>2</scalar>
</property>
</propertyList>
<me:DOSCMethod name="ClassicalRotors"/>
</molecule>
<molecule id="RC1">
<propertyList>
<property dictRef="me:ZPE">
<scalar units="kcal/mol">-3.0</scalar>
</property>
<property dictRef="me:rotConsts">
<array units="cm-1">0.08699 0.04316 0.03617</array>
</property>
<property dictRef="me:vibFreqs">
<array units="cm-1">35.8244 67.5886 72.8471 122.4920 134.5751 156.6399
217.2786 255.0761 301.6535 336.9823 370.1718 400.8085 447.0932 474.5829 545.6857
684.2300 763.5315 829.0335 883.0211 968.2325 993.8452 1011.0493 1047.0903 1107.8475
1133.4125 1185.6253 1257.4277 1280.8523 1371.0997 1415.5526 1420.7184 1472.0298
1476.8668 1492.6928 1497.9832 1501.8395 1753.7393 2718.8594 3045.7800 3053.9183
3098.5793 3106.6719 3108.3600 3145.2134 3149.7155 3156.6792 3169.2441
3722.1643</array>
</property>
<property dictRef="me:frequenciesScaleFactor">
<scalar>1.0</scalar>
</property>
<property dictRef="me:symmetryNumber">

<scalar>1</scalar>
</property>
<property dictRef="me:MW">
<scalar units="amu">119.05306</scalar>
</property>
<property dictRef="me:spinMultiplicity">
<scalar>2</scalar>
</property>
<property dictRef="me:epsilon">
<scalar>306.5</scalar>
</property>
<property dictRef="me:sigma">
<scalar>4.42</scalar>
</property>
</propertyList>
<me:DOSCMethod name="ClassicalRotors"/>
<me:energyTransferModel xsi:type="me:ExponentialDown">
<me:deltaEDown units="cm-1">200</me:deltaEDown>
</me:energyTransferModel>
</molecule>
<molecule id="TS1">
<propertyList>
<property dictRef="me:ZPE">
<scalar units="kcal/mol">-2.8</scalar>
</property>
<property dictRef="me:rotConsts">
<array units="cm-1">0.09892 0.03862 0.03410</array>
</property>
<property dictRef="me:vibFreqs">
<array units="cm-1">42.0949 66.0832 101.1661 125.1211 149.2984 199.2532 239.7764 291.9091 312.9310 347.7784 408.6016 478.4386 513.6131 677.7916 721.6769 777.7024 834.2156 888.6888 966.8135 978.5449 1016.6894 1044.4787 1106.7130 1137.1491 1177.7503 1253.4574 1264.7619 1379.1132 1415.3825 1424.0299 1466.7154 1471.2219 1489.3623 1493.3814 1500.3525 1755.1678 1816.9381 3048.0655 3055.1320 3089.2471 3098.7352 3102.5719 3148.0541 3155.4809 3166.3512 3179.0713 3703.6759</array>
</property>
<property dictRef="me:frequenciesScaleFactor">
<scalar>1.0</scalar>
</property>
<property dictRef="me:symmetryNumber">
<scalar>1</scalar>
</property>
<property dictRef="me:spinMultiplicity">
<scalar>2</scalar>
</property>
<property dictRef="me:imFreqs">

<array units="cm-1">841.2870</array>
</property>
</propertyList>
<me:DOSCMethod name="ClassicalRotors"/>
</molecule>
<molecule id="PC1">
<propertyList>
<property dictRef="me:ZPE">
<scalar units="kcal/mol">-33.8</scalar>
</property>
<property dictRef="me:rotConsts">
<array units="cm-1">0.07527 0.04649 0.03575</array>
</property>
<property dictRef="me:vibFreqs">
<array units="cm-1">33.9848 49.4617 86.8056 111.0586 125.1645 140.1312 154.2511
207.9138 209.6167 306.3430 308.4153 316.7423 393.0498 479.7644 533.7893 737.6537
789.7403 868.5491 927.1859 970.7603 1007.5822 1022.0066 1108.0001 1122.6240 1141.5243
1223.4369 1254.2351 1365.2863 1413.6310 1418.5516 1469.0884 1477.9441 1489.9130
1493.5207 1511.5155 1619.1846 1719.2404 3051.1728 3056.9746 3103.1631 3107.0268
3111.4326 3148.5694 3154.7557 3164.7785 3183.9198 3833.0212 3933.5262</array>
</property>
<property dictRef="me:frequenciesScaleFactor">
<scalar>1.0</scalar>
</property>
<property dictRef="me:symmetryNumber">
<scalar>1</scalar>
</property>
<property dictRef="me:MW">
<scalar units="amu">119.05306</scalar>
</property>
<property dictRef="me:spinMultiplicity">
<scalar>2</scalar>
</property>
<property dictRef="me:epsilon">
<scalar>306.5</scalar>
</property>
<property dictRef="me:sigma">
<scalar>4.42</scalar>
</property>
</propertyList>
<me:DOSCMethod name="ClassicalRotors"/>
<me:energyTransferModel xsi:type="me:ExponentialDown">
<me:deltaEDown units="cm-1">200</me:deltaEDown>
</me:energyTransferModel>
</molecule>
<molecule id="P1">

<propertyList>
<property dictRef="me:ZPE">
<scalar units="kcal/mol">-30.5</scalar>
</property>
<property dictRef="me:rotConsts">
<array units="cm-1">0.18295 0.05091 0.04745</array>
</property>
<property dictRef="me:vibFreqs">
<array units="cm-1">76.1362 105.5411 138.7874 194.5983 305.8958 314.2413 390.0590 469.3094 534.9709 738.9309 790.0075 868.4685 925.6677 968.1145 1006.4569 1021.9085 1104.4419 1121.6659 1142.2676 1223.4815 1254.8085 1366.3424 1413.7055 1420.3833 1469.9065 1478.1165 1489.0891 1492.5590 1510.3855 1733.5672 3049.1930 3054.7838 3099.5313 3100.3730 3104.2002 3148.9958 3150.1356 3165.8914 3186.1982</array>
</property>
<property dictRef="me:frequenciesScaleFactor">
<scalar>1</scalar>
</property>
<property dictRef="me:symmetryNumber">
<scalar>1</scalar>
</property>
<property dictRef="me:MW">
<scalar units="amu">101.04250</scalar>
</property>
<property dictRef="me:spinMultiplicity">
<scalar>2</scalar>
</property>
</propertyList>
<me:DOSCMethod name="ClassicalRotors"/>
</molecule>
<molecule id="H2O">
<propertyList>
<property dictRef="me:ZPE">
<scalar units="kcal/mol">0.0</scalar>
</property>
<property dictRef="me:rotConsts">
<array units="cm-1">27.82574 14.37796 9.47967</array>
</property>
<property dictRef="me:vibFreqs">
<array units="cm-1">1618.8285 3867.7498 3971.3807</array>
</property>
<property dictRef="me:frequenciesScaleFactor">
<scalar>1.0</scalar>
</property>
<property dictRef="me:symmetryNumber">
<scalar>1</scalar>

```
        </property>
        <property dictRef="me:MW">
          <scalar units="amu">18.01056</scalar>
        </property>
        <property dictRef="me:spinMultiplicity">
          <scalar>1</scalar>
        </property>
      </propertyList>
      <me:DOSCMethod name="ClassicalRotors"/>
    </molecule>
    <molecule id="N2">
      <propertyList>
        <property dictRef="me:MW">
          <scalar units="amu">28</scalar>
        </property>
        <property dictRef="me:epsilon">
          <scalar>48</scalar>
        </property>
        <property dictRef="me:sigma">
          <scalar>3.9</scalar>
        </property>
      </propertyList>
      <me:DOSCMethod name="ClassicalRotors"/>
    </molecule>
  </moleculeList>
  <reactionList>
    <reaction id="R0">
      <reactant>
        <molecule ref="OH" me:type="deficientReactant" />
      </reactant>
      <reactant>
        <molecule ref="321MBT" me:type="excessReactant" />
      </reactant>
      <product>
        <molecule ref="RC1" me:type="modelled" />
      </product>
      <me:excessReactantConc>1.00E11</me:excessReactantConc>
      <me:MCRCMethod name="MesmerILT"/>
      <me:preExponential units="cm3molecule-1s-1">1.00E-11</me:preExponential>
      <me:activationEnergy units="kcal/mol">0.01</me:activationEnergy>
      <me:TInfinity>298.0</me:TInfinity>
      <me:nInfinity>0.1</me:nInfinity>
    </reaction>
    <reaction id="R1">
      <reactant>
        <molecule ref="RC1" me:type="modelled" />
```

```
      </reactant>
      <product>
        <molecule ref="PC1" me:type="modelled" />
      </product>
      <me:MCRCMethod name="SimpleRRKM"/>
      <me:transitionState>
        <molecule ref="TS1" me:type="transitionState" />
      </me:transitionState>
      <me:tunneling name="Eckart"/>
      <me:MCRCMethod name="SimpleRRKM"/>
    </reaction>
    <reaction id="R2">
      <reactant>
        <molecule ref="PC1" me:type="modelled" />
      </reactant>
      <product>
        <molecule ref="P1" me:type="sink" />
      </product>
      <product>
        <molecule ref="H2O" me:type="sink" />
      </product>
      <me:MCRCMethod name="MesmerILT" xsi:type="MesmerILT" >
        <me:preExponential units="cm3molecule-1s-1">1.00E-11</me:preExponential>
        <me:activationEnergy units="kcal/mol" reverse="true">0.01</me:activationEnergy>
    </me:MCRCMethod>
      </reaction>
  </reactionList>
  <me:conditions>
    <me:bathGas>N2</me:bathGas>
    <me:PTs>
      <me:PTpair me:units="Torr" me:P="760" me:T="200" me:precision= "qd" />
      <me:PTpair me:units="Torr" me:P="760" me:T="220" me:precision= "qd" />
      <me:PTpair me:units="Torr" me:P="760" me:T="240" me:precision= "qd" />
      <me:PTpair me:units="Torr" me:P="760" me:T="250" me:precision= "qd" />
      <me:PTpair me:units="Torr" me:P="760" me:T="260" me:precision= "qd" />
      <me:PTpair me:units="Torr" me:P="760" me:T="280" me:precision= "qd" />
      <me:PTpair me:units="Torr" me:P="760" me:T="298" me:precision= "qd" />
      <me:PTpair me:units="Torr" me:P="760" me:T="300" me:precision= "qd" />
      <me:PTpair me:units="Torr" me:P="760" me:T="320" me:precision= "qd" />
    </me:PTs>
    <me:InitialPopulation>
      <molecule ref="OH" me:population="1.0"/>
    </me:InitialPopulation>
  </me:conditions>
  <me:modelParameters>
    <me:grainSize units="cm-1">20</me:grainSize>
```

<me:energyAboveTheTopHill>25</me:energyAboveTheTopHill>
</me:modelParameters>
<me:control>
<me:eigenvalues>1</me:eigenvalues>
<me:calcMethod>simpleCalc</me:calcMethod>
<me:testMicroRates/>
<me:testRateConstants/>
<me:printGrainDOS/>
<me:printGrainkfE/>
<me:testDOS/>
<me:printSpeciesProfile/>
<me:printGrainedSpeciesProfile/>
</me:control>
</me:mesmer>

Mesmer script for TS2

<?xml version="1.0" encoding="utf-8" ?>
<?xml-stylesheet type='text/xsl' href='../../mesmer2.xsl' media='other'?>
<?xml-stylesheet type='text/xsl' href='../../mesmer1.xsl' media='screen'?>
<me:mesmer xmlns="http://www.xml-cml.org/schema"
xmlns:me="http://www.chem.leeds.ac.uk/mesmer"
xmlns:xsi="http://www.w3.org/2001/XMLSchema-instance" xmlns:cml="http://www.xml-cml.org/schema">
<me:title>321MBT_OH_TS2</me:title>
<moleculeList>
<molecule id="321MBT">
<propertyList>
<property dictRef="me:ZPE">
<scalar units="kcal/mol">0.0</scalar>
</property>
<property dictRef="me:rotConsts">
<array units="cm-1">0.19625 0.04349 0.04035</array>
</property>
<property dictRef="me:vibFreqs">
<array units="cm-1">40.2728 115.9719 137.1856 199.4724 238.1814 316.9590 347.5624 402.8403 474.4488 519.2721 704.7871 770.9793 839.5900 887.5999 966.7843 1007.2573 1014.6785 1046.3552 1106.3000 1137.0336 1180.0570 1262.0525 1270.0480 1375.0776 1414.1651 1421.7355 1473.8347 1477.9148 1492.9447 1495.8890 1504.7109 1764.7289 2730.2078 3047.3869 3054.1175 3097.3687 3097.8866 3100.6151 3147.0004 3151.3191 3164.8649 3173.6496</array>
</property>
<property dictRef="me:frequenciesScaleFactor">
<scalar>1.0</scalar>
</property>
<property dictRef="me:symmetryNumber">

```
      <scalar>1</scalar>
    </property>
    <property dictRef="me:MW">
      <scalar units="amu">102.05032</scalar>
    </property>
    <property dictRef="me:spinMultiplicity">
      <scalar>1</scalar>
    </property>
  </propertyList>
  <me:DOSCMethod name="ClassicalRotors"/>
</molecule>
<molecule id="OH">
  <propertyList>
    <property dictRef="me:ZPE">
      <scalar units="kcal/mol">0.0</scalar>
    </property>
    <property dictRef="me:rotConsts">
      <array units="cm-1">18.83025</array>
    </property>
    <property dictRef="me:vibFreqs">
      <array units="cm-1">3768.5403</array>
    </property>
    <property dictRef="me:frequenciesScaleFactor">
     <scalar>1.0</scalar>
    </property>
    <property dictRef="me:symmetryNumber">
      <scalar>1</scalar>
    </property>
    <property dictRef="me:MW">
      <scalar units="amu">17.00274</scalar>
    </property>
    <property dictRef="me:spinMultiplicity">
      <scalar>2</scalar>
    </property>
  </propertyList>
  <me:DOSCMethod name="ClassicalRotors"/>
</molecule>
<molecule id="RC2">
  <propertyList>
    <property dictRef="me:ZPE">
     <scalar units="kcal/mol">-3.6</scalar>
    </property>
    <property dictRef="me:rotConsts">
      <array units="cm-1">0.08517 0.03711 0.02850</array>
    </property>
    <property dictRef="me:vibFreqs">
```

<array units="cm-1">30.9451 59.5534 76.3263 119.2061 138.7491 143.7835 194.2656 211.8785 321.4811 350.3024 366.8120 403.1757 420.7014 476.0277 526.3855 694.4637 769.5880 842.5191 888.7272 969.1120 1002.7876 1012.6180 1043.9410 1107.5784 1135.2397 1185.8401 1263.2303 1278.1043 1376.2153 1415.4743 1424.7868 1473.0708 1477.4287 1491.0373 1496.6969 1508.3204 1760.6348 2722.9847 3048.8728 3054.3520 3096.4679 3099.9312 3102.7838 3139.8262 3146.6642 3160.5663 3170.6882 3636.4704</array>
</property>
<property dictRef="me:frequenciesScaleFactor">
<scalar>1.0</scalar>
</property>
<property dictRef="me:symmetryNumber">
<scalar>1</scalar>
</property>
<property dictRef="me:MW">
<scalar units="amu">119.05306</scalar>
</property>
<property dictRef="me:spinMultiplicity">
<scalar>2</scalar>
</property>
<property dictRef="me:epsilon">
<scalar>306.5</scalar>
</property>
<property dictRef="me:sigma">
<scalar>4.42</scalar>
</property>
</propertyList>
<me:DOSCMethod name="ClassicalRotors"/>
<me:energyTransferModel xsi:type="me:ExponentialDown">
<me:deltaEDown units="cm-1">200</me:deltaEDown>
</me:energyTransferModel>
</molecule>
<molecule id="TS2">
<propertyList>
<property dictRef="me:ZPE">
<scalar units="kcal/mol">0.2</scalar>
</property>
<property dictRef="me:rotConsts">
<array units="cm-1">0.09008 0.03140 0.02766</array>
</property>
<property dictRef="me:vibFreqs">
<array units="cm-1">37.2233 66.0951 91.4529 117.5456 130.7354 148.0437 194.1105 213.3783 292.8273 325.8488 411.4233 473.0285 493.9977 621.6636 729.7601 794.2550 845.6756 876.7480 965.9339 984.8741 1016.7366 1048.1924 1070.1797 1104.4464 1156.0603 1205.8746 1270.6841 1319.2335 1389.3366 1414.8371 1422.8234 1472.7195 1485.7538

1493.8369 1496.8494 1637.8881 1780.5584 2724.2276 3048.6126 3056.4088 3099.2003
3103.0540 3150.0282 3152.2242 3170.5693 3177.6220 3770.0306</array>
</property>
<property dictRef="me:frequenciesScaleFactor">
<scalar>1.0</scalar>
</property>
<property dictRef="me:symmetryNumber">
<scalar>1</scalar>
</property>
<property dictRef="me:spinMultiplicity">
<scalar>2</scalar>
</property>
<property dictRef="me:imFreqs">
<array units="cm-1">639.3869</array>
</property>
</propertyList>
<me:DOSCMethod name="ClassicalRotors"/>
</molecule>
<molecule id="PC2">
<propertyList>
<property dictRef="me:ZPE">
<scalar units="kcal/mol">-37.7</scalar>
</property>
<property dictRef="me:rotConsts">
<array units="cm-1">0.07832 0.03496 0.02899</array>
</property>
<property dictRef="me:vibFreqs">
<array units="cm-1">23.0995 63.3937 85.5867 104.7036 121.7524 146.7136
159.0430 173.7303 188.6086 248.7931 301.7819 308.8988 375.9968 415.0212 431.8803
489.4436 709.6454 826.2063 827.8796 911.0493 984.8586 986.0326 1001.2220 1054.7847
1076.1949 1181.1402 1232.6067 1321.5332 1400.5774 1412.8669 1428.4705 1471.5487
1476.8718 1489.6631 1497.7943 1519.2144 1615.2924 2729.6385 3032.6506 3037.5521
3077.5323 3087.8488 3137.7997 3141.0299 3152.3600 3214.1938 3826.3140
3939.6610</array>
</property>
<property dictRef="me:frequenciesScaleFactor">
<scalar>1.0</scalar>
</property>
<property dictRef="me:symmetryNumber">
<scalar>1</scalar>
</property>
<property dictRef="me:MW">
<scalar units="amu">119.05306</scalar>
</property>
<property dictRef="me:spinMultiplicity">
<scalar>2</scalar>

</property>
<property dictRef="me:epsilon">
<scalar>306.5</scalar>
</property>
<property dictRef="me:sigma">
<scalar>4.42</scalar>
</property>
</propertyList>
<me:DOSCMethod name="ClassicalRotors"/>
<me:energyTransferModel xsi:type="me:ExponentialDown">
<me:deltaEDown units="cm-1">200</me:deltaEDown>
</me:energyTransferModel>
</molecule>
<molecule id="P2">
<propertyList>
<property dictRef="me:ZPE">
<scalar units="kcal/mol">-34.3</scalar>
</property>
<property dictRef="me:rotConsts">
<array units="cm-1">0.25302 0.04002 0.03500</array>
</property>
<property dictRef="me:vibFreqs">
<array units="cm-1">85.9610 131.5048 167.2374 183.4367 239.9712 296.4347
301.4346 414.9136 416.4955 487.9792 707.6603 826.1410 827.4161 908.9132 982.6487
986.0236 997.9192 1051.9220 1071.8936 1183.2122 1233.6460 1320.0262 1399.3124
1413.0270 1429.8451 1472.6476 1477.2247 1487.1721 1498.3179 1518.7724 2731.6411
3029.0204 3035.3446 3070.8564 3080.9120 3135.1767 3137.6291 3153.3105
3215.6986</array>
</property>
<property dictRef="me:frequenciesScaleFactor">
<scalar>1</scalar>
</property>
<property dictRef="me:symmetryNumber">
<scalar>1</scalar>
</property>
<property dictRef="me:MW">
<scalar units="amu">101.04250</scalar>
</property>
<property dictRef="me:spinMultiplicity">
<scalar>2</scalar>
</property>
</propertyList>
<me:DOSCMethod name="ClassicalRotors"/>
</molecule>
<molecule id="H2O">
<propertyList>

```
      <property dictRef="me:ZPE">
        <scalar units="kcal/mol">0.0</scalar>
      </property>
      <property dictRef="me:rotConsts">
        <array units="cm-1">27.82574 14.37796 9.47967</array>
      </property>
      <property dictRef="me:vibFreqs">
        <array units="cm-1">1618.8285 3867.7498 3971.3807</array>
      </property>
      <property dictRef="me:frequenciesScaleFactor">
        <scalar>1.0</scalar>
      </property>
      <property dictRef="me:symmetryNumber">
        <scalar>1</scalar>
      </property>
      <property dictRef="me:MW">
        <scalar units="amu">18.01056</scalar>
      </property>
      <property dictRef="me:spinMultiplicity">
        <scalar>1</scalar>
      </property>
    </propertyList>
    <me:DOSCMethod name="ClassicalRotors"/>
  </molecule>
  <molecule id="N2">
    <propertyList>
      <property dictRef="me:MW">
        <scalar units="amu">28</scalar>
      </property>
      <property dictRef="me:epsilon">
        <scalar>48</scalar>
      </property>
      <property dictRef="me:sigma">
        <scalar>3.9</scalar>
      </property>
    </propertyList>
    <me:DOSCMethod name="ClassicalRotors"/>
  </molecule>
</moleculeList>
<reactionList>
  <reaction id="R0">
    <reactant>
      <molecule ref="OH" me:type="deficientReactant" />
    </reactant>
    <reactant>
      <molecule ref="321MBT" me:type="excessReactant" />
```

```
      </reactant>
      <product>
        <molecule ref="RC2" me:type="modelled" />
      </product>
      <me:excessReactantConc>1.00E11</me:excessReactantConc>
      <me:MCRCMethod name="MesmerILT"/>
      <me:preExponential units="cm3molecule-1s-1">1.00E-11</me:preExponential>
      <me:activationEnergy units="kcal/mol">0.01</me:activationEnergy>
      <me:TInfinity>298.0</me:TInfinity>
      <me:nInfinity>0.1</me:nInfinity>
    </reaction>
    <reaction id="R1">
      <reactant>
        <molecule ref="RC2" me:type="modelled" />
      </reactant>
      <product>
        <molecule ref="PC2" me:type="modelled" />
      </product>
      <me:MCRCMethod name="SimpleRRKM"/>
      <me:transitionState>
        <molecule ref="TS2" me:type="transitionState" />
      </me:transitionState>
      <me:tunneling name="Eckart"/>
      <me:MCRCMethod name="SimpleRRKM"/>
    </reaction>
    <reaction id="R2">
      <reactant>
        <molecule ref="PC2" me:type="modelled" />
      </reactant>
      <product>
        <molecule ref="P2" me:type="sink" />
      </product>
      <product>
        <molecule ref="H2O" me:type="sink" />
      </product>
      <me:MCRCMethod name="MesmerILT" xsi:type="MesmerILT" >
        <me:preExponential units="cm3molecule-1s-1">1.00E-11</me:preExponential>
        <me:activationEnergy units="kcal/mol" reverse="true">0.01</me:activationEnergy>
    </me:MCRCMethod>
    </reaction>
  </reactionList>
  <me:conditions>
    <me:bathGas>N2</me:bathGas>
    <me:PTs>
      <me:PTpair me:units="Torr" me:P="760" me:T="200" me:precision= "qd" />
      <me:PTpair me:units="Torr" me:P="760" me:T="220" me:precision= "qd" />
```

```
      <me:PTpair me:units="Torr" me:P="760" me:T="240" me:precision= "qd" />
      <me:PTpair me:units="Torr" me:P="760" me:T="250" me:precision= "qd" />
      <me:PTpair me:units="Torr" me:P="760" me:T="260" me:precision= "qd" />
      <me:PTpair me:units="Torr" me:P="760" me:T="280" me:precision= "qd" />
      <me:PTpair me:units="Torr" me:P="760" me:T="298" me:precision= "qd" />
      <me:PTpair me:units="Torr" me:P="760" me:T="300" me:precision= "qd" />
      <me:PTpair me:units="Torr" me:P="760" me:T="320" me:precision= "qd" />
    </me:PTs>
    <me:InitialPopulation>
      <molecule ref="OH" me:population="1.0"/>
    </me:InitialPopulation>
  </me:conditions>
  <me:modelParameters>
    <me:grainSize units="cm-1">20</me:grainSize>
    <me:energyAboveTheTopHill>25</me:energyAboveTheTopHill>
  </me:modelParameters>
  <me:control>
    <me:eigenvalues>1</me:eigenvalues>
    <me:calcMethod>simpleCalc</me:calcMethod>
    <me:testMicroRates/>
    <me:testRateConstants/>
    <me:printGrainDOS/>
    <me:printGrainkfE/>
    <me:testDOS/>
    <me:printSpeciesProfile/>
    <me:printGrainedSpeciesProfile/>
  </me:control>
</me:mesmer>
```


Mesmer script for TS3


```
<?xml version="1.0" encoding="utf-8" ?>
<?xml-stylesheet type='text/xsl' href='../../mesmer2.xsl' media='other'?>
<?xml-stylesheet type='text/xsl' href='../../mesmer1.xsl' media='screen'?>
<me:mesmer xmlns="http://www.xml-cml.org/schema"
xmlns:me="http://www.chem.leeds.ac.uk/mesmer"
xmlns:xsi="http://www.w3.org/2001/XMLSchema-instance" xmlns:cml="http://www.xml-
cml.org/schema">
<me:title>321MBT_OH_TS3</me:title>
<moleculeList>
  <molecule id="321MBT">
    <propertyList>
      <property dictRef="me:ZPE">
        <scalar units="kcal/mol">0.0</scalar>
      </property>
```

<property dictRef="me:rotConsts">
<array units="cm-1">0.19625 0.04349 0.04035</array>
</property>
<property dictRef="me:vibFreqs">
<array units="cm-1">40.2728 115.9719 137.1856 199.4724 238.1814 316.9590 347.5624 402.8403 474.4488 519.2721 704.7871 770.9793 839.5900 887.5999 966.7843 1007.2573 1014.6785 1046.3552 1106.3000 1137.0336 1180.0570 1262.0525 1270.0480 1375.0776 1414.1651 1421.7355 1473.8347 1477.9148 1492.9447 1495.8890 1504.7109 1764.7289 2730.2078 3047.3869 3054.1175 3097.3687 3097.8866 3100.6151 3147.0004 3151.3191 3164.8649 3173.6496</array>
</property>
<property dictRef="me:frequenciesScaleFactor">
<scalar>1.0</scalar>
</property>
<property dictRef="me:symmetryNumber">
<scalar>1</scalar>
</property>
<property dictRef="me:MW">
<scalar units="amu">102.05032</scalar>
</property>
<property dictRef="me:spinMultiplicity">
<scalar>1</scalar>
</property>
</propertyList>
<me:DOSCMethod name="ClassicalRotors"/>
</molecule>
<molecule id="OH">
<propertyList>
<property dictRef="me:ZPE">
<scalar units="kcal/mol">0.0</scalar>
</property>
<property dictRef="me:rotConsts">
<array units="cm-1">18.83025</array>
</property>
<property dictRef="me:vibFreqs">
<array units="cm-1">3768.5403</array>
</property>
<property dictRef="me:frequenciesScaleFactor">
<scalar>1.0</scalar>
</property>
<property dictRef="me:symmetryNumber">
<scalar>1</scalar>
</property>
<property dictRef="me:MW">
<scalar units="amu">17.00274</scalar>
</property>

<property dictRef="me:spinMultiplicity">
<scalar>2</scalar>
</property>
</propertyList>
<me:DOSCMethod name="ClassicalRotors"/>
</molecule>
<molecule id="RC2">
<propertyList>
<property dictRef="me:ZPE">
<scalar units="kcal/mol">-3.6</scalar>
</property>
<property dictRef="me:rotConsts">
<array units="cm-1">0.08517 0.03711 0.02850</array>
</property>
<property dictRef="me:vibFreqs">
<array units="cm-1">30.9451 59.5534 76.3263 119.2061 138.7491 143.7835 194.2656 211.8785 321.4811 350.3024 366.8120 403.1757 420.7014 476.0277 526.3855 694.4637 769.5880 842.5191 888.7272 969.1120 1002.7876 1012.6180 1043.9410 1107.5784 1135.2397 1185.8401 1263.2303 1278.1043 1376.2153 1415.4743 1424.7868 1473.0708 1477.4287 1491.0373 1496.6969 1508.3204 1760.6348 2722.9847 3048.8728 3054.3520 3096.4679 3099.9312 3102.7838 3139.8262 3146.6642 3160.5663 3170.6882 3636.4704</array>
</property>
<property dictRef="me:frequenciesScaleFactor">
<scalar>1.0</scalar>
</property>
<property dictRef="me:symmetryNumber">
<scalar>1</scalar>
</property>
<property dictRef="me:MW">
<scalar units="amu">119.05306</scalar>
</property>
<property dictRef="me:spinMultiplicity">
<scalar>2</scalar>
</property>
<property dictRef="me:epsilon">
<scalar>306.5</scalar>
</property>
<property dictRef="me:sigma">
<scalar>4.42</scalar>
</property>
</propertyList>
<me:DOSCMethod name="ClassicalRotors"/>
<me:energyTransferModel xsi:type="me:ExponentialDown">
<me:deltaEDown units="cm-1">200</me:deltaEDown>
</me:energyTransferModel>

</molecule>
<molecule id="TS3">
<propertyList>
<property dictRef="me:ZPE">
<scalar units="kcal/mol">-0.6</scalar>
</property>
<property dictRef="me:rotConsts">
<array units="cm-1">0.08346 0.04007 0.03267</array>
</property>
<property dictRef="me:vibFreqs">
<array units="cm-1">54.0947 83.2553 118.3007 125.6138 157.5346 205.2766
227.4529 303.4782 333.6271 364.4011 400.6841 458.4511 527.7319 642.7992 708.3367
783.7738 839.0881 885.7854 969.6718 995.9431 1003.8838 1018.1515 1070.8042 1106.9919
1139.3129 1233.9186 1293.3450 1354.9366 1370.8811 1415.2991 1423.8579 1474.3771
1487.3076 1493.8521 1500.9895 1702.6804 1750.9828 2730.1104 3047.7597 3051.3324
3098.7995 3101.7639 3131.2167 3148.1992 3166.3075 3167.6935 3758.9747</array>
</property>
<property dictRef="me:frequenciesScaleFactor">
<scalar>1.0</scalar>
</property>
<property dictRef="me:symmetryNumber">
<scalar>1</scalar>
</property>
<property dictRef="me:spinMultiplicity">
<scalar>2</scalar>
</property>
<property dictRef="me:imFreqs">
<array units="cm-1">712.6057</array>
</property>
</propertyList>
<me:DOSCMethod name="ClassicalRotors"/>
</molecule>
<molecule id="PC3">
<propertyList>
<property dictRef="me:ZPE">
<scalar units="kcal/mol">-35.0</scalar>
</property>
<property dictRef="me:rotConsts">
<array units="cm-1">0.08132 0.04265 0.03667</array>
</property>
<property dictRef="me:vibFreqs">
<array units="cm-1">55.4689 80.4249 105.5655 130.8053 160.0274 174.2451
185.2565 195.6544 228.5039 284.2631 313.6031 382.4919 399.0561 439.3943 479.5350
642.7475 670.8147 721.2254 831.9396 901.8402 973.3853 994.3111 1004.1593 1046.6868
1079.3530 1187.7562 1240.6789 1349.8699 1394.5517 1411.7850 1428.1242 1467.6537
1478.5038 1487.5133 1504.8049 1530.1162 1614.4298 2724.2542 3031.7968 3039.4596

3076.0297 3100.5408 3139.1144 3147.3579 3152.9604 3209.9383 3807.1956
3930.5755</array>
</property>
<property dictRef="me:frequenciesScaleFactor">
<scalar>1.0</scalar>
</property>
<property dictRef="me:symmetryNumber">
<scalar>1</scalar>
</property>
<property dictRef="me:MW">
<scalar units="amu">119.05306</scalar>
</property>
<property dictRef="me:spinMultiplicity">
<scalar>2</scalar>
</property>
<property dictRef="me:epsilon">
<scalar>306.5</scalar>
</property>
<property dictRef="me:sigma">
<scalar>4.42</scalar>
</property>
</propertyList>
<me:DOSCMethod name="ClassicalRotors"/>
<me:energyTransferModel xsi:type="me:ExponentialDown">
<me:deltaEDown units="cm-1">200</me:deltaEDown>
</me:energyTransferModel>
</molecule>
<molecule id="P2">
<propertyList>
<property dictRef="me:ZPE">
<scalar units="kcal/mol">-34.3</scalar>
</property>
<property dictRef="me:rotConsts">
<array units="cm-1">0.25302 0.04002 0.03500</array>
</property>
<property dictRef="me:vibFreqs">
<array units="cm-1">85.9610 131.5048 167.2374 183.4367 239.9712 296.4347
301.4346 414.9136 416.4955 487.9792 707.6603 826.1410 827.4161 908.9132 982.6487
986.0236 997.9192 1051.9220 1071.8936 1183.2122 1233.6460 1320.0262 1399.3124
1413.0270 1429.8451 1472.6476 1477.2247 1487.1721 1498.3179 1518.7724 2731.6411
3029.0204 3035.3446 3070.8564 3080.9120 3135.1767 3137.6291 3153.3105
3215.6986</array>
</property>
<property dictRef="me:frequenciesScaleFactor">
<scalar>1</scalar>
</property>

```
      <property dictRef="me:symmetryNumber">
        <scalar>1</scalar>
      </property>
      <property dictRef="me:MW">
        <scalar units="amu">101.04250</scalar>
      </property>
      <property dictRef="me:spinMultiplicity">
        <scalar>2</scalar>
      </property>
    </propertyList>
    <me:DOSCMethod name="ClassicalRotors"/>
  </molecule>
  <molecule id="H2O">
    <propertyList>
      <property dictRef="me:ZPE">
        <scalar units="kcal/mol">0.0</scalar>
      </property>
      <property dictRef="me:rotConsts">
        <array units="cm-1">27.82574 14.37796 9.47967</array>
      </property>
      <property dictRef="me:vibFreqs">
        <array units="cm-1">1618.8285 3867.7498 3971.3807</array>
      </property>
      <property dictRef="me:frequenciesScaleFactor">
       <scalar>1.0</scalar>
      </property>
      <property dictRef="me:symmetryNumber">
        <scalar>1</scalar>
      </property>
      <property dictRef="me:MW">
        <scalar units="amu">18.01056</scalar>
      </property>
      <property dictRef="me:spinMultiplicity">
        <scalar>1</scalar>
      </property>
    </propertyList>
    <me:DOSCMethod name="ClassicalRotors"/>
  </molecule>
  <molecule id="N2">
    <propertyList>
      <property dictRef="me:MW">
        <scalar units="amu">28</scalar>
      </property>
      <property dictRef="me:epsilon">
        <scalar>48</scalar>
      </property>
```

```
      <property dictRef="me:sigma">
        <scalar>3.9</scalar>
      </property>
    </propertyList>
    <me:DOSCMethod name="ClassicalRotors"/>
  </molecule>
</moleculeList>
<reactionList>
  <reaction id="R0">
    <reactant>
      <molecule ref="OH" me:type="deficientReactant" />
    </reactant>
    <reactant>
      <molecule ref="321MBT" me:type="excessReactant" />
    </reactant>
    <product>
      <molecule ref="RC2" me:type="modelled" />
    </product>
    <me:excessReactantConc>1.00E11</me:excessReactantConc>
    <me:MCRCMethod name="MesmerILT"/>
    <me:preExponential units="cm3molecule-1s-1">1.00E-11</me:preExponential>
    <me:activationEnergy units="kcal/mol">0.01</me:activationEnergy>
    <me:TInfinity>298.0</me:TInfinity>
    <me:nInfinity>0.1</me:nInfinity>
  </reaction>
  <reaction id="R1">
    <reactant>
      <molecule ref="RC2" me:type="modelled" />
    </reactant>
    <product>
      <molecule ref="PC3" me:type="modelled" />
    </product>
    <me:MCRCMethod name="SimpleRRKM"/>
    <me:transitionState>
      <molecule ref="TS3" me:type="transitionState" />
    </me:transitionState>
    <me:tunneling name="Eckart"/>
    <me:MCRCMethod name="SimpleRRKM"/>
  </reaction>
  <reaction id="R2">
    <reactant>
      <molecule ref="PC3" me:type="modelled" />
    </reactant>
    <product>
      <molecule ref="P2" me:type="sink" />
    </product>
```

```xml
      <product>
        <molecule ref="H2O" me:type="sink" />
      </product>
      <me:MCRCMethod name="MesmerILT" xsi:type="MesmerILT" >
        <me:preExponential units="cm3molecule-1s-1">1.00E-11</me:preExponential>
        <me:activationEnergy units="kcal/mol" reverse="true">0.01</me:activationEnergy>
    </me:MCRCMethod>
    </reaction>
</reactionList>
<me:conditions>
    <me:bathGas>N2</me:bathGas>
    <me:PTs>
      <me:PTpair me:units="Torr" me:P="760" me:T="200" me:precision= "qd" />
      <me:PTpair me:units="Torr" me:P="760" me:T="220" me:precision= "qd" />
      <me:PTpair me:units="Torr" me:P="760" me:T="240" me:precision= "qd" />
      <me:PTpair me:units="Torr" me:P="760" me:T="250" me:precision= "qd" />
      <me:PTpair me:units="Torr" me:P="760" me:T="260" me:precision= "qd" />
      <me:PTpair me:units="Torr" me:P="760" me:T="280" me:precision= "qd" />
      <me:PTpair me:units="Torr" me:P="760" me:T="298" me:precision= "qd" />
      <me:PTpair me:units="Torr" me:P="760" me:T="300" me:precision= "qd" />
      <me:PTpair me:units="Torr" me:P="760" me:T="320" me:precision= "qd" />
    </me:PTs>
    <me:InitialPopulation>
      <molecule ref="OH" me:population="1.0"/>
    </me:InitialPopulation>
</me:conditions>
<me:modelParameters>
    <me:grainSize units="cm-1">20</me:grainSize>
    <me:energyAboveTheTopHill>25</me:energyAboveTheTopHill>
</me:modelParameters>
<me:control>
    <me:eigenvalues>1</me:eigenvalues>
    <me:calcMethod>simpleCalc</me:calcMethod>
    <me:testMicroRates/>
    <me:testRateConstants/>
    <me:printGrainDOS/>
    <me:printGrainkfE/>
    <me:testDOS/>
    <me:printSpeciesProfile/>
    <me:printGrainedSpeciesProfile/>
</me:control>
</me:mesmer>
```


Mesmer script for TS4

```
<?xml version="1.0" encoding="utf-8" ?>
<?xml-stylesheet type='text/xsl' href='../../mesmer2.xsl' media='other'?>
<?xml-stylesheet type='text/xsl' href='../../mesmer1.xsl' media='screen'?>
<me:mesmer xmlns="http://www.xml-cml.org/schema"
xmlns:me="http://www.chem.leeds.ac.uk/mesmer"
xmlns:xsi="http://www.w3.org/2001/XMLSchema-instance" xmlns:cml="http://www.xml-
cml.org/schema">
<me:title>321MBT_OH_TS4</me:title>
<moleculeList>
  <molecule id="321MBT">
    <propertyList>
      <property dictRef="me:ZPE">
        <scalar units="kcal/mol">0.0</scalar>
      </property>
      <property dictRef="me:rotConsts">
        <array units="cm-1">0.19625 0.04349 0.04035</array>
      </property>
      <property dictRef="me:vibFreqs">
        <array units="cm-1">40.2728 115.9719 137.1856 199.4724 238.1814 316.9590
347.5624 402.8403 474.4488 519.2721 704.7871 770.9793 839.5900 887.5999 966.7843
1007.2573 1014.6785 1046.3552 1106.3000 1137.0336 1180.0570 1262.0525 1270.0480
1375.0776 1414.1651 1421.7355 1473.8347 1477.9148 1492.9447 1495.8890 1504.7109
1764.7289 2730.2078 3047.3869 3054.1175 3097.3687 3097.8866 3100.6151 3147.0004
3151.3191 3164.8649 3173.6496</array>
      </property>
      <property dictRef="me:frequenciesScaleFactor">
       <scalar>1.0</scalar>
      </property>
      <property dictRef="me:symmetryNumber">
        <scalar>1</scalar>
      </property>
      <property dictRef="me:MW">
        <scalar units="amu">102.05032</scalar>
      </property>
      <property dictRef="me:spinMultiplicity">
        <scalar>1</scalar>
      </property>
    </propertyList>
    <me:DOSCMethod name="ClassicalRotors"/>
  </molecule>
  <molecule id="OH">
    <propertyList>
      <property dictRef="me:ZPE">
        <scalar units="kcal/mol">0.0</scalar>
      </property>
      <property dictRef="me:rotConsts">
```

<array units="cm-1">18.83025</array>
</property>
<property dictRef="me:vibFreqs">
<array units="cm-1">3768.5403</array>
</property>
<property dictRef="me:frequenciesScaleFactor">
<scalar>1.0</scalar>
</property>
<property dictRef="me:symmetryNumber">
<scalar>1</scalar>
</property>
<property dictRef="me:MW">
<scalar units="amu">17.00274</scalar>
</property>
<property dictRef="me:spinMultiplicity">
<scalar>2</scalar>
</property>
</propertyList>
<me:DOSCMethod name="ClassicalRotors"/>
</molecule>
<molecule id="RC4">
<propertyList>
<property dictRef="me:ZPE">
<scalar units="kcal/mol">-4.2</scalar>
</property>
<property dictRef="me:rotConsts">
<array units="cm-1">0.09554 0.04198 0.03662</array>
</property>
<property dictRef="me:vibFreqs">
<array units="cm-1">35.7787 70.3183 124.2449 134.5829 149.0950 166.9956
212.3151 246.2130 313.5306 352.5367 398.8715 441.4619 471.5616 497.1640 542.3588
677.0556 761.2820 829.7183 883.5424 969.8058 993.5754 1007.5748 1048.6448 1105.4685
1135.4445 1189.7518 1261.3261 1280.1405 1370.7399 1416.1810 1417.3408 1472.0102
1475.0698 1490.8198 1494.6929 1503.2930 1741.5790 2717.6292 3052.9388 3056.2290
3106.6369 3107.3552 3112.5705 3148.3617 3154.0865 3156.7024 3174.6065
3694.8463</array>
</property>
<property dictRef="me:frequenciesScaleFactor">
<scalar>1.0</scalar>
</property>
<property dictRef="me:symmetryNumber">
<scalar>1</scalar>
</property>
<property dictRef="me:MW">
<scalar units="amu">119.05306</scalar>
</property>

<property dictRef="me:spinMultiplicity">
<scalar>2</scalar>
</property>
<property dictRef="me:epsilon">
<scalar>306.5</scalar>
</property>
<property dictRef="me:sigma">
<scalar>4.42</scalar>
</property>
</propertyList>
<me:DOSCMethod name="ClassicalRotors"/>
<me:energyTransferModel xsi:type="me:ExponentialDown">
<me:deltaEDown units="cm-1">200</me:deltaEDown>
</me:energyTransferModel>
</molecule>
<molecule id="TS4">
<propertyList>
<property dictRef="me:ZPE">
<scalar units="kcal/mol">1.0</scalar>
</property>
<property dictRef="me:rotConsts">
<array units="cm-1">0.08196 0.04176 0.03030</array>
</property>
<property dictRef="me:vibFreqs">
<array units="cm-1">61.6182 85.2881 112.7779 119.7991 143.5582 200.9902
242.8835 285.9543 336.4634 359.2971 385.9420 421.8287 512.0487 570.9763 718.7670
767.4449 846.1386 918.9615 954.0719 970.6884 999.0001 1028.0538 1073.0507 1104.4189
1158.2480 1210.8840 1254.7939 1270.1725 1403.1304 1416.2523 1432.7579 1461.8221
1474.9686 1487.9694 1495.4168 1497.7200 1765.5753 2730.8391 3051.0688 3056.5845
3090.4073 3103.6427 3107.7840 3140.3700 3159.4925 3164.3095 3751.1729</array>
</property>
<property dictRef="me:frequenciesScaleFactor">
<scalar>1.0</scalar>
</property>
<property dictRef="me:symmetryNumber">
<scalar>1</scalar>
</property>
<property dictRef="me:spinMultiplicity">
<scalar>2</scalar>
</property>
<property dictRef="me:imFreqs">
<array units="cm-1">1050.6351</array>
</property>
</propertyList>
<me:DOSCMethod name="ClassicalRotors"/>
</molecule>

<molecule id="PC4">
<propertyList>
<property dictRef="me:ZPE">
<scalar units="kcal/mol">-10.8</scalar>
</property>
<property dictRef="me:rotConsts">
<array units="cm-1">0.07681 0.03713 0.02754</array>
</property>
<property dictRef="me:vibFreqs">
<array units="cm-1">22.4349 44.8066 80.9505 113.8549 130.3917 157.3695
197.2901 201.0511 216.9150 258.5526 294.1073 329.1240 385.6252 435.1540 484.9063
500.8854 694.9506 758.5933 827.7770 960.0182 962.2941 992.0986 1002.7179 1096.8418
1160.4741 1180.6012 1227.1721 1274.1628 1394.9922 1410.1872 1450.1471 1473.8863
1484.5462 1492.4252 1494.9803 1632.2453 1792.1724 2726.7728 3051.4333 3052.2792
3055.7834 3106.6732 3109.8471 3115.1144 3153.7505 3154.2609 3817.1806
3908.1584</array>
</property>
<property dictRef="me:frequenciesScaleFactor">
<scalar>1.0</scalar>
</property>
<property dictRef="me:symmetryNumber">
<scalar>1</scalar>
</property>
<property dictRef="me:MW">
<scalar units="amu">119.05306</scalar>
</property>
<property dictRef="me:spinMultiplicity">
<scalar>2</scalar>
</property>
<property dictRef="me:epsilon">
<scalar>306.5</scalar>
</property>
<property dictRef="me:sigma">
<scalar>4.42</scalar>
</property>
</propertyList>
<me:DOSCMethod name="ClassicalRotors"/>
<me:energyTransferModel xsi:type="me:ExponentialDown">
<me:deltaEDown units="cm-1">200</me:deltaEDown>
</me:energyTransferModel>
</molecule>
<molecule id="P3">
<propertyList>
<property dictRef="me:ZPE">
<scalar units="kcal/mol">-7.8</scalar>
</property>

<property dictRef="me:rotConsts">
<array units="cm-1">0.20236 0.04214 0.03913</array>
</property>
<property dictRef="me:vibFreqs">
<array units="cm-1">22.1968 106.7002 157.5756 165.4530 202.2801 236.2518 325.7176 383.5624 481.9971 499.3667 701.2744 759.9462 824.6916 960.9838 961.6871 1000.1917 1007.2531 1096.8221 1161.5167 1180.3116 1226.8022 1268.1354 1393.5657 1408.3855 1454.0446 1474.7789 1484.2937 1493.1533 1494.9373 1792.2713 2730.7672 3046.1017 3048.9892 3052.9036 3102.3538 3106.1425 3109.7860 3150.4080 3156.3167</array>
</property>
<property dictRef="me:frequenciesScaleFactor">
<scalar>1</scalar>
</property>
<property dictRef="me:symmetryNumber">
<scalar>1</scalar>
</property>
<property dictRef="me:MW">
<scalar units="amu">101.04250</scalar>
</property>
<property dictRef="me:spinMultiplicity">
<scalar>2</scalar>
</property>
</propertyList>
<me:DOSCMethod name="ClassicalRotors"/>
</molecule>
<molecule id="H2O">
<propertyList>
<property dictRef="me:ZPE">
<scalar units="kcal/mol">0.0</scalar>
</property>
<property dictRef="me:rotConsts">
<array units="cm-1">27.82574 14.37796 9.47967</array>
</property>
<property dictRef="me:vibFreqs">
<array units="cm-1">1618.8285 3867.7498 3971.3807</array>
</property>
<property dictRef="me:frequenciesScaleFactor">
<scalar>1.0</scalar>
</property>
<property dictRef="me:symmetryNumber">
<scalar>1</scalar>
</property>
<property dictRef="me:MW">
<scalar units="amu">18.01056</scalar>
</property>

```
        <property dictRef="me:spinMultiplicity">
          <scalar>1</scalar>
        </property>
      </propertyList>
      <me:DOSCMethod name="ClassicalRotors"/>
    </molecule>
    <molecule id="N2">
      <propertyList>
        <property dictRef="me:MW">
          <scalar units="amu">28</scalar>
        </property>
        <property dictRef="me:epsilon">
          <scalar>48</scalar>
        </property>
        <property dictRef="me:sigma">
          <scalar>3.9</scalar>
        </property>
      </propertyList>
      <me:DOSCMethod name="ClassicalRotors"/>
    </molecule>
  </moleculeList>
  <reactionList>
    <reaction id="R0">
      <reactant>
        <molecule ref="OH" me:type="deficientReactant" />
      </reactant>
      <reactant>
        <molecule ref="321MBT" me:type="excessReactant" />
      </reactant>
      <product>
        <molecule ref="RC4" me:type="modelled" />
      </product>
      <me:excessReactantConc>1.00E11</me:excessReactantConc>
      <me:MCRCMethod name="MesmerILT"/>
      <me:preExponential units="cm3molecule-1s-1">1.00E-11</me:preExponential>
      <me:activationEnergy units="kcal/mol">0.01</me:activationEnergy>
      <me:TInfinity>298.0</me:TInfinity>
      <me:nInfinity>0.1</me:nInfinity>
    </reaction>
    <reaction id="R1">
      <reactant>
        <molecule ref="RC4" me:type="modelled" />
      </reactant>
      <product>
        <molecule ref="PC4" me:type="modelled" />
      </product>
```

```
      <me:MCRCMethod name="SimpleRRKM"/>
      <me:transitionState>
        <molecule ref="TS4" me:type="transitionState" />
      </me:transitionState>
      <me:tunneling name="Eckart"/>
      <me:MCRCMethod name="SimpleRRKM"/>
    </reaction>
    <reaction id="R2">
      <reactant>
        <molecule ref="PC4" me:type="modelled" />
      </reactant>
      <product>
        <molecule ref="P3" me:type="sink" />
      </product>
      <product>
        <molecule ref="H2O" me:type="sink" />
      </product>
      <me:MCRCMethod name="MesmerILT" xsi:type="MesmerILT" >
        <me:preExponential units="cm3molecule-1s-1">1.00E-11</me:preExponential>
        <me:activationEnergy units="kcal/mol" reverse="true">0.01</me:activationEnergy>
    </me:MCRCMethod>
    </reaction>
  </reactionList>
  <me:conditions>
    <me:bathGas>N2</me:bathGas>
    <me:PTs>
      <me:PTpair me:units="Torr" me:P="760" me:T="200" me:precision= "qd" />
      <me:PTpair me:units="Torr" me:P="760" me:T="220" me:precision= "qd" />
      <me:PTpair me:units="Torr" me:P="760" me:T="240" me:precision= "qd" />
      <me:PTpair me:units="Torr" me:P="760" me:T="250" me:precision= "qd" />
      <me:PTpair me:units="Torr" me:P="760" me:T="260" me:precision= "qd" />
      <me:PTpair me:units="Torr" me:P="760" me:T="280" me:precision= "qd" />
      <me:PTpair me:units="Torr" me:P="760" me:T="298" me:precision= "qd" />
      <me:PTpair me:units="Torr" me:P="760" me:T="300" me:precision= "qd" />
      <me:PTpair me:units="Torr" me:P="760" me:T="320" me:precision= "qd" />
    </me:PTs>
    <me:InitialPopulation>
      <molecule ref="OH" me:population="1.0"/>
    </me:InitialPopulation>
  </me:conditions>
  <me:modelParameters>
    <me:grainSize units="cm-1">20</me:grainSize>
    <me:energyAboveTheTopHill>25</me:energyAboveTheTopHill>
  </me:modelParameters>
  <me:control>
    <me:eigenvalues>1</me:eigenvalues>
```

```
    <me:calcMethod>simpleCalc</me:calcMethod>
    <me:testMicroRates/>
    <me:testRateConstants/>
    <me:printGrainDOS/>
    <me:printGrainkfE/>
    <me:testDOS/>
    <me:printSpeciesProfile/>
    <me:printGrainedSpeciesProfile/>
</me:control>
</me:mesmer>
```

<u>Mesmer script for TS5</u>

```
<?xml version="1.0" encoding="utf-8" ?>
<?xml-stylesheet type='text/xsl' href='../../mesmer2.xsl' media='other'?>
<?xml-stylesheet type='text/xsl' href='../../mesmer1.xsl' media='screen'?>
<me:mesmer xmlns="http://www.xml-cml.org/schema"
xmlns:me="http://www.chem.leeds.ac.uk/mesmer"
xmlns:xsi="http://www.w3.org/2001/XMLSchema-instance" xmlns:cml="http://www.xml-
cml.org/schema">
<me:title>321MBT_OH_TS5</me:title>
<moleculeList>
    <molecule id="321MBT">
        <propertyList>
            <property dictRef="me:ZPE">
                <scalar units="kcal/mol">0.0</scalar>
            </property>
            <property dictRef="me:rotConsts">
                <array units="cm-1">0.19625 0.04349 0.04035</array>
            </property>
            <property dictRef="me:vibFreqs">
                <array units="cm-1">40.2728 115.9719 137.1856 199.4724 238.1814 316.9590
347.5624 402.8403 474.4488 519.2721 704.7871 770.9793 839.5900 887.5999 966.7843
1007.2573 1014.6785 1046.3552 1106.3000 1137.0336 1180.0570 1262.0525 1270.0480
1375.0776 1414.1651 1421.7355 1473.8347 1477.9148 1492.9447 1495.8890 1504.7109
1764.7289 2730.2078 3047.3869 3054.1175 3097.3687 3097.8866 3100.6151 3147.0004
3151.3191 3164.8649 3173.6496</array>
            </property>
            <property dictRef="me:frequenciesScaleFactor">
               <scalar>1.0</scalar>
            </property>
            <property dictRef="me:symmetryNumber">
               <scalar>1</scalar>
            </property>
            <property dictRef="me:MW">
```

<scalar units="amu">102.05032</scalar>
</property>
<property dictRef="me:spinMultiplicity">
<scalar>1</scalar>
</property>
</propertyList>
<me:DOSCMethod name="ClassicalRotors"/>
</molecule>
<molecule id="OH">
<propertyList>
<property dictRef="me:ZPE">
<scalar units="kcal/mol">0.0</scalar>
</property>
<property dictRef="me:rotConsts">
<array units="cm-1">18.83025</array>
</property>
<property dictRef="me:vibFreqs">
<array units="cm-1">3768.5403</array>
</property>
<property dictRef="me:frequenciesScaleFactor">
<scalar>1.0</scalar>
</property>
<property dictRef="me:symmetryNumber">
<scalar>1</scalar>
</property>
<property dictRef="me:MW">
<scalar units="amu">17.00274</scalar>
</property>
<property dictRef="me:spinMultiplicity">
<scalar>2</scalar>
</property>
</propertyList>
<me:DOSCMethod name="ClassicalRotors"/>
</molecule>
<molecule id="RC4">
<propertyList>
<property dictRef="me:ZPE">
<scalar units="kcal/mol">-4.2</scalar>
</property>
<property dictRef="me:rotConsts">
<array units="cm-1">0.09554 0.04198 0.03662</array>
</property>
<property dictRef="me:vibFreqs">
<array units="cm-1">35.7787 70.3183 124.2449 134.5829 149.0950 166.9956
212.3151 246.2130 313.5306 352.5367 398.8715 441.4619 471.5616 497.1640 542.3588
677.0556 761.2820 829.7183 883.5424 969.8058 993.5754 1007.5748 1048.6448 1105.4685

1135.4445 1189.7518 1261.3261 1280.1405 1370.7399 1416.1810 1417.3408 1472.0102
1475.0698 1490.8198 1494.6929 1503.2930 1741.5790 2717.6292 3052.9388 3056.2290
3106.6369 3107.3552 3112.5705 3148.3617 3154.0865 3156.7024 3174.6065
3694.8463</array>
</property>
<property dictRef="me:frequenciesScaleFactor">
<scalar>1.0</scalar>
</property>
<property dictRef="me:symmetryNumber">
<scalar>1</scalar>
</property>
<property dictRef="me:MW">
<scalar units="amu">119.05306</scalar>
</property>
<property dictRef="me:spinMultiplicity">
<scalar>2</scalar>
</property>
<property dictRef="me:epsilon">
<scalar>306.5</scalar>
</property>
<property dictRef="me:sigma">
<scalar>4.42</scalar>
</property>
</propertyList>
<me:DOSCMethod name="ClassicalRotors"/>
<me:energyTransferModel xsi:type="me:ExponentialDown">
<me:deltaEDown units="cm-1">200</me:deltaEDown>
</me:energyTransferModel>
</molecule>
<molecule id="TS5">
<propertyList>
<property dictRef="me:ZPE">
<scalar units="kcal/mol">0.1</scalar>
</property>
<property dictRef="me:rotConsts">
<array units="cm-1">0.07149 0.04513 0.03212</array>
</property>
<property dictRef="me:vibFreqs">
<array units="cm-1">28.6170 61.8380 69.6561 85.1467 126.6149 200.0631 242.5912
303.8256 326.4587 350.9916 404.3258 469.5836 530.3024 659.9723 700.6092 780.0433
850.4146 890.7515 986.1829 989.3063 1015.0759 1048.4634 1088.7973 1133.7156 1174.5231
1256.0586 1268.8694 1297.1101 1346.3755 1380.1763 1415.8177 1469.7101 1484.6887
1496.6332 1503.1557 1651.9180 1834.2824 2698.7971 3049.9313 3098.7108 3102.8794
3106.6524 3148.8284 3151.1037 3169.9836 3178.3116 3777.1637</array>
</property>
<property dictRef="me:frequenciesScaleFactor">

<scalar>1.0</scalar>
</property>
<property dictRef="me:symmetryNumber">
<scalar>1</scalar>
</property>
<property dictRef="me:spinMultiplicity">
<scalar>2</scalar>
</property>
<property dictRef="me:imFreqs">
<array units="cm-1">672.5616</array>
</property>
</propertyList>
<me:DOSCMethod name="ClassicalRotors"/>
</molecule>
<molecule id="PC5">
<propertyList>
<property dictRef="me:ZPE">
<scalar units="kcal/mol">-32.8</scalar>
</property>
<property dictRef="me:rotConsts">
<array units="cm-1">0.07994 0.04041 0.03260</array>
</property>
<property dictRef="me:vibFreqs">
<array units="cm-1">13.5305 48.7653 77.6952 93.8775 123.0928 133.6944 149.4398 217.7222 282.1693 320.9817 368.6389 400.4006 432.9467 495.0649 510.1087 610.6688 696.8584 764.8488 778.0469 837.3752 891.8374 962.2114 1007.8148 1049.8337 1064.1513 1139.0611 1195.3793 1245.8069 1285.6929 1337.5492 1409.7436 1451.9716 1489.2094 1495.9265 1502.3226 1559.7554 1610.1095 2693.3733 3065.8029 3087.2562 3127.9529 3152.5663 3154.9492 3178.3625 3185.4555 3266.4262 3790.4952 3934.8556</array>
</property>
<property dictRef="me:frequenciesScaleFactor">
<scalar>1.0</scalar>
</property>
<property dictRef="me:symmetryNumber">
<scalar>1</scalar>
</property>
<property dictRef="me:MW">
<scalar units="amu">119.05306</scalar>
</property>
<property dictRef="me:spinMultiplicity">
<scalar>2</scalar>
</property>
<property dictRef="me:epsilon">
<scalar>306.5</scalar>
</property>
<property dictRef="me:sigma">

<scalar>4.42</scalar>
</property>
</propertyList>
<me:DOSCMethod name="ClassicalRotors"/>
<me:energyTransferModel xsi:type="me:ExponentialDown">
<me:deltaEDown units="cm-1">200</me:deltaEDown>
</me:energyTransferModel>
</molecule>
<molecule id="P4">
<propertyList>
<property dictRef="me:ZPE">
<scalar units="kcal/mol">-29.5</scalar>
</property>
<property dictRef="me:rotConsts">
<array units="cm-1">0.19971 0.04750 0.04455</array>
</property>
<property dictRef="me:vibFreqs">
<array units="cm-1">70.8637 84.4487 106.9806 173.0253 304.6887 318.7750 428.7644 484.8145 525.1031 603.7048 654.6832 737.5220 787.4276 852.8379 883.6111 953.9823 1004.5478 1053.8957 1064.6069 1128.7833 1198.6969 1253.3244 1278.7220 1334.8805 1410.6218 1452.6993 1489.3660 1497.6437 1505.8495 1566.0557 2722.9610 3067.1244 3104.7070 3126.7407 3152.6138 3153.1238 3175.3520 3191.6923 3263.6723</array>
</property>
<property dictRef="me:frequenciesScaleFactor">
<scalar>1</scalar>
</property>
<property dictRef="me:symmetryNumber">
<scalar>1</scalar>
</property>
<property dictRef="me:MW">
<scalar units="amu">101.04250</scalar>
</property>
<property dictRef="me:spinMultiplicity">
<scalar>2</scalar>
</property>
</propertyList>
<me:DOSCMethod name="ClassicalRotors"/>
</molecule>
<molecule id="H2O">
<propertyList>
<property dictRef="me:ZPE">
<scalar units="kcal/mol">0.0</scalar>
</property>
<property dictRef="me:rotConsts">
<array units="cm-1">27.82574 14.37796 9.47967</array>

```xml
        </property>
        <property dictRef="me:vibFreqs">
          <array units="cm-1">1618.8285 3867.7498 3971.3807</array>
        </property>
        <property dictRef="me:frequenciesScaleFactor">
          <scalar>1.0</scalar>
        </property>
        <property dictRef="me:symmetryNumber">
          <scalar>1</scalar>
        </property>
        <property dictRef="me:MW">
          <scalar units="amu">18.01056</scalar>
        </property>
        <property dictRef="me:spinMultiplicity">
          <scalar>1</scalar>
        </property>
      </propertyList>
      <me:DOSCMethod name="ClassicalRotors"/>
    </molecule>
    <molecule id="N2">
      <propertyList>
        <property dictRef="me:MW">
          <scalar units="amu">28</scalar>
        </property>
        <property dictRef="me:epsilon">
          <scalar>48</scalar>
        </property>
        <property dictRef="me:sigma">
          <scalar>3.9</scalar>
        </property>
      </propertyList>
      <me:DOSCMethod name="ClassicalRotors"/>
    </molecule>
  </moleculeList>
  <reactionList>
    <reaction id="R0">
      <reactant>
        <molecule ref="OH" me:type="deficientReactant" />
      </reactant>
      <reactant>
        <molecule ref="321MBT" me:type="excessReactant" />
      </reactant>
      <product>
        <molecule ref="RC4" me:type="modelled" />
      </product>
      <me:excessReactantConc>1.00E11</me:excessReactantConc>
```

```
      <me:MCRCMethod name="MesmerILT"/>
      <me:preExponential units="cm3molecule-1s-1">1.00E-11</me:preExponential>
      <me:activationEnergy units="kcal/mol">0.01</me:activationEnergy>
      <me:TInfinity>298.0</me:TInfinity>
      <me:nInfinity>0.1</me:nInfinity>
    </reaction>
    <reaction id="R1">
      <reactant>
        <molecule ref="RC4" me:type="modelled" />
      </reactant>
      <product>
        <molecule ref="PC5" me:type="modelled" />
      </product>
      <me:MCRCMethod name="SimpleRRKM"/>
      <me:transitionState>
        <molecule ref="TS5" me:type="transitionState" />
      </me:transitionState>
      <me:tunneling name="Eckart"/>
      <me:MCRCMethod name="SimpleRRKM"/>
    </reaction>
    <reaction id="R2">
      <reactant>
        <molecule ref="PC5" me:type="modelled" />
      </reactant>
      <product>
        <molecule ref="P4" me:type="sink" />
      </product>
      <product>
        <molecule ref="H2O" me:type="sink" />
      </product>
      <me:MCRCMethod name="MesmerILT" xsi:type="MesmerILT" >
        <me:preExponential units="cm3molecule-1s-1">1.00E-11</me:preExponential>
        <me:activationEnergy units="kcal/mol" reverse="true">0.01</me:activationEnergy>
    </me:MCRCMethod>
    </reaction>
  </reactionList>
  <me:conditions>
    <me:bathGas>N2</me:bathGas>
    <me:PTs>
      <me:PTpair me:units="Torr" me:P="760" me:T="200" me:precision= "qd" />
      <me:PTpair me:units="Torr" me:P="760" me:T="220" me:precision= "qd" />
      <me:PTpair me:units="Torr" me:P="760" me:T="240" me:precision= "qd" />
      <me:PTpair me:units="Torr" me:P="760" me:T="250" me:precision= "qd" />
      <me:PTpair me:units="Torr" me:P="760" me:T="260" me:precision= "qd" />
      <me:PTpair me:units="Torr" me:P="760" me:T="280" me:precision= "qd" />
      <me:PTpair me:units="Torr" me:P="760" me:T="298" me:precision= "qd" />
```

```
      <me:PTpair me:units="Torr" me:P="760" me:T="300" me:precision= "qd" />
      <me:PTpair me:units="Torr" me:P="760" me:T="320" me:precision= "qd" />
   </me:PTs>
   <me:InitialPopulation>
      <molecule ref="OH" me:population="1.0"/>
   </me:InitialPopulation>
</me:conditions>
<me:modelParameters>
   <me:grainSize units="cm-1">20</me:grainSize>
   <me:energyAboveTheTopHill>25</me:energyAboveTheTopHill>
</me:modelParameters>
<me:control>
   <me:eigenvalues>1</me:eigenvalues>
   <me:calcMethod>simpleCalc</me:calcMethod>
   <me:testMicroRates/>
   <me:testRateConstants/>
   <me:printGrainDOS/>
   <me:printGrainkfE/>
   <me:testDOS/>
   <me:printSpeciesProfile/>
   <me:printGrainedSpeciesProfile/>
</me:control>
</me:mesmer>
```

Mesmer script for TS6


```
<?xml version="1.0" encoding="utf-8" ?>
<?xml-stylesheet type='text/xsl' href='../../mesmer2.xsl' media='other'?>
<?xml-stylesheet type='text/xsl' href='../../mesmer1.xsl' media='screen'?>
<me:mesmer xmlns="http://www.xml-cml.org/schema"
xmlns:me="http://www.chem.leeds.ac.uk/mesmer"
xmlns:xsi="http://www.w3.org/2001/XMLSchema-instance" xmlns:cml="http://www.xml-
cml.org/schema">
<me:title>321MBT_OH_TS6</me:title>
<moleculeList>
   <molecule id="321MBT">
      <propertyList>
         <property dictRef="me:ZPE">
            <scalar units="kcal/mol">0.0</scalar>
         </property>
         <property dictRef="me:rotConsts">
            <array units="cm-1">0.19625 0.04349 0.04035</array>
         </property>
         <property dictRef="me:vibFreqs">
            <array units="cm-1">40.2728 115.9719 137.1856 199.4724 238.1814 316.9590
347.5624 402.8403 474.4488 519.2721 704.7871 770.9793 839.5900 887.5999 966.7843
```

```
1007.2573 1014.6785 1046.3552 1106.3000 1137.0336 1180.0570 1262.0525 1270.0480
1375.0776 1414.1651 1421.7355 1473.8347 1477.9148 1492.9447 1495.8890 1504.7109
1764.7289 2730.2078 3047.3869 3054.1175 3097.3687 3097.8866 3100.6151 3147.0004
3151.3191 3164.8649 3173.6496</array>
      </property>
      <property dictRef="me:frequenciesScaleFactor">
        <scalar>1.0</scalar>
      </property>
      <property dictRef="me:symmetryNumber">
        <scalar>1</scalar>
      </property>
      <property dictRef="me:MW">
        <scalar units="amu">102.05032</scalar>
      </property>
      <property dictRef="me:spinMultiplicity">
        <scalar>1</scalar>
      </property>
    </propertyList>
    <me:DOSCMethod name="ClassicalRotors"/>
  </molecule>
  <molecule id="OH">
    <propertyList>
      <property dictRef="me:ZPE">
        <scalar units="kcal/mol">0.0</scalar>
      </property>
      <property dictRef="me:rotConsts">
        <array units="cm-1">18.83025</array>
      </property>
      <property dictRef="me:vibFreqs">
        <array units="cm-1">3768.5403</array>
      </property>
      <property dictRef="me:frequenciesScaleFactor">
        <scalar>1.0</scalar>
      </property>
      <property dictRef="me:symmetryNumber">
        <scalar>1</scalar>
      </property>
      <property dictRef="me:MW">
        <scalar units="amu">17.00274</scalar>
      </property>
      <property dictRef="me:spinMultiplicity">
        <scalar>2</scalar>
      </property>
    </propertyList>
    <me:DOSCMethod name="ClassicalRotors"/>
  </molecule>
```

<molecule id="RC3">
<propertyList>
<property dictRef="me:ZPE">
<scalar units="kcal/mol">-4.2</scalar>
</property>
<property dictRef="me:rotConsts">
<array units="cm-1">0.09554 0.04198 0.03662</array>
</property>
<property dictRef="me:vibFreqs">
<array units="cm-1">35.7787 70.3183 124.2449 134.5829 149.0950 166.9956
212.3151 246.2130 313.5306 352.5367 398.8715 441.4619 471.5616 497.1640 542.3588
677.0556 761.2820 829.7183 883.5424 969.8058 993.5754 1007.5748 1048.6448 1105.4685
1135.4445 1189.7518 1261.3261 1280.1405 1370.7399 1416.1810 1417.3408 1472.0102
1475.0698 1490.8198 1494.6929 1503.2930 1741.5790 2717.6292 3052.9388 3056.2290
3106.6369 3107.3552 3112.5705 3148.3617 3154.0865 3156.7024 3174.6065
3694.8463</array>
</property>
<property dictRef="me:frequenciesScaleFactor">
<scalar>1.0</scalar>
</property>
<property dictRef="me:symmetryNumber">
<scalar>1</scalar>
</property>
<property dictRef="me:MW">
<scalar units="amu">119.05306</scalar>
</property>
<property dictRef="me:spinMultiplicity">
<scalar>2</scalar>
</property>
<property dictRef="me:epsilon">
<scalar>306.5</scalar>
</property>
<property dictRef="me:sigma">
<scalar>4.42</scalar>
</property>
</propertyList>
<me:DOSCMethod name="ClassicalRotors"/>
<me:energyTransferModel xsi:type="me:ExponentialDown">
<me:deltaEDown units="cm-1">200</me:deltaEDown>
</me:energyTransferModel>
</molecule>
<molecule id="TS6">
<propertyList>
<property dictRef="me:ZPE">
<scalar units="kcal/mol">0.9</scalar>
</property>

<property dictRef="me:rotConsts">
<array units="cm-1">0.10717 0.02936 0.02603</array>
</property>
<property dictRef="me:vibFreqs">
<array units="cm-1">25.7405 40.8974 76.8288 127.0523 170.2731 205.7722
275.2253 307.8860 334.8656 355.1619 399.6303 456.7797 518.8199 691.4698 724.3039
778.4553 847.7091 890.6651 927.3391 986.1267 1007.0235 1046.8732 1079.2518 1135.7936
1180.9345 1262.8779 1270.3855 1330.2329 1357.9731 1401.9276 1415.6109 1464.9566
1483.2003 1496.2669 1500.7625 1573.1114 1762.2473 2730.7254 3050.3192 3094.3751
3098.6697 3103.3654 3149.9910 3151.8375 3171.8320 3178.6494 3774.1384</array>
</property>
<property dictRef="me:frequenciesScaleFactor">
<scalar>1.0</scalar>
</property>
<property dictRef="me:symmetryNumber">
<scalar>1</scalar>
</property>
<property dictRef="me:spinMultiplicity">
<scalar>2</scalar>
</property>
<property dictRef="me:imFreqs">
<array units="cm-1">840.4725</array>
</property>
</propertyList>
<me:DOSCMethod name="ClassicalRotors"/>
</molecule>
<molecule id="PC6">
<propertyList>
<property dictRef="me:ZPE">
<scalar units="kcal/mol">-32.0</scalar>
</property>
<property dictRef="me:rotConsts">
<array units="cm-1">0.07002 0.04041 0.03059</array>
</property>
<property dictRef="me:vibFreqs">
<array units="cm-1">40.9478 57.6632 77.5579 89.6561 102.4130 125.3057 147.1574
193.6143 270.9335 299.2407 330.0015 332.3976 427.5682 492.3255 524.6881 621.4944
662.2332 745.8907 785.5782 857.2700 882.5822 954.4076 1005.3014 1053.6974 1064.7058
1130.3767 1205.4824 1251.9821 1284.1326 1344.2845 1409.5232 1453.0177 1488.9770
1497.3274 1506.2104 1565.3358 1614.6441 2729.6381 3066.6144 3113.6135 3126.8483
3152.2594 3162.6530 3172.8092 3193.8730 3261.1397 3820.2561 3942.5234</array>
</property>
<property dictRef="me:frequenciesScaleFactor">
<scalar>1.0</scalar>
</property>
<property dictRef="me:symmetryNumber">

<scalar>1</scalar>
</property>
<property dictRef="me:MW">
<scalar units="amu">119.05306</scalar>
</property>
<property dictRef="me:spinMultiplicity">
<scalar>2</scalar>
</property>
<property dictRef="me:epsilon">
<scalar>306.5</scalar>
</property>
<property dictRef="me:sigma">
<scalar>4.42</scalar>
</property>
</propertyList>
<me:DOSCMethod name="ClassicalRotors"/>
<me:energyTransferModel xsi:type="me:ExponentialDown">
<me:deltaEDown units="cm-1">200</me:deltaEDown>
</me:energyTransferModel>
</molecule>
<molecule id="P4">
<propertyList>
<property dictRef="me:ZPE">
<scalar units="kcal/mol">-29.5</scalar>
</property>
<property dictRef="me:rotConsts">
<array units="cm-1">0.19971 0.04750 0.04455</array>
</property>
<property dictRef="me:vibFreqs">
<array units="cm-1">70.8637 84.4487 106.9806 173.0253 304.6887 318.7750
428.7644 484.8145 525.1031 603.7048 654.6832 737.5220 787.4276 852.8379 883.6111
953.9823 1004.5478 1053.8957 1064.6069 1128.7833 1198.6969 1253.3244 1278.7220
1334.8805 1410.6218 1452.6993 1489.3660 1497.6437 1505.8495 1566.0557 2722.9610
3067.1244 3104.7070 3126.7407 3152.6138 3153.1238 3175.3520 3191.6923
3263.6723</array>
</property>
<property dictRef="me:frequenciesScaleFactor">
<scalar>1</scalar>
</property>
<property dictRef="me:symmetryNumber">
<scalar>1</scalar>
</property>
<property dictRef="me:MW">
<scalar units="amu">101.04250</scalar>
</property>
<property dictRef="me:spinMultiplicity">

```xml
          <scalar>2</scalar>
        </property>
      </propertyList>
      <me:DOSCMethod name="ClassicalRotors"/>
    </molecule>
    <molecule id="H2O">
      <propertyList>
        <property dictRef="me:ZPE">
          <scalar units="kcal/mol">0.0</scalar>
        </property>
        <property dictRef="me:rotConsts">
          <array units="cm-1">27.82574 14.37796 9.47967</array>
        </property>
        <property dictRef="me:vibFreqs">
          <array units="cm-1">1618.8285 3867.7498 3971.3807</array>
        </property>
        <property dictRef="me:frequenciesScaleFactor">
         <scalar>1.0</scalar>
        </property>
        <property dictRef="me:symmetryNumber">
          <scalar>1</scalar>
        </property>
        <property dictRef="me:MW">
          <scalar units="amu">18.01056</scalar>
        </property>
        <property dictRef="me:spinMultiplicity">
          <scalar>1</scalar>
        </property>
      </propertyList>
      <me:DOSCMethod name="ClassicalRotors"/>
    </molecule>
    <molecule id="N2">
      <propertyList>
        <property dictRef="me:MW">
          <scalar units="amu">28</scalar>
        </property>
        <property dictRef="me:epsilon">
          <scalar>48</scalar>
        </property>
        <property dictRef="me:sigma">
          <scalar>3.9</scalar>
        </property>
      </propertyList>
      <me:DOSCMethod name="ClassicalRotors"/>
    </molecule>
  </moleculeList>
```

```
<reactionList>
   <reaction id="R0">
      <reactant>
         <molecule ref="OH" me:type="deficientReactant" />
      </reactant>
      <reactant>
         <molecule ref="321MBT" me:type="excessReactant" />
      </reactant>
      <product>
         <molecule ref="RC3" me:type="modelled" />
      </product>
      <me:excessReactantConc>1.00E11</me:excessReactantConc>
      <me:MCRCMethod name="MesmerILT"/>
      <me:preExponential units="cm3molecule-1s-1">1.00E-11</me:preExponential>
      <me:activationEnergy units="kcal/mol">0.01</me:activationEnergy>
      <me:TInfinity>298.0</me:TInfinity>
      <me:nInfinity>0.1</me:nInfinity>
   </reaction>
   <reaction id="R1">
      <reactant>
         <molecule ref="RC3" me:type="modelled" />
      </reactant>
      <product>
         <molecule ref="PC6" me:type="modelled" />
      </product>
      <me:MCRCMethod name="SimpleRRKM"/>
      <me:transitionState>
         <molecule ref="TS6" me:type="transitionState" />
      </me:transitionState>
      <me:tunneling name="Eckart"/>
      <me:MCRCMethod name="SimpleRRKM"/>
   </reaction>
   <reaction id="R2">
      <reactant>
         <molecule ref="PC6" me:type="modelled" />
      </reactant>
      <product>
         <molecule ref="P4" me:type="sink" />
      </product>
      <product>
         <molecule ref="H2O" me:type="sink" />
      </product>
      <me:MCRCMethod name="MesmerILT" xsi:type="MesmerILT" >
         <me:preExponential units="cm3molecule-1s-1">1.00E-11</me:preExponential>
         <me:activationEnergy units="kcal/mol" reverse="true">0.01</me:activationEnergy>
   </me:MCRCMethod>
```

```
    </reaction>
</reactionList>
<me:conditions>
    <me:bathGas>N2</me:bathGas>
    <me:PTs>
        <me:PTpair me:units="Torr" me:P="760" me:T="200" me:precision= "qd" />
        <me:PTpair me:units="Torr" me:P="760" me:T="220" me:precision= "qd" />
        <me:PTpair me:units="Torr" me:P="760" me:T="240" me:precision= "qd" />
        <me:PTpair me:units="Torr" me:P="760" me:T="250" me:precision= "qd" />
        <me:PTpair me:units="Torr" me:P="760" me:T="260" me:precision= "qd" />
        <me:PTpair me:units="Torr" me:P="760" me:T="280" me:precision= "qd" />
        <me:PTpair me:units="Torr" me:P="760" me:T="298" me:precision= "qd" />
        <me:PTpair me:units="Torr" me:P="760" me:T="300" me:precision= "qd" />
        <me:PTpair me:units="Torr" me:P="760" me:T="320" me:precision= "qd" />
    </me:PTs>
    <me:InitialPopulation>
        <molecule ref="OH" me:population="1.0"/>
    </me:InitialPopulation>
</me:conditions>
<me:modelParameters>
    <me:grainSize units="cm-1">20</me:grainSize>
    <me:energyAboveTheTopHill>25</me:energyAboveTheTopHill>
</me:modelParameters>
<me:control>
    <me:eigenvalues>1</me:eigenvalues>
    <me:calcMethod>simpleCalc</me:calcMethod>
    <me:testMicroRates/>
    <me:testRateConstants/>
    <me:printGrainDOS/>
    <me:printGrainkfE/>
    <me:testDOS/>
    <me:printSpeciesProfile/>
    <me:printGrainedSpeciesProfile/>
</me:control>
</me:mesmer>
```


Mesmer script for TS7


```
<?xml version="1.0" encoding="utf-8" ?>
<?xml-stylesheet type='text/xsl' href='../../mesmer2.xsl' media='other'?>
<?xml-stylesheet type='text/xsl' href='../../mesmer1.xsl' media='screen'?>
<me:mesmer xmlns="http://www.xml-cml.org/schema"
xmlns:me="http://www.chem.leeds.ac.uk/mesmer"
xmlns:xsi="http://www.w3.org/2001/XMLSchema-instance" xmlns:cml="http://www.xml-
cml.org/schema">
<me:title>321MBT_OH_TS7</me:title>
```

<moleculeList>
<molecule id="321MBT">
<propertyList>
<property dictRef="me:ZPE">
<scalar units="kcal/mol">0.0</scalar>
</property>
<property dictRef="me:rotConsts">
<array units="cm-1">0.19625 0.04349 0.04035</array>
</property>
<property dictRef="me:vibFreqs">
<array units="cm-1">40.2728 115.9719 137.1856 199.4724 238.1814 316.9590
347.5624 402.8403 474.4488 519.2721 704.7871 770.9793 839.5900 887.5999 966.7843
1007.2573 1014.6785 1046.3552 1106.3000 1137.0336 1180.0570 1262.0525 1270.0480
1375.0776 1414.1651 1421.7355 1473.8347 1477.9148 1492.9447 1495.8890 1504.7109
1764.7289 2730.2078 3047.3869 3054.1175 3097.3687 3097.8866 3100.6151 3147.0004
3151.3191 3164.8649 3173.6496</array>
</property>
<property dictRef="me:frequenciesScaleFactor">
<scalar>1.0</scalar>
</property>
<property dictRef="me:symmetryNumber">
<scalar>1</scalar>
</property>
<property dictRef="me:MW">
<scalar units="amu">102.05032</scalar>
</property>
<property dictRef="me:spinMultiplicity">
<scalar>1</scalar>
</property>
</propertyList>
<me:DOSCMethod name="ClassicalRotors"/>
</molecule>
<molecule id="OH">
<propertyList>
<property dictRef="me:ZPE">
<scalar units="kcal/mol">0.0</scalar>
</property>
<property dictRef="me:rotConsts">
<array units="cm-1">18.83025</array>
</property>
<property dictRef="me:vibFreqs">
<array units="cm-1">3768.5403</array>
</property>
<property dictRef="me:frequenciesScaleFactor">
<scalar>1.0</scalar>
</property>

<property dictRef="me:symmetryNumber">
<scalar>1</scalar>
</property>
<property dictRef="me:MW">
<scalar units="amu">17.00274</scalar>
</property>
<property dictRef="me:spinMultiplicity">
<scalar>2</scalar>
</property>
</propertyList>
<me:DOSCMethod name="ClassicalRotors"/>
</molecule>
<molecule id="RC3">
<propertyList>
<property dictRef="me:ZPE">
<scalar units="kcal/mol">-4.2</scalar>
</property>
<property dictRef="me:rotConsts">
<array units="cm-1">0.09554 0.04198 0.03662</array>
</property>
<property dictRef="me:vibFreqs">
<array units="cm-1">35.7787 70.3183 124.2449 134.5829 149.0950 166.9956
212.3151 246.2130 313.5306 352.5367 398.8715 441.4619 471.5616 497.1640 542.3588
677.0556 761.2820 829.7183 883.5424 969.8058 993.5754 1007.5748 1048.6448 1105.4685
1135.4445 1189.7518 1261.3261 1280.1405 1370.7399 1416.1810 1417.3408 1472.0102
1475.0698 1490.8198 1494.6929 1503.2930 1741.5790 2717.6292 3052.9388 3056.2290
3106.6369 3107.3552 3112.5705 3148.3617 3154.0865 3156.7024 3174.6065
3694.8463</array>
</property>
<property dictRef="me:frequenciesScaleFactor">
<scalar>1.0</scalar>
</property>
<property dictRef="me:symmetryNumber">
<scalar>1</scalar>
</property>
<property dictRef="me:MW">
<scalar units="amu">119.05306</scalar>
</property>
<property dictRef="me:spinMultiplicity">
<scalar>2</scalar>
</property>
<property dictRef="me:epsilon">
<scalar>306.5</scalar>
</property>
<property dictRef="me:sigma">
<scalar>4.42</scalar>

</property>
</propertyList>
<me:DOSCMethod name="ClassicalRotors"/>
<me:energyTransferModel xsi:type="me:ExponentialDown">
<me:deltaEDown units="cm-1">200</me:deltaEDown>
</me:energyTransferModel>
</molecule>
<molecule id="TS7">
<propertyList>
<property dictRef="me:ZPE">
<scalar units="kcal/mol">0.3</scalar>
</property>
<property dictRef="me:rotConsts">
<array units="cm-1">0.09072 0.03277 0.02879</array>
</property>
<property dictRef="me:vibFreqs">
<array units="cm-1">16.0204 48.4106 52.9496 88.5500 128.5910 153.4464 175.4564 281.7447 321.4985 369.4382 402.9132 466.6106 526.1255 699.4136 735.1559 776.7005 838.4552 873.3316 942.9405 981.2934 1008.0600 1048.1974 1068.8525 1136.4200 1182.5012 1257.8003 1268.6326 1344.8670 1369.7109 1384.8211 1423.5340 1466.2438 1480.8049 1488.3898 1506.4530 1600.5106 1768.0477 2722.5350 3056.2131 3085.0381 3104.5783 3107.4167 3152.4447 3162.7849 3165.6032 3173.4238 3773.5649</array>
</property>
<property dictRef="me:frequenciesScaleFactor">
<scalar>1.0</scalar>
</property>
<property dictRef="me:symmetryNumber">
<scalar>1</scalar>
</property>
<property dictRef="me:spinMultiplicity">
<scalar>2</scalar>
</property>
<property dictRef="me:imFreqs">
<array units="cm-1">816.9770</array>
</property>
</propertyList>
<me:DOSCMethod name="ClassicalRotors"/>
</molecule>
<molecule id="PC7">
<propertyList>
<property dictRef="me:ZPE">
<scalar units="kcal/mol">-33.3</scalar>
</property>
<property dictRef="me:rotConsts">
<array units="cm-1">0.07804 0.04345 0.03443</array>
</property>

<property dictRef="me:vibFreqs">
<array units="cm-1">55.8438 71.9342 97.6493 118.5241 120.5299 152.0781 196.2032 249.2345 285.5973 302.6743 331.5793 387.7059 436.5461 497.3363 522.3877 613.0238 679.1093 778.3144 789.0915 846.2190 871.6731 963.5673 1006.2403 1040.9771 1064.4964 1128.7595 1211.6587 1245.3430 1279.1541 1390.5166 1421.9198 1433.7368 1487.8844 1492.2097 1502.5765 1539.6187 1618.7390 2694.4757 3064.6384 3099.2954 3124.7513 3152.8415 3156.1097 3163.3306 3185.0587 3260.1016 3805.8521 3935.9889</array>
</property>
<property dictRef="me:frequenciesScaleFactor">
<scalar>1.0</scalar>
</property>
<property dictRef="me:symmetryNumber">
<scalar>1</scalar>
</property>
<property dictRef="me:MW">
<scalar units="amu">119.05306</scalar>
</property>
<property dictRef="me:spinMultiplicity">
<scalar>2</scalar>
</property>
<property dictRef="me:epsilon">
<scalar>306.5</scalar>
</property>
<property dictRef="me:sigma">
<scalar>4.42</scalar>
</property>
</propertyList>
<me:DOSCMethod name="ClassicalRotors"/>
<me:energyTransferModel xsi:type="me:ExponentialDown">
<me:deltaEDown units="cm-1">200</me:deltaEDown>
</me:energyTransferModel>
</molecule>
<molecule id="P4">
<propertyList>
<property dictRef="me:ZPE">
<scalar units="kcal/mol">-29.5</scalar>
</property>
<property dictRef="me:rotConsts">
<array units="cm-1">0.19971 0.04750 0.04455</array>
</property>
<property dictRef="me:vibFreqs">
<array units="cm-1">70.8637 84.4487 106.9806 173.0253 304.6887 318.7750 428.7644 484.8145 525.1031 603.7048 654.6832 737.5220 787.4276 852.8379 883.6111 953.9823 1004.5478 1053.8957 1064.6069 1128.7833 1198.6969 1253.3244 1278.7220 1334.8805 1410.6218 1452.6993 1489.3660 1497.6437 1505.8495 1566.0557 2722.9610

3067.1244 3104.7070 3126.7407 3152.6138 3153.1238 3175.3520 3191.6923
3263.6723</array>
</property>
<property dictRef="me:frequenciesScaleFactor">
<scalar>1</scalar>
</property>
<property dictRef="me:symmetryNumber">
<scalar>1</scalar>
</property>
<property dictRef="me:MW">
<scalar units="amu">101.04250</scalar>
</property>
<property dictRef="me:spinMultiplicity">
<scalar>2</scalar>
</property>
</propertyList>
<me:DOSCMethod name="ClassicalRotors"/>
</molecule>
<molecule id="H2O">
<propertyList>
<property dictRef="me:ZPE">
<scalar units="kcal/mol">0.0</scalar>
</property>
<property dictRef="me:rotConsts">
<array units="cm-1">27.82574 14.37796 9.47967</array>
</property>
<property dictRef="me:vibFreqs">
<array units="cm-1">1618.8285 3867.7498 3971.3807</array>
</property>
<property dictRef="me:frequenciesScaleFactor">
<scalar>1.0</scalar>
</property>
<property dictRef="me:symmetryNumber">
<scalar>1</scalar>
</property>
<property dictRef="me:MW">
<scalar units="amu">18.01056</scalar>
</property>
<property dictRef="me:spinMultiplicity">
<scalar>1</scalar>
</property>
</propertyList>
<me:DOSCMethod name="ClassicalRotors"/>
</molecule>
<molecule id="N2">
<propertyList>

```
      <property dictRef="me:MW">
        <scalar units="amu">28</scalar>
      </property>
      <property dictRef="me:epsilon">
        <scalar>48</scalar>
      </property>
      <property dictRef="me:sigma">
        <scalar>3.9</scalar>
      </property>
    </propertyList>
    <me:DOSCMethod name="ClassicalRotors"/>
  </molecule>
</moleculeList>
<reactionList>
  <reaction id="R0">
    <reactant>
      <molecule ref="OH" me:type="deficientReactant" />
    </reactant>
    <reactant>
      <molecule ref="321MBT" me:type="excessReactant" />
    </reactant>
    <product>
      <molecule ref="RC3" me:type="modelled" />
    </product>
    <me:excessReactantConc>1.00E11</me:excessReactantConc>
    <me:MCRCMethod name="MesmerILT"/>
    <me:preExponential units="cm3molecule-1s-1">1.00E-11</me:preExponential>
    <me:activationEnergy units="kcal/mol">0.01</me:activationEnergy>
    <me:TInfinity>298.0</me:TInfinity>
    <me:nInfinity>0.1</me:nInfinity>
  </reaction>
  <reaction id="R1">
    <reactant>
      <molecule ref="RC3" me:type="modelled" />
    </reactant>
    <product>
      <molecule ref="PC7" me:type="modelled" />
    </product>
    <me:MCRCMethod name="SimpleRRKM"/>
    <me:transitionState>
      <molecule ref="TS7" me:type="transitionState" />
    </me:transitionState>
    <me:tunneling name="Eckart"/>
    <me:MCRCMethod name="SimpleRRKM"/>
  </reaction>
  <reaction id="R2">
```

```xml
      <reactant>
        <molecule ref="PC7" me:type="modelled" />
      </reactant>
      <product>
        <molecule ref="P5" me:type="sink" />
      </product>
      <product>
        <molecule ref="H2O" me:type="sink" />
      </product>
      <me:MCRCMethod name="MesmerILT" xsi:type="MesmerILT" >
        <me:preExponential units="cm3molecule-1s-1">1.00E-11</me:preExponential>
        <me:activationEnergy units="kcal/mol" reverse="true">0.01</me:activationEnergy>
    </me:MCRCMethod>
    </reaction>
  </reactionList>
  <me:conditions>
    <me:bathGas>N2</me:bathGas>
    <me:PTs>
      <me:PTpair me:units="Torr" me:P="760" me:T="200" me:precision= "qd" />
      <me:PTpair me:units="Torr" me:P="760" me:T="220" me:precision= "qd" />
      <me:PTpair me:units="Torr" me:P="760" me:T="240" me:precision= "qd" />
      <me:PTpair me:units="Torr" me:P="760" me:T="250" me:precision= "qd" />
      <me:PTpair me:units="Torr" me:P="760" me:T="260" me:precision= "qd" />
      <me:PTpair me:units="Torr" me:P="760" me:T="280" me:precision= "qd" />
      <me:PTpair me:units="Torr" me:P="760" me:T="298" me:precision= "qd" />
      <me:PTpair me:units="Torr" me:P="760" me:T="300" me:precision= "qd" />
      <me:PTpair me:units="Torr" me:P="760" me:T="320" me:precision= "qd" />
    </me:PTs>
    <me:InitialPopulation>
      <molecule ref="OH" me:population="1.0"/>
    </me:InitialPopulation>
  </me:conditions>
  <me:modelParameters>
    <me:grainSize units="cm-1">20</me:grainSize>
    <me:energyAboveTheTopHill>25</me:energyAboveTheTopHill>
  </me:modelParameters>
  <me:control>
    <me:eigenvalues>1</me:eigenvalues>
    <me:calcMethod>simpleCalc</me:calcMethod>
    <me:testMicroRates/>
    <me:testRateConstants/>
    <me:printGrainDOS/>
    <me:printGrainkfE/>
    <me:testDOS/>
    <me:printSpeciesProfile/>
    <me:printGrainedSpeciesProfile/>
```

</me:control>
</me:mesmer>

Mesmer script for TS8

<?xml version="1.0" encoding="utf-8" ?>
<?xml-stylesheet type='text/xsl' href='../../mesmer2.xsl' media='other'?>
<?xml-stylesheet type='text/xsl' href='../../mesmer1.xsl' media='screen'?>
<me:mesmer xmlns="http://www.xml-cml.org/schema"
xmlns:me="http://www.chem.leeds.ac.uk/mesmer"
xmlns:xsi="http://www.w3.org/2001/XMLSchema-instance" xmlns:cml="http://www.xml-cml.org/schema">
<me:title>321MBT_OH_TS8</me:title>
<moleculeList>
<molecule id="321MBT">
<propertyList>
<property dictRef="me:ZPE">
<scalar units="kcal/mol">0.0</scalar>
</property>
<property dictRef="me:rotConsts">
<array units="cm-1">0.19625 0.04349 0.04035</array>
</property>
<property dictRef="me:vibFreqs">
<array units="cm-1">40.2728 115.9719 137.1856 199.4724 238.1814 316.9590 347.5624 402.8403 474.4488 519.2721 704.7871 770.9793 839.5900 887.5999 966.7843 1007.2573 1014.6785 1046.3552 1106.3000 1137.0336 1180.0570 1262.0525 1270.0480 1375.0776 1414.1651 1421.7355 1473.8347 1477.9148 1492.9447 1495.8890 1504.7109 1764.7289 2730.2078 3047.3869 3054.1175 3097.3687 3097.8866 3100.6151 3147.0004 3151.3191 3164.8649 3173.6496</array>
</property>
<property dictRef="me:frequenciesScaleFactor">
<scalar>1.0</scalar>
</property>
<property dictRef="me:symmetryNumber">
<scalar>1</scalar>
</property>
<property dictRef="me:MW">
<scalar units="amu">102.05032</scalar>
</property>
<property dictRef="me:spinMultiplicity">
<scalar>1</scalar>
</property>
</propertyList>
<me:DOSCMethod name="ClassicalRotors"/>
</molecule>

<molecule id="OH">
<propertyList>
<property dictRef="me:ZPE">
<scalar units="kcal/mol">0.0</scalar>
</property>
<property dictRef="me:rotConsts">
<array units="cm-1">18.83025</array>
</property>
<property dictRef="me:vibFreqs">
<array units="cm-1">3768.5403</array>
</property>
<property dictRef="me:frequenciesScaleFactor">
<scalar>1.0</scalar>
</property>
<property dictRef="me:symmetryNumber">
<scalar>1</scalar>
</property>
<property dictRef="me:MW">
<scalar units="amu">17.00274</scalar>
</property>
<property dictRef="me:spinMultiplicity">
<scalar>2</scalar>
</property>
</propertyList>
<me:DOSCMethod name="ClassicalRotors"/>
</molecule>
<molecule id="RC3">
<propertyList>
<property dictRef="me:ZPE">
<scalar units="kcal/mol">-4.2</scalar>
</property>
<property dictRef="me:rotConsts">
<array units="cm-1">0.09554 0.04198 0.03662</array>
</property>
<property dictRef="me:vibFreqs">
<array units="cm-1">35.7787 70.3183 124.2449 134.5829 149.0950 166.9956
212.3151 246.2130 313.5306 352.5367 398.8715 441.4619 471.5616 497.1640 542.3588
677.0556 761.2820 829.7183 883.5424 969.8058 993.5754 1007.5748 1048.6448 1105.4685
1135.4445 1189.7518 1261.3261 1280.1405 1370.7399 1416.1810 1417.3408 1472.0102
1475.0698 1490.8198 1494.6929 1503.2930 1741.5790 2717.6292 3052.9388 3056.2290
3106.6369 3107.3552 3112.5705 3148.3617 3154.0865 3156.7024 3174.6065
3694.8463</array>
</property>
<property dictRef="me:frequenciesScaleFactor">
<scalar>1.0</scalar>
</property>

<property dictRef="me:symmetryNumber">
<scalar>1</scalar>
</property>
<property dictRef="me:MW">
<scalar units="amu">119.05306</scalar>
</property>
<property dictRef="me:spinMultiplicity">
<scalar>2</scalar>
</property>
<property dictRef="me:epsilon">
<scalar>306.5</scalar>
</property>
<property dictRef="me:sigma">
<scalar>4.42</scalar>
</property>
</propertyList>
<me:DOSCMethod name="ClassicalRotors"/>
<me:energyTransferModel xsi:type="me:ExponentialDown">
<me:deltaEDown units="cm-1">200</me:deltaEDown>
</me:energyTransferModel>
</molecule>
<molecule id="TS8">
<propertyList>
<property dictRef="me:ZPE">
<scalar units="kcal/mol">0.7</scalar>
</property>
<property dictRef="me:rotConsts">
<array units="cm-1">0.15399 0.02466 0.02419</array>
</property>
<property dictRef="me:vibFreqs">
<array units="cm-1">30.6757 41.6766 73.4407 107.8500 145.1592 164.0789
207.2901 292.2301 315.1860 354.6026 410.6572 472.0270 515.7597 707.0783 723.1794
779.1989 842.3616 876.5669 940.9674 989.4162 1010.9132 1047.0812 1070.4799 1137.9524
1181.9020 1259.3732 1267.9818 1344.6340 1375.1342 1393.1437 1423.7420 1462.7199
1480.7076 1486.7340 1502.7156 1576.9646 1763.4371 2728.3617 3057.5925 3083.4232
3098.2185 3106.9773 3153.9214 3163.7114 3167.7461 3176.1543 3779.7212</array>
</property>
<property dictRef="me:frequenciesScaleFactor">
<scalar>1.0</scalar>
</property>
<property dictRef="me:symmetryNumber">
<scalar>1</scalar>
</property>
<property dictRef="me:spinMultiplicity">
<scalar>2</scalar>
</property>

<property dictRef="me:imFreqs">
<array units="cm-1">784.9387</array>
</property>
</propertyList>
<me:DOSCMethod name="ClassicalRotors"/>
</molecule>
<molecule id="PC8">
<propertyList>
<property dictRef="me:ZPE">
<scalar units="kcal/mol">-32.5</scalar>
</property>
<property dictRef="me:rotConsts">
<array units="cm-1">0.07363 0.03875 0.03066</array>
</property>
<property dictRef="me:vibFreqs">
<array units="cm-1">41.5274 56.2853 79.6581 90.3410 122.3193 149.3645 174.8784 184.2657 197.0733 290.4644 303.1590 331.4885 435.7107 494.6252 522.4119 593.0760 671.0966 764.5478 782.9395 843.2608 860.1001 955.7228 1001.6914 1026.2241 1064.4523 1127.4246 1211.5839 1244.5059 1272.1483 1389.2253 1421.2783 1434.2002 1486.2066 1493.1029 1506.1397 1536.0069 1607.8255 2720.8493 3062.1756 3105.0893 3121.0503 3152.6976 3163.9878 3166.7584 3185.6494 3263.6129 3826.1344 3944.4559</array>
</property>
<property dictRef="me:frequenciesScaleFactor">
<scalar>1.0</scalar>
</property>
<property dictRef="me:symmetryNumber">
<scalar>1</scalar>
</property>
<property dictRef="me:MW">
<scalar units="amu">119.05306</scalar>
</property>
<property dictRef="me:spinMultiplicity">
<scalar>2</scalar>
</property>
<property dictRef="me:epsilon">
<scalar>306.5</scalar>
</property>
<property dictRef="me:sigma">
<scalar>4.42</scalar>
</property>
</propertyList>
<me:DOSCMethod name="ClassicalRotors"/>
<me:energyTransferModel xsi:type="me:ExponentialDown">
<me:deltaEDown units="cm-1">200</me:deltaEDown>
</me:energyTransferModel>
</molecule>

<molecule id="P4">
<propertyList>
<property dictRef="me:ZPE">
<scalar units="kcal/mol">-29.4</scalar>
</property>
<property dictRef="me:rotConsts">
<array units="cm-1">0.19971 0.04750 0.04455</array>
</property>
<property dictRef="me:vibFreqs">
<array units="cm-1">70.8637 84.4487 106.9806 173.0253 304.6887 318.7750
428.7644 484.8145 525.1031 603.7048 654.6832 737.5220 787.4276 852.8379 883.6111
953.9823 1004.5478 1053.8957 1064.6069 1128.7833 1198.6969 1253.3244 1278.7220
1334.8805 1410.6218 1452.6993 1489.3660 1497.6437 1505.8495 1566.0557 2722.9610
3067.1244 3104.7070 3126.7407 3152.6138 3153.1238 3175.3520 3191.6923
3263.6723</array>
</property>
<property dictRef="me:frequenciesScaleFactor">
<scalar>1</scalar>
</property>
<property dictRef="me:symmetryNumber">
<scalar>1</scalar>
</property>
<property dictRef="me:MW">
<scalar units="amu">101.04250</scalar>
</property>
<property dictRef="me:spinMultiplicity">
<scalar>2</scalar>
</property>
</propertyList>
<me:DOSCMethod name="ClassicalRotors"/>
</molecule>
<molecule id="H2O">
<propertyList>
<property dictRef="me:ZPE">
<scalar units="kcal/mol">0.0</scalar>
</property>
<property dictRef="me:rotConsts">
<array units="cm-1">27.82574 14.37796 9.47967</array>
</property>
<property dictRef="me:vibFreqs">
<array units="cm-1">1618.8285 3867.7498 3971.3807</array>
</property>
<property dictRef="me:frequenciesScaleFactor">
<scalar>1.0</scalar>
</property>
<property dictRef="me:symmetryNumber">

```xml
        <scalar>1</scalar>
      </property>
      <property dictRef="me:MW">
        <scalar units="amu">18.01056</scalar>
      </property>
      <property dictRef="me:spinMultiplicity">
        <scalar>1</scalar>
      </property>
    </propertyList>
    <me:DOSCMethod name="ClassicalRotors"/>
  </molecule>
  <molecule id="N2">
    <propertyList>
      <property dictRef="me:MW">
        <scalar units="amu">28</scalar>
      </property>
      <property dictRef="me:epsilon">
        <scalar>48</scalar>
      </property>
      <property dictRef="me:sigma">
        <scalar>3.9</scalar>
      </property>
    </propertyList>
    <me:DOSCMethod name="ClassicalRotors"/>
  </molecule>
</moleculeList>
<reactionList>
  <reaction id="R0">
    <reactant>
      <molecule ref="OH" me:type="deficientReactant" />
    </reactant>
    <reactant>
      <molecule ref="321MBT" me:type="excessReactant" />
    </reactant>
    <product>
      <molecule ref="RC3" me:type="modelled" />
    </product>
    <me:excessReactantConc>1.00E11</me:excessReactantConc>
    <me:MCRCMethod name="MesmerILT"/>
    <me:preExponential units="cm3molecule-1s-1">1.00E-11</me:preExponential>
    <me:activationEnergy units="kcal/mol">0.01</me:activationEnergy>
    <me:TInfinity>298.0</me:TInfinity>
    <me:nInfinity>0.1</me:nInfinity>
  </reaction>
  <reaction id="R1">
    <reactant>
```

```
        <molecule ref="RC3" me:type="modelled" />
      </reactant>
      <product>
        <molecule ref="PC8" me:type="modelled" />
      </product>
      <me:MCRCMethod name="SimpleRRKM"/>
      <me:transitionState>
        <molecule ref="TS8" me:type="transitionState" />
      </me:transitionState>
      <me:tunneling name="Eckart"/>
      <me:MCRCMethod name="SimpleRRKM"/>
    </reaction>
    <reaction id="R2">
      <reactant>
        <molecule ref="PC8" me:type="modelled" />
      </reactant>
      <product>
        <molecule ref="P4" me:type="sink" />
      </product>
      <product>
        <molecule ref="H2O" me:type="sink" />
      </product>
      <me:MCRCMethod name="MesmerILT" xsi:type="MesmerILT" >
        <me:preExponential units="cm3molecule-1s-1">1.00E-11</me:preExponential>
        <me:activationEnergy units="kcal/mol" reverse="true">0.01</me:activationEnergy>
    </me:MCRCMethod>
    </reaction>
  </reactionList>
  <me:conditions>
    <me:bathGas>N2</me:bathGas>
    <me:PTs>
      <me:PTpair me:units="Torr" me:P="760" me:T="200" me:precision= "qd" />
      <me:PTpair me:units="Torr" me:P="760" me:T="220" me:precision= "qd" />
      <me:PTpair me:units="Torr" me:P="760" me:T="240" me:precision= "qd" />
      <me:PTpair me:units="Torr" me:P="760" me:T="250" me:precision= "qd" />
      <me:PTpair me:units="Torr" me:P="760" me:T="260" me:precision= "qd" />
      <me:PTpair me:units="Torr" me:P="760" me:T="280" me:precision= "qd" />
      <me:PTpair me:units="Torr" me:P="760" me:T="298" me:precision= "qd" />
      <me:PTpair me:units="Torr" me:P="760" me:T="300" me:precision= "qd" />
      <me:PTpair me:units="Torr" me:P="760" me:T="320" me:precision= "qd" />
    </me:PTs>
    <me:InitialPopulation>
      <molecule ref="OH" me:population="1.0"/>
    </me:InitialPopulation>
  </me:conditions>
  <me:modelParameters>
```

```
    <me:grainSize units="cm-1">20</me:grainSize>
    <me:energyAboveTheTopHill>25</me:energyAboveTheTopHill>
</me:modelParameters>
<me:control>
    <me:eigenvalues>1</me:eigenvalues>
    <me:calcMethod>simpleCalc</me:calcMethod>
    <me:testMicroRates/>
    <me:testRateConstants/>
    <me:printGrainDOS/>
    <me:printGrainkfE/>
    <me:testDOS/>
    <me:printSpeciesProfile/>
    <me:printGrainedSpeciesProfile/>
</me:control>
</me:mesmer>
```


Mesmer script for TS9


```
<?xml version="1.0" encoding="utf-8" ?>
<?xml-stylesheet type='text/xsl' href='../../mesmer2.xsl' media='other'?>
<?xml-stylesheet type='text/xsl' href='../../mesmer1.xsl' media='screen'?>
<me:mesmer xmlns="http://www.xml-cml.org/schema"
xmlns:me="http://www.chem.leeds.ac.uk/mesmer"
xmlns:xsi="http://www.w3.org/2001/XMLSchema-instance" xmlns:cml="http://www.xml-
cml.org/schema">
<me:title>321MBT_OH_TS9</me:title>
<moleculeList>
    <molecule id="321MBT">
        <propertyList>
            <property dictRef="me:ZPE">
                <scalar units="kcal/mol">0.0</scalar>
            </property>
            <property dictRef="me:rotConsts">
                <array units="cm-1">0.19625 0.04349 0.04035</array>
            </property>
            <property dictRef="me:vibFreqs">
                <array units="cm-1">40.2728 115.9719 137.1856 199.4724 238.1814 316.9590
347.5624 402.8403 474.4488 519.2721 704.7871 770.9793 839.5900 887.5999 966.7843
1007.2573 1014.6785 1046.3552 1106.3000 1137.0336 1180.0570 1262.0525 1270.0480
1375.0776 1414.1651 1421.7355 1473.8347 1477.9148 1492.9447 1495.8890 1504.7109
1764.7289 2730.2078 3047.3869 3054.1175 3097.3687 3097.8866 3100.6151 3147.0004
3151.3191 3164.8649 3173.6496</array>
            </property>
            <property dictRef="me:frequenciesScaleFactor">
              <scalar>1.0</scalar>
            </property>
```

<property dictRef="me:symmetryNumber">
<scalar>1</scalar>
</property>
<property dictRef="me:MW">
<scalar units="amu">102.05032</scalar>
</property>
<property dictRef="me:spinMultiplicity">
<scalar>1</scalar>
</property>
</propertyList>
<me:DOSCMethod name="ClassicalRotors"/>
</molecule>
<molecule id="OH">
<propertyList>
<property dictRef="me:ZPE">
<scalar units="kcal/mol">0.0</scalar>
</property>
<property dictRef="me:rotConsts">
<array units="cm-1">18.83025</array>
</property>
<property dictRef="me:vibFreqs">
<array units="cm-1">3768.5403</array>
</property>
<property dictRef="me:frequenciesScaleFactor">
<scalar>1.0</scalar>
</property>
<property dictRef="me:symmetryNumber">
<scalar>1</scalar>
</property>
<property dictRef="me:MW">
<scalar units="amu">17.00274</scalar>
</property>
<property dictRef="me:spinMultiplicity">
<scalar>2</scalar>
</property>
</propertyList>
<me:DOSCMethod name="ClassicalRotors"/>
</molecule>
<molecule id="RC3">
<propertyList>
<property dictRef="me:ZPE">
<scalar units="kcal/mol">-4.2</scalar>
</property>
<property dictRef="me:rotConsts">
<array units="cm-1">0.09554 0.04198 0.03662</array>
</property>

<property dictRef="me:vibFreqs">
<array units="cm-1">35.7787 70.3183 124.2449 134.5829 149.0950 166.9956
212.3151 246.2130 313.5306 352.5367 398.8715 441.4619 471.5616 497.1640 542.3588
677.0556 761.2820 829.7183 883.5424 969.8058 993.5754 1007.5748 1048.6448 1105.4685
1135.4445 1189.7518 1261.3261 1280.1405 1370.7399 1416.1810 1417.3408 1472.0102
1475.0698 1490.8198 1494.6929 1503.2930 1741.5790 2717.6292 3052.9388 3056.2290
3106.6369 3107.3552 3112.5705 3148.3617 3154.0865 3156.7024 3174.6065
3694.8463</array>
</property>
<property dictRef="me:frequenciesScaleFactor">
<scalar>1.0</scalar>
</property>
<property dictRef="me:symmetryNumber">
<scalar>1</scalar>
</property>
<property dictRef="me:MW">
<scalar units="amu">119.05306</scalar>
</property>
<property dictRef="me:spinMultiplicity">
<scalar>2</scalar>
</property>
<property dictRef="me:epsilon">
<scalar>306.5</scalar>
</property>
<property dictRef="me:sigma">
<scalar>4.42</scalar>
</property>
</propertyList>
<me:DOSCMethod name="ClassicalRotors"/>
<me:energyTransferModel xsi:type="me:ExponentialDown">
<me:deltaEDown units="cm-1">200</me:deltaEDown>
</me:energyTransferModel>
</molecule>
<molecule id="TS9">
<propertyList>
<property dictRef="me:ZPE">
<scalar units="kcal/mol">-3.6</scalar>
</property>
<property dictRef="me:rotConsts">
<array units="cm-1">0.09389 0.03807 0.03293</array>
</property>
<property dictRef="me:vibFreqs">
<array units="cm-1">26.2264 47.2168 91.8250 114.8459 129.7682 159.1357
186.7684 257.4149 320.4654 345.2027 400.1665 470.0443 524.5307 607.6665 706.4594
771.2090 838.5007 893.8448 965.7258 1000.7088 1006.0154 1048.3292 1096.6255 1139.7318
1183.8080 1261.9979 1269.6890 1372.8911 1415.1093 1418.7983 1468.8109 1475.5142

1487.5908 1492.8336 1500.7525 1688.5043 2724.3976 3047.1545 3051.1969 3094.4425 3102.9133 3109.2972 3152.5627 3157.1357 3164.3036 3195.9171 3796.9432 </array>
</property>
<property dictRef="me:frequenciesScaleFactor">
<scalar>1.0</scalar>
</property>
<property dictRef="me:symmetryNumber">
<scalar>1</scalar>
</property>
<property dictRef="me:spinMultiplicity">
<scalar>2</scalar>
</property>
<property dictRef="me:imFreqs">
<array units="cm-1">163.3461</array>
</property>
</propertyList>
<me:DOSCMethod name="ClassicalRotors"/>
</molecule>
<molecule id="P5">
<propertyList>
<property dictRef="me:ZPE">
<scalar units="kcal/mol">-28.4</scalar>
</property>
<property dictRef="me:rotConsts">
<array units="cm-1">0.09246 0.04614 0.03902</array>
</property>
<property dictRef="me:vibFreqs">
<array units="cm-1">48.2695 95.0670 110.7283 128.7449 168.5714 224.3486 241.9731 299.7620 348.4495 362.2111 400.6299 490.8469 546.5858 706.2912 783.0783 839.4414 934.2286 964.5744 983.5206 998.8696 1041.8782 1059.4890 1103.4461 1164.5261 1204.4894 1264.9071 1301.1823 1320.8069 1387.4638 1403.5968 1409.9486 1423.9114 1468.3775 1479.3799 1480.2073 1494.6184 1500.5191 2729.9402 2983.2072 3003.6207 3078.5631 3084.4952 3086.4299 3095.4275 3126.1039 3129.9591 3142.9208 3858.7365</array>
</property>
<property dictRef="me:frequenciesScaleFactor">
<scalar>1.0</scalar>
</property>
<property dictRef="me:symmetryNumber">
<scalar>1</scalar>
</property>
<property dictRef="me:MW">
<scalar units="amu">119.05306</scalar>
</property>
<property dictRef="me:spinMultiplicity">
<scalar>2</scalar>

```
          </property>
          <property dictRef="me:epsilon">
            <scalar>306.5</scalar>
          </property>
          <property dictRef="me:sigma">
            <scalar>4.42</scalar>
          </property>
        </propertyList>
        <me:DOSCMethod name="ClassicalRotors"/>
        <me:energyTransferModel xsi:type="me:ExponentialDown">
          <me:deltaEDown units="cm-1">200</me:deltaEDown>
        </me:energyTransferModel>
      </molecule>
      <molecule id="N2">
        <propertyList>
          <property dictRef="me:MW">
            <scalar units="amu">28</scalar>
          </property>
          <property dictRef="me:epsilon">
            <scalar>48</scalar>
          </property>
          <property dictRef="me:sigma">
            <scalar>3.9</scalar>
          </property>
        </propertyList>
        <me:DOSCMethod name="ClassicalRotors"/>
      </molecule>
    </moleculeList>
    <reactionList>
      <reaction id="R0">
        <reactant>
          <molecule ref="OH" me:type="deficientReactant" />
        </reactant>
        <reactant>
          <molecule ref="321MBT" me:type="excessReactant" />
        </reactant>
        <product>
          <molecule ref="RC3" me:type="modelled" />
        </product>
        <me:excessReactantConc>1.00E11</me:excessReactantConc>
        <me:MCRCMethod name="MesmerILT"/>
        <me:preExponential units="cm3molecule-1s-1">1.00E-11</me:preExponential>
        <me:activationEnergy units="kcal/mol">0.01</me:activationEnergy>
        <me:TInfinity>298.0</me:TInfinity>
        <me:nInfinity>0.1</me:nInfinity>
      </reaction>
```

```
    <reaction id="R1">
      <reactant>
        <molecule ref="RC3" me:type="modelled" />
      </reactant>
      <product>
        <molecule ref="P5" me:type="modelled" />
      </product>
      <me:MCRCMethod name="SimpleRRKM"/>
      <me:transitionState>
        <molecule ref="TS9" me:type="transitionState" />
      </me:transitionState>
      <me:tunneling name="Eckart"/>
      <me:MCRCMethod name="SimpleRRKM"/>
    </reaction>
  </reactionList>
  <me:conditions>
    <me:bathGas>N2</me:bathGas>
    <me:PTs>
      <me:PTpair me:units="Torr" me:P="760" me:T="200" me:precision= "qd" />
      <me:PTpair me:units="Torr" me:P="760" me:T="220" me:precision= "qd" />
      <me:PTpair me:units="Torr" me:P="760" me:T="240" me:precision= "qd" />
      <me:PTpair me:units="Torr" me:P="760" me:T="250" me:precision= "qd" />
      <me:PTpair me:units="Torr" me:P="760" me:T="260" me:precision= "qd" />
      <me:PTpair me:units="Torr" me:P="760" me:T="280" me:precision= "qd" />
      <me:PTpair me:units="Torr" me:P="760" me:T="298" me:precision= "qd" />
      <me:PTpair me:units="Torr" me:P="760" me:T="300" me:precision= "qd" />
      <me:PTpair me:units="Torr" me:P="760" me:T="320" me:precision= "qd" />
    </me:PTs>
    <me:InitialPopulation>
      <molecule ref="OH" me:population="1.0"/>
    </me:InitialPopulation>
  </me:conditions>
  <me:modelParameters>
    <me:grainSize units="cm-1">20</me:grainSize>
    <me:energyAboveTheTopHill>25</me:energyAboveTheTopHill>
  </me:modelParameters>
  <me:control>
    <me:eigenvalues>1</me:eigenvalues>
    <me:calcMethod>simpleCalc</me:calcMethod>
    <me:testMicroRates/>
    <me:testRateConstants/>
    <me:printGrainDOS/>
    <me:printGrainkfE/>
    <me:testDOS/>
    <me:printSpeciesProfile/>
    <me:printGrainedSpeciesProfile/>
```

```
</me:control>
</me:mesmer>
```


Mesmer script for TS10


```
<?xml version="1.0" encoding="utf-8" ?>
<?xml-stylesheet type='text/xsl' href='../../mesmer2.xsl' media='other'?>
<?xml-stylesheet type='text/xsl' href='../../mesmer1.xsl' media='screen'?>
<me:mesmer xmlns="http://www.xml-cml.org/schema"
xmlns:me="http://www.chem.leeds.ac.uk/mesmer"
xmlns:xsi="http://www.w3.org/2001/XMLSchema-instance" xmlns:cml="http://www.xml-
cml.org/schema">
<me:title>321MBT_OH_TS10</me:title>
<moleculeList>
  <molecule id="321MBT">
    <propertyList>
      <property dictRef="me:ZPE">
        <scalar units="kcal/mol">0.0</scalar>
      </property>
      <property dictRef="me:rotConsts">
        <array units="cm-1">0.19625 0.04349 0.04035</array>
      </property>
      <property dictRef="me:vibFreqs">
        <array units="cm-1">40.2728 115.9719 137.1856 199.4724 238.1814 316.9590
347.5624 402.8403 474.4488 519.2721 704.7871 770.9793 839.5900 887.5999 966.7843
1007.2573 1014.6785 1046.3552 1106.3000 1137.0336 1180.0570 1262.0525 1270.0480
1375.0776 1414.1651 1421.7355 1473.8347 1477.9148 1492.9447 1495.8890 1504.7109
1764.7289 2730.2078 3047.3869 3054.1175 3097.3687 3097.8866 3100.6151 3147.0004
3151.3191 3164.8649 3173.6496</array>
      </property>
      <property dictRef="me:frequenciesScaleFactor">
       <scalar>1.0</scalar>
      </property>
      <property dictRef="me:symmetryNumber">
        <scalar>1</scalar>
      </property>
      <property dictRef="me:MW">
        <scalar units="amu">102.05032</scalar>
      </property>
      <property dictRef="me:spinMultiplicity">
        <scalar>1</scalar>
      </property>
    </propertyList>
    <me:DOSCMethod name="ClassicalRotors"/>
  </molecule>
```

<molecule id="OH">
<propertyList>
<property dictRef="me:ZPE">
<scalar units="kcal/mol">0.0</scalar>
</property>
<property dictRef="me:rotConsts">
<array units="cm-1">18.83025</array>
</property>
<property dictRef="me:vibFreqs">
<array units="cm-1">3768.5403</array>
</property>
<property dictRef="me:frequenciesScaleFactor">
<scalar>1.0</scalar>
</property>
<property dictRef="me:symmetryNumber">
<scalar>1</scalar>
</property>
<property dictRef="me:MW">
<scalar units="amu">17.00274</scalar>
</property>
<property dictRef="me:spinMultiplicity">
<scalar>2</scalar>
</property>
</propertyList>
<me:DOSCMethod name="ClassicalRotors"/>
</molecule>
<molecule id="RC3">
<propertyList>
<property dictRef="me:ZPE">
<scalar units="kcal/mol">-4.2</scalar>
</property>
<property dictRef="me:rotConsts">
<array units="cm-1">0.09554 0.04198 0.03662</array>
</property>
<property dictRef="me:vibFreqs">
<array units="cm-1">35.7787 70.3183 124.2449 134.5829 149.0950 166.9956
212.3151 246.2130 313.5306 352.5367 398.8715 441.4619 471.5616 497.1640 542.3588
677.0556 761.2820 829.7183 883.5424 969.8058 993.5754 1007.5748 1048.6448 1105.4685
1135.4445 1189.7518 1261.3261 1280.1405 1370.7399 1416.1810 1417.3408 1472.0102
1475.0698 1490.8198 1494.6929 1503.2930 1741.5790 2717.6292 3052.9388 3056.2290
3106.6369 3107.3552 3112.5705 3148.3617 3154.0865 3156.7024 3174.6065
3694.8463</array>
</property>
<property dictRef="me:frequenciesScaleFactor">
<scalar>1.0</scalar>
</property>

<property dictRef="me:symmetryNumber">
<scalar>1</scalar>
</property>
<property dictRef="me:MW">
<scalar units="amu">119.05306</scalar>
</property>
<property dictRef="me:spinMultiplicity">
<scalar>2</scalar>
</property>
<property dictRef="me:epsilon">
<scalar>306.5</scalar>
</property>
<property dictRef="me:sigma">
<scalar>4.42</scalar>
</property>
</propertyList>
<me:DOSCMethod name="ClassicalRotors"/>
<me:energyTransferModel xsi:type="me:ExponentialDown">
<me:deltaEDown units="cm-1">200</me:deltaEDown>
</me:energyTransferModel>
</molecule>
<molecule id="TS10">
<propertyList>
<property dictRef="me:ZPE">
<scalar units="kcal/mol">-4.1</scalar>
</property>
<property dictRef="me:rotConsts">
<array units="cm-1">0.10980 0.03675 0.03400</array>
</property>
<property dictRef="me:vibFreqs">
<array units="cm-1">32.0942 101.0686 118.9414 127.0030 168.5388 212.3980
241.4481 266.0674 322.3978 352.7545 402.1087 420.2565 527.0139 666.1398 694.4273
766.3680 828.3863 875.4150 966.2979 997.2202 1001.8028 1049.4057 1084.3023 1138.8260
1180.6026 1261.7491 1269.5908 1369.6422 1411.2670 1421.4273 1467.7326 1476.7156
1487.8799 1496.2318 1502.7733 1667.1187 2722.8291 3054.1110 3060.1918 3111.1732
3116.5225 3125.7223 3147.5319 3162.9815 3172.3329 3182.4874 3806.3731</array>
</property>
<property dictRef="me:frequenciesScaleFactor">
<scalar>1.0</scalar>
</property>
<property dictRef="me:symmetryNumber">
<scalar>1</scalar>
</property>
<property dictRef="me:spinMultiplicity">
<scalar>2</scalar>
</property>

<property dictRef="me:imFreqs">
<array units="cm-1">154.6911</array>
</property>
</propertyList>
<me:DOSCMethod name="ClassicalRotors"/>
</molecule>
<molecule id="P6">
<propertyList>
<property dictRef="me:ZPE">
<scalar units="kcal/mol">-30.5</scalar>
</property>
<property dictRef="me:rotConsts">
<array units="cm-1">0.13385 0.03675 0.03569</array>
</property>
<property dictRef="me:vibFreqs">
<array units="cm-1">67.9592 100.0784 224.6593 233.9199 252.1854 291.1269
307.4486 338.7248 340.7004 364.2212 407.5621 442.9710 541.5649 562.2948 666.9060
732.7660 821.1153 914.7033 942.6104 978.4418 993.0770 1024.5720 1121.5136 1128.4343
1179.7350 1229.6198 1238.8820 1256.4236 1347.3065 1393.1077 1408.1080 1416.8142
1475.1413 1483.5874 1490.3369 1502.9972 1511.8245 2741.3841 3056.2361 3062.4746
3084.1979 3127.5417 3137.8090 3143.7881 3144.8436 3148.4694 3197.1370
3850.8426</array>
</property>
<property dictRef="me:frequenciesScaleFactor">
<scalar>1.0</scalar>
</property>
<property dictRef="me:symmetryNumber">
<scalar>1</scalar>
</property>
<property dictRef="me:MW">
<scalar units="amu">119.05306</scalar>
</property>
<property dictRef="me:spinMultiplicity">
<scalar>2</scalar>
</property>
<property dictRef="me:epsilon">
<scalar>306.5</scalar>
</property>
<property dictRef="me:sigma">
<scalar>4.42</scalar>
</property>
</propertyList>
<me:DOSCMethod name="ClassicalRotors"/>
<me:energyTransferModel xsi:type="me:ExponentialDown">
<me:deltaEDown units="cm-1">200</me:deltaEDown>
</me:energyTransferModel>

```xml
    </molecule>
    <molecule id="N2">
      <propertyList>
        <property dictRef="me:MW">
          <scalar units="amu">28</scalar>
        </property>
        <property dictRef="me:epsilon">
          <scalar>48</scalar>
        </property>
        <property dictRef="me:sigma">
          <scalar>3.9</scalar>
        </property>
      </propertyList>
      <me:DOSCMethod name="ClassicalRotors"/>
    </molecule>
  </moleculeList>
  <reactionList>
    <reaction id="R0">
      <reactant>
        <molecule ref="OH" me:type="deficientReactant" />
      </reactant>
      <reactant>
        <molecule ref="321MBT" me:type="excessReactant" />
      </reactant>
      <product>
        <molecule ref="RC3" me:type="modelled" />
      </product>
      <me:excessReactantConc>1.00E11</me:excessReactantConc>
      <me:MCRCMethod name="MesmerILT"/>
      <me:preExponential units="cm3molecule-1s-1">1.00E-11</me:preExponential>
      <me:activationEnergy units="kcal/mol">0.01</me:activationEnergy>
      <me:TInfinity>298.0</me:TInfinity>
      <me:nInfinity>0.1</me:nInfinity>
    </reaction>
    <reaction id="R1">
      <reactant>
        <molecule ref="RC3" me:type="modelled" />
      </reactant>
      <product>
        <molecule ref="P6" me:type="modelled" />
      </product>
      <me:MCRCMethod name="SimpleRRKM"/>
      <me:transitionState>
        <molecule ref="TS10" me:type="transitionState" />
      </me:transitionState>
      <me:tunneling name="Eckart"/>
```

```
      <me:MCRCMethod name="SimpleRRKM"/>
    </reaction>
  </reactionList>
  <me:conditions>
    <me:bathGas>N2</me:bathGas>
    <me:PTs>
      <me:PTpair me:units="Torr" me:P="760" me:T="200" me:precision= "qd" />
      <me:PTpair me:units="Torr" me:P="760" me:T="220" me:precision= "qd" />
      <me:PTpair me:units="Torr" me:P="760" me:T="240" me:precision= "qd" />
      <me:PTpair me:units="Torr" me:P="760" me:T="250" me:precision= "qd" />
      <me:PTpair me:units="Torr" me:P="760" me:T="260" me:precision= "qd" />
      <me:PTpair me:units="Torr" me:P="760" me:T="280" me:precision= "qd" />
      <me:PTpair me:units="Torr" me:P="760" me:T="298" me:precision= "qd" />
      <me:PTpair me:units="Torr" me:P="760" me:T="300" me:precision= "qd" />
      <me:PTpair me:units="Torr" me:P="760" me:T="320" me:precision= "qd" />
    </me:PTs>
    <me:InitialPopulation>
      <molecule ref="OH" me:population="1.0"/>
    </me:InitialPopulation>
  </me:conditions>
  <me:modelParameters>
    <me:grainSize units="cm-1">20</me:grainSize>
    <me:energyAboveTheTopHill>25</me:energyAboveTheTopHill>
  </me:modelParameters>
  <me:control>
    <me:eigenvalues>1</me:eigenvalues>
    <me:calcMethod>simpleCalc</me:calcMethod>
    <me:testMicroRates/>
    <me:testRateConstants/>
    <me:printGrainDOS/>
    <me:printGrainkfE/>
    <me:testDOS/>
    <me:printSpeciesProfile/>
    <me:printGrainedSpeciesProfile/>
  </me:control>
</me:mesmer>
```

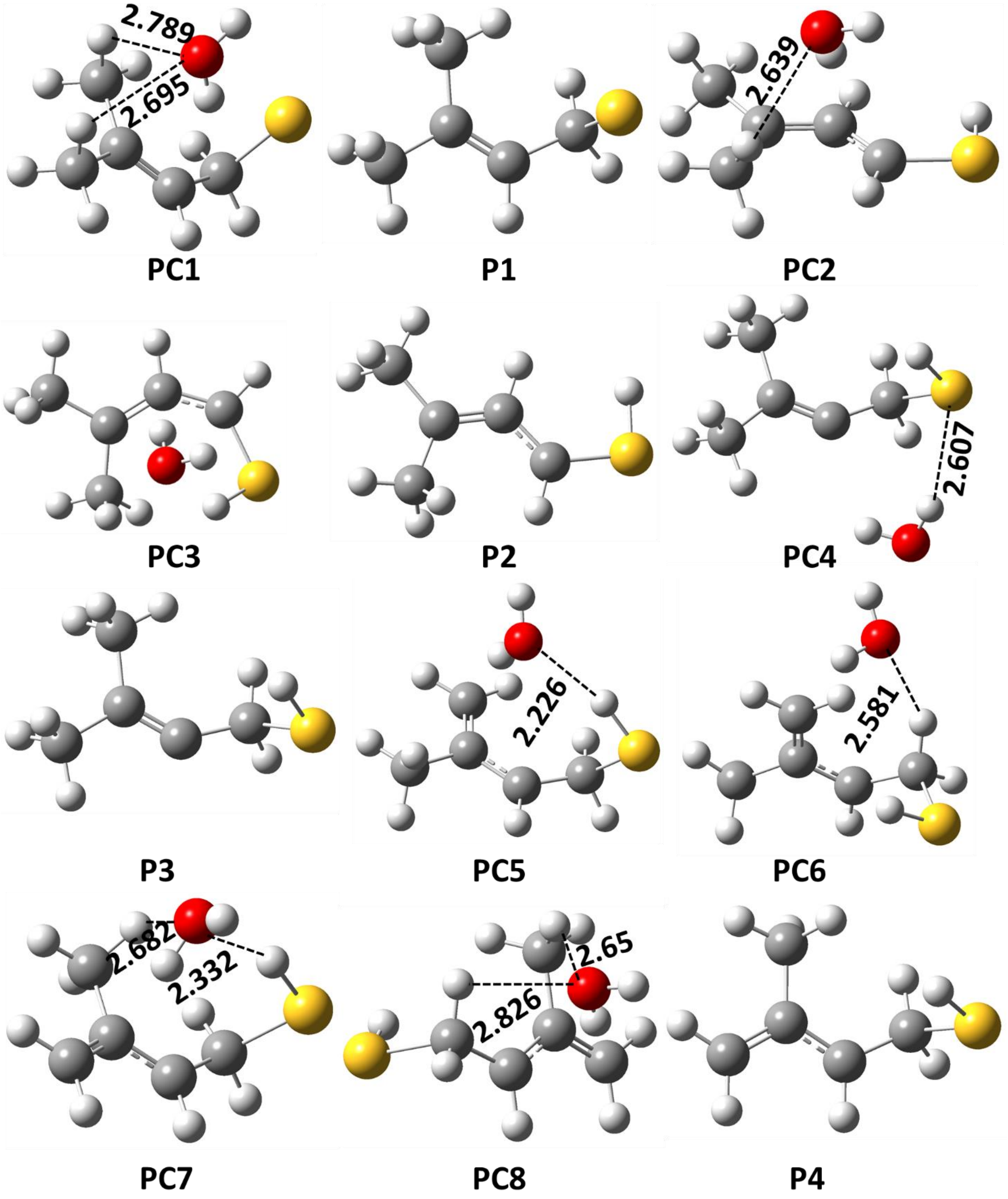

Figure S1. Optimized geometries of post-reactive complexes and products for the H-abstraction and addition paths associated with the MBT + •OH reaction obtained at the M06-2X/aug-cc-pV(T+d)Z level of theory. The black, yellow, red, and white colors denote C, S, O, and H-atoms, respectively.

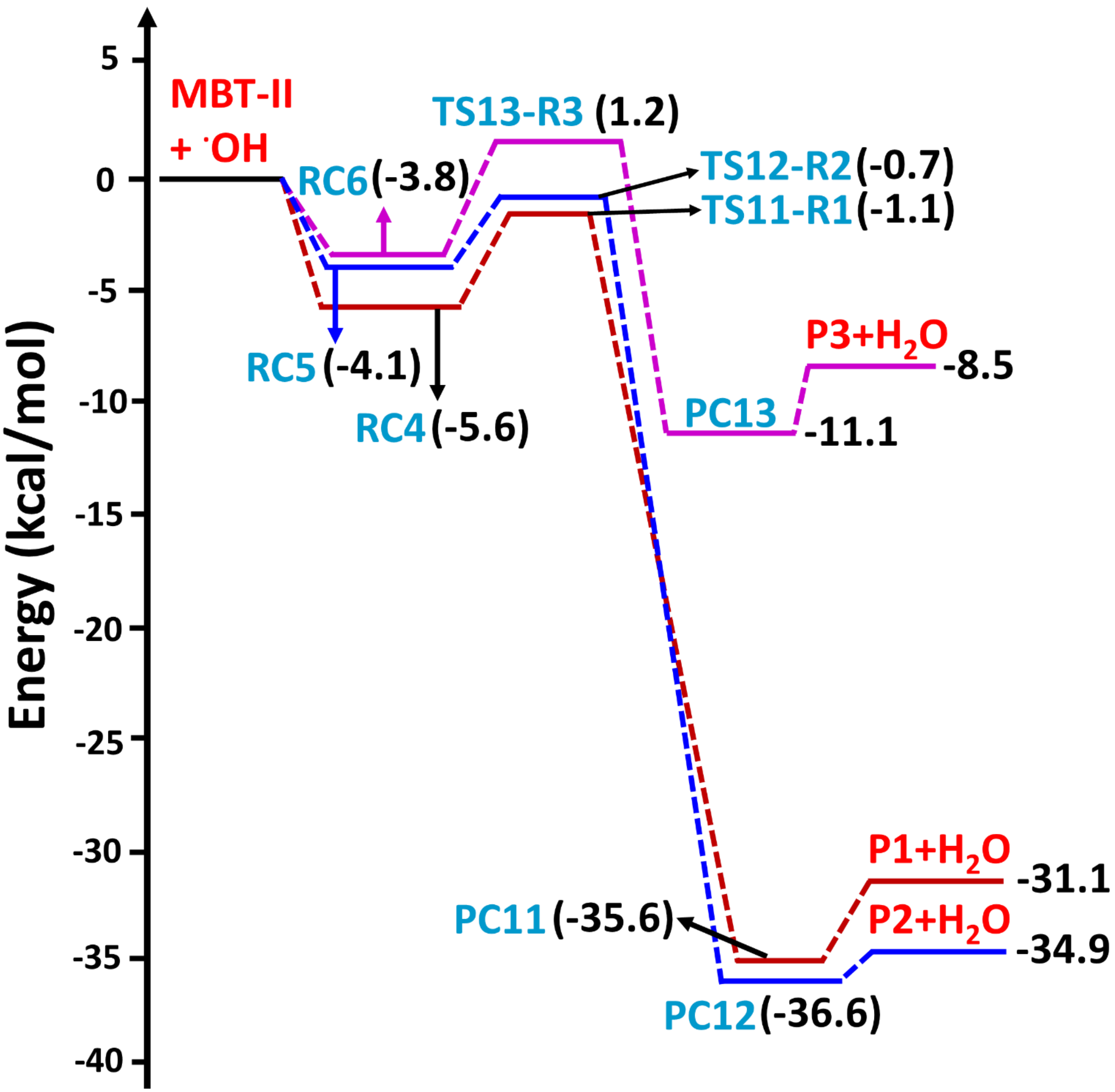

Figure S2. The potential energy surface profiles for H-abstraction from the -SH, $-CH_2$, and -CH sites in the reaction of MBT-II + OH radical computed at the CCSD(T)/aug-cc-pV(T+d)Z//M06-2X/aug-cc-pV(T+d)Z level of theory. The RCs, TSs, PCs, and Ps refer to pre-reactive complexes, transition states, product complexes and products, respectively.

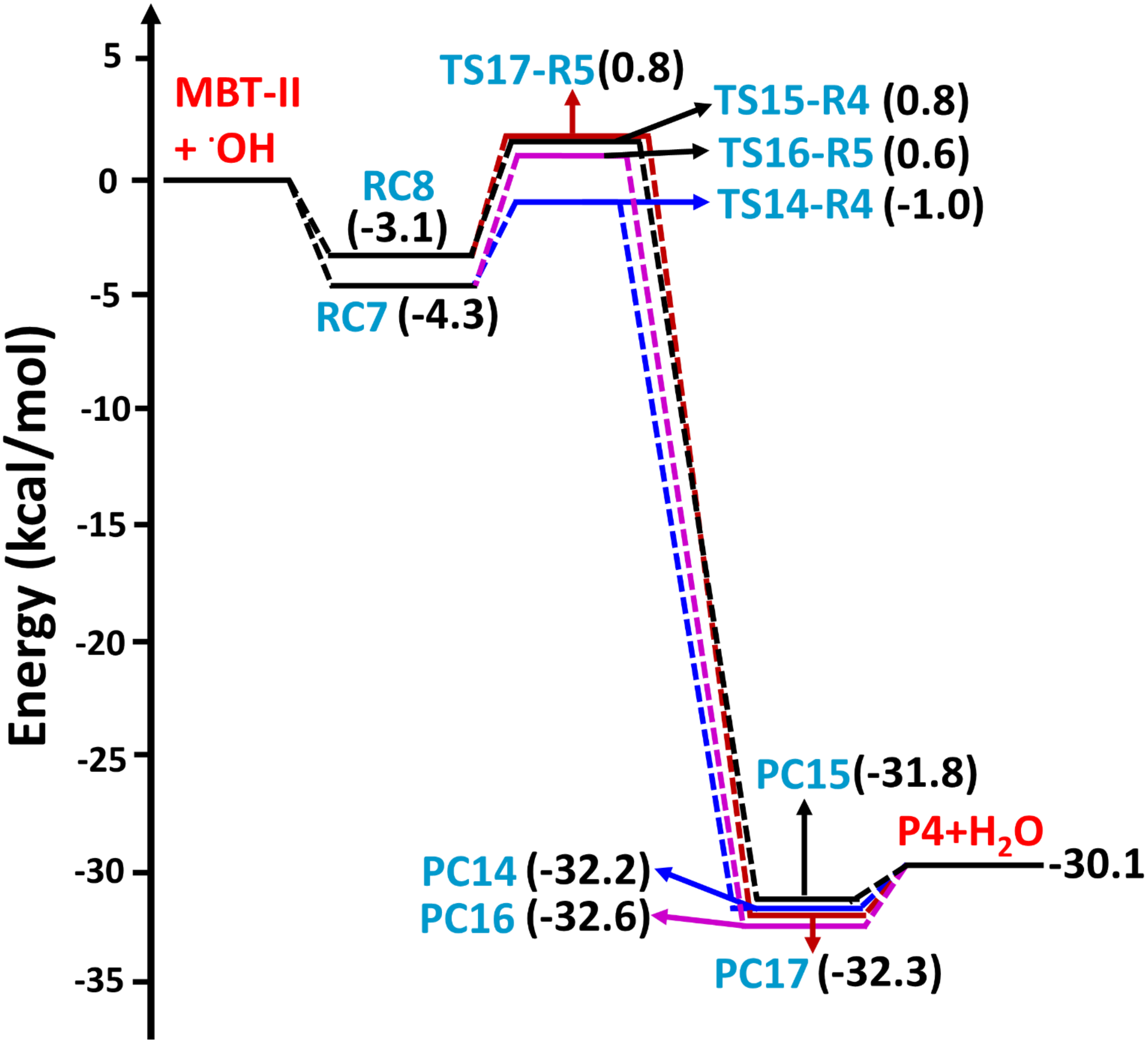

Figure S3. The potential energy surface profiles for H-abstraction from the two methyl groups involved in the reaction of MBT-II + OH radical computed at the CCSD(T)/aug-cc-pV(T+d)Z//M06-2X/aug-cc-pV(T+d)Z level of theory. The RCs, TSs, PCs, and Ps refer to pre-reactive complexes, transition states, product complexes and products, respectively.

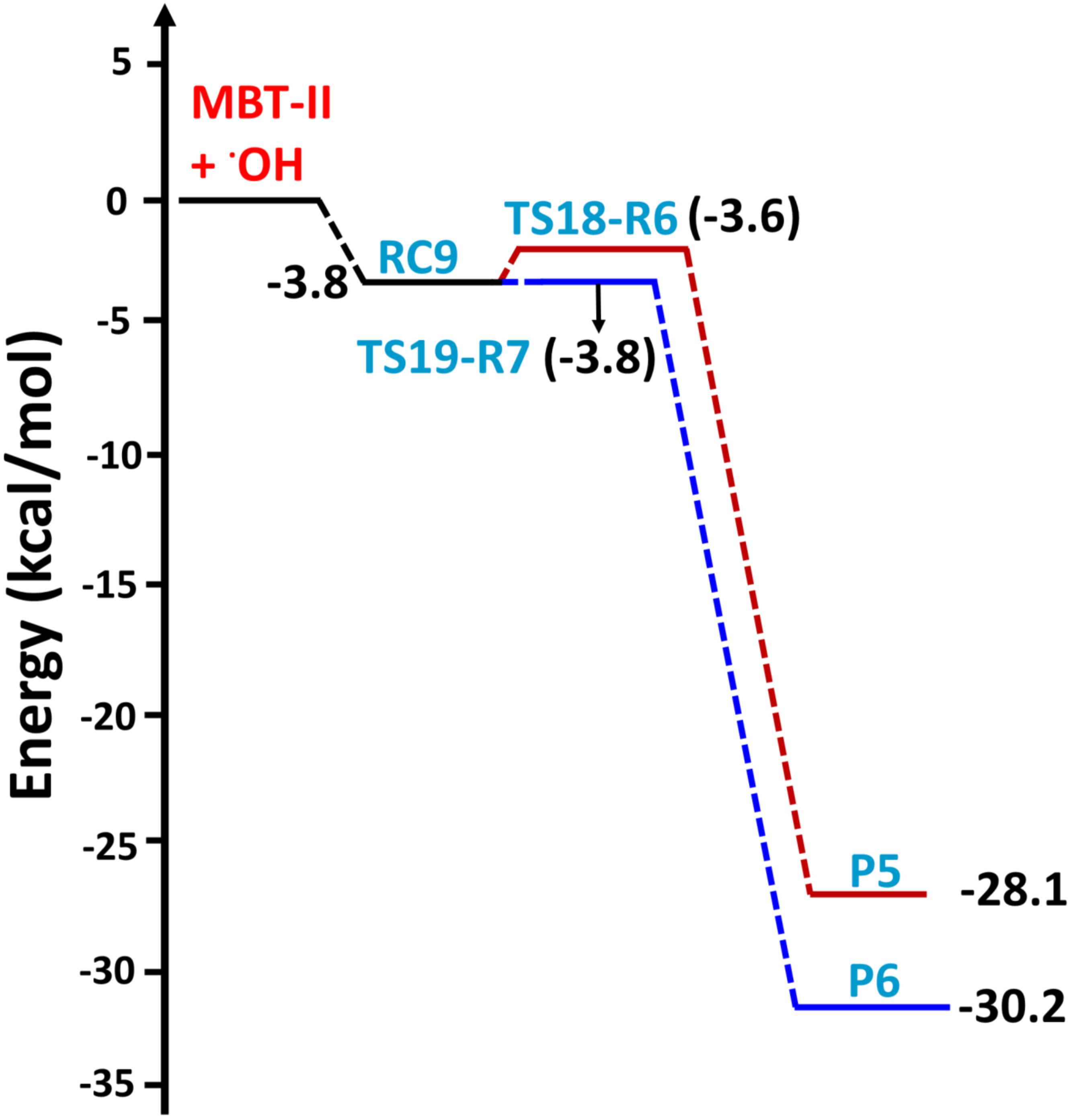

Figure S4. The potential energy surface profiles for the two possible OH addition paths involved in the reaction of MBT-II + OH radical computed at the CCSD(T)/aug-cc-pV(T+d)Z//M06-2X/aug-cc-pV(T+d)Z level of theory. The RCs, TSs and Ps refer to pre-reactive complexes, transition states, and products, respectively.

Master equation simulations:[1,2]

In essence, the energy associated with the intermediate complexes along the potential energy surfaces (PESs) is partitioned into discrete units, which serve as the foundational components for constructing the chemical master equation model.[1,2] The specific formulation of the energy-grained master equation employed in this study is presented in eqn. 1[1,2]

$$\frac{\mathrm{d}}{\mathrm{dt}}\mathbf{p} = \mathbf{Mp} \qquad (1)$$

In eqn. 1, 'p' represents a vector that encompasses the population distributions, while 'M' is a matrix responsible for determining how these energy grains within the populations evolve through collision-induced energy transfer processes. These energy transfer events are characterized using an exponential decay model.[1,2]

We employed a single exponential down model with an average transfer energy of ΔE_d = 200 cm^{-1} to simulate the collision energy transfer processes between the RCs and PCs, and the bath gas (N_2) in the various possible H-abstraction and addition paths involving MBT-I + OH radical and MBT-II + OH radical reactions. This choice of ΔE_d was based on prior studies in the literature that investigated analogous reactions with OH radicals.[2, 3]

In addition to this, Lennard-Jones' (L-J) potentials were essential for computing collision frequencies. Therefore, we adopted the L-J parameters for the RCs and PCs involved in the PES profiles associated with the MBT-I + OH radical and MBT-II + OH radical reactions. The RCs and PC's L-J parameters used were σ = 4.42 Å and ε = 306.5 K. These L-J parameters were derived based on the nearest-sized alkane.[4] For the bath gas, N_2 was used in the calculations, and its L-J parameters were σ = 3.9 Å and ε = 48 K as per Glowacki et al.[5]

The zero-point-corrected energies for all stationary points located on the PESs as shown in Figures 3, 4 and 5 for the MBT-I + OH radical reaction, and Figures S1, S2 and S3 for MBT-II + OH radical reaction respectively, along with the rotational constants and vibrational frequencies, were obtained from the current calculations. These data were utilized as input parameters for the Mesmer rate calculations. The interaction between MBT-I and OH radical, and MBT-II and OH radical respectively, to form the corresponding intermediate complexes is barrierless. Therefore, for these barrierless reactions, we employed the Inverse Laplace Transform (ILT) method.[5] The Arrhenius pre-exponential factor utilized in the ILT approach for the MBT-I + OH radical and MBT-II + OH radical barrierless reactions was 1.0×10^{-11} cm^3 $molecule^{-1}$ s^{-1}, as shown in Figures 3, 4 and 5. We set an activation energy value of 0 kcal mol^{-1} and a modified Arrhenius parameter of 0.1 for all the barrierless reaction pathways used in the rate coefficient calculations. To consider the effects of tunneling corrections on reaction rates, we applied the Eckart tunneling correction method.[6]

References:

1. Arathala, P.; Tangtartharakul, C. B.; Sinha, A. Atmospheric ring-closure and dehydration reactions of 1,4-hydroxycarbonyls in the gas phase: The impact of catalysts. *J. Phys. Chem. A* **2021**, *125*, 5963-5975.
2. Arathala, P.; Musah, R. A. Theoretical studies of the gas-phase reactions of *S*-methyl methanesulfinothioate (dimethyl thiosulfinate) with OH and Cl radicals: Reaction mechanisms, energetics, and kinetics. *J. Phys. Chem. A* **2019,** *123*, 8448-8459.
3. Bunkan, A. J. C.; Hetzler, J.; Mikoviny, T.; Wisthaler, A.; Nielsen, C. J.; Olzmann, M. The Reactions of N-Methylformamide and *N*, *N*-Dimethylformamide with OH and their photo-oxidation under atmospheric conditions: Experimental and theoretical studies. *Phys. Chem. Chem. Phys.* **2015**, *17*, 7046-7059.
4. Jasper, A. W., Miller, J. A. Lennard–Jones parameters for combustion and chemical kinetics modeling from full-dimensional intermolecular potentials. *Combust. Flame.* **2014**, *161*, 101-110.
5. Glowacki, D. R.; Liang, C.-H.; Morley, C.; Pilling, M. J.; Robertson, S. H. MESMER: An open-source master equation solver for multi-energy well reactions. *J. Phys. Chem. A* **2012**, *116*, 9545-9560.
6. Miller, W. H. Tunneling corrections to unimolecular rate constants, with application to formaldehyde. *J. Am. Chem. Soc.* **1979**, *101*, 6810-6814.
